# Mapping of N−C Bond Formation from a Series of Crystalline Peri‐Substituted Naphthalenes by Charge Density and Solid‐State NMR Methodologies

**DOI:** 10.1002/anie.202111100

**Published:** 2021-10-01

**Authors:** Gregory J. Rees, Mateusz B. Pitak, Alberth Lari, Stephen P. Day, Jonathan R. Yates, Peter Gierth, Kristian Barnsley, Mark E. Smith, Simon J. Coles, John V. Hanna, John D. Wallis

**Affiliations:** ^1^ Department of Physics University of Warwick Gibbet Hill Road Coventry CV4 7AL UK; ^2^ Department of Materials University of Oxford Parks Rd Oxford OX1 3PH UK; ^3^ School of Chemistry University of Southampton Highfield Southampton SO17 1BJ UK; ^4^ School of Science and Technology Nottingham Trent University Clifton Lane Nottingham NG11 8NS UK; ^5^ Bruker (UK) Ltd Banner Lane Coventry CV4 9GH UK; ^6^ Vice-Chancellor's Office University of Southampton Highfield Southampton SO17 1BJ UK

**Keywords:** bond theory, NMR spectroscopy, through-bond interaction, through-space interaction, X-ray diffraction

## Abstract

A combination of charge density studies and solid state nuclear magnetic resonance (NMR) ^1^ *J*_NC_ coupling measurements supported by periodic density functional theory (DFT) calculations is used to characterise the transition from an n–π* interaction to bond formation between a nucleophilic nitrogen atom and an electrophilic *sp* ^2^ carbon atom in a series of crystalline peri‐substituted naphthalenes. As the N⋅⋅⋅C distance reduces there is a sharp decrease in the Laplacian derived from increasing charge density between the two groups at ca. N⋅⋅⋅C = 1.8 Å, with the periodic DFT calculations predicting, and heteronuclear spin‐echo NMR measurements confirming, the ^1^ *J*_NC_ couplings of ≈3–6 Hz for long C−N bonds (1.60–1.65 Å), and ^1^ *J*_NC_ couplings of <1 Hz for N⋅⋅⋅C >2.1 Å.

## Graphical abstract

A combination of charge density measurements, solid‐state NMR ^1^ *J*_NC_ couplings, and DFT calculations is presented to characterise the transition from an n–π* interaction to bond formation for a series of model crystalline peri‐substituted naphthalenes.

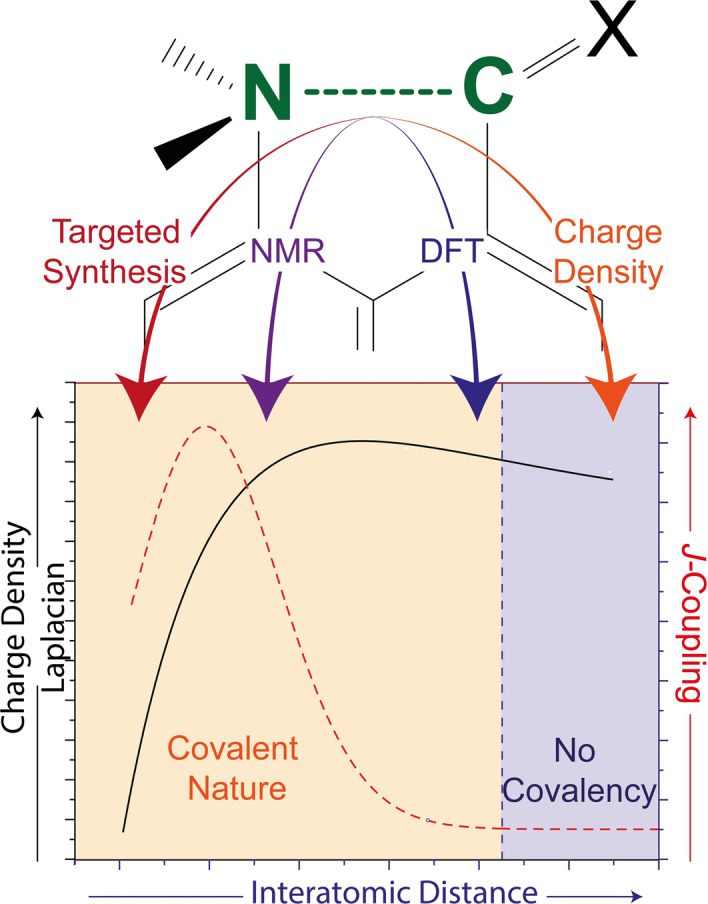

## Introduction

The concept of structure in chemistry implies the existence of bonds that can persist over a range of inter‐nuclear distances until a point is reached where the bond is considered broken.^[1]^ The formation of bonds is central to our understanding of all chemical processes. In this study, we measure the degree of covalent bond formation in a series of crystalline organic compounds using two complementary solid‐state methods, X‐ray crystallography and NMR which are both supported by density functional theory (DFT) calculations.

Interactions between electrophilic and nucleophilic functional groups separated by a range of interatomic distances, measured by single‐crystal X‐ray diffraction, can be considered to represent discrete stages in the reaction between such groups.^[2]^ The concept was first developed using transannular amine‐carbonyl interactions (with interatomic distances ranging from 1.64 to 2.58 Å) in a series of pyrrolizidine alkaloids, such as senkirkine and clivorine (ESI Scheme S1, structures **S1** and **S2**).^[3]^ It was extended to through‐space interactions in peri‐disubstituted naphthalenes between dimethylamino or methoxy groups and a ketone, ester or carboxamide electrophile, where small pyramidalizations of the carbonyl carbons were observed.^[4]^ These peri‐naphthalene systems can alternatively contain methylthio or naphtholate moieties as electron‐rich groups, and alkynes, polarized alkenes or aldehydes as the electron‐deficient centre.[^5^, ^6^] For peri‐naphthalenes bearing a dimethylamino (‐NMe_2_) group adjacent to an electrophilic group containing a multiple bond (such as C=O, C=C, or C≡N), the naphthalene skeleton can hold the groups close to the optimal orientation for orbital overlap, often referred to as the Bürgi–Dunitz angle, inducing an n–π* interaction which can modify the chemistry of the groups.^[4]^ If the electrophile is sufficiently reactive an intramolecular bond is formed.^[7]^ The inter‐group separation can be controlled by adjusting the substituents at the opposing peri positions_._ ^[8]^ n–π* Interactions are particularly important in chemistry, e.g., O⋅⋅⋅C=O interactions between the carbonyl groups is critical in determining conformations of proteins (such as collagen), and the role of n–π* interactions in enzymatic processes is only just being recognised.^[9]^ X‐ray crystallography of model compounds based on peri‐naphthalenes and acenaphthenes have been used to probe the mechanism of nucleophilic attack on silicon and unconventional hydrogen bonding to an amide nitrogen atom.^[10]^

**Figure 1:**
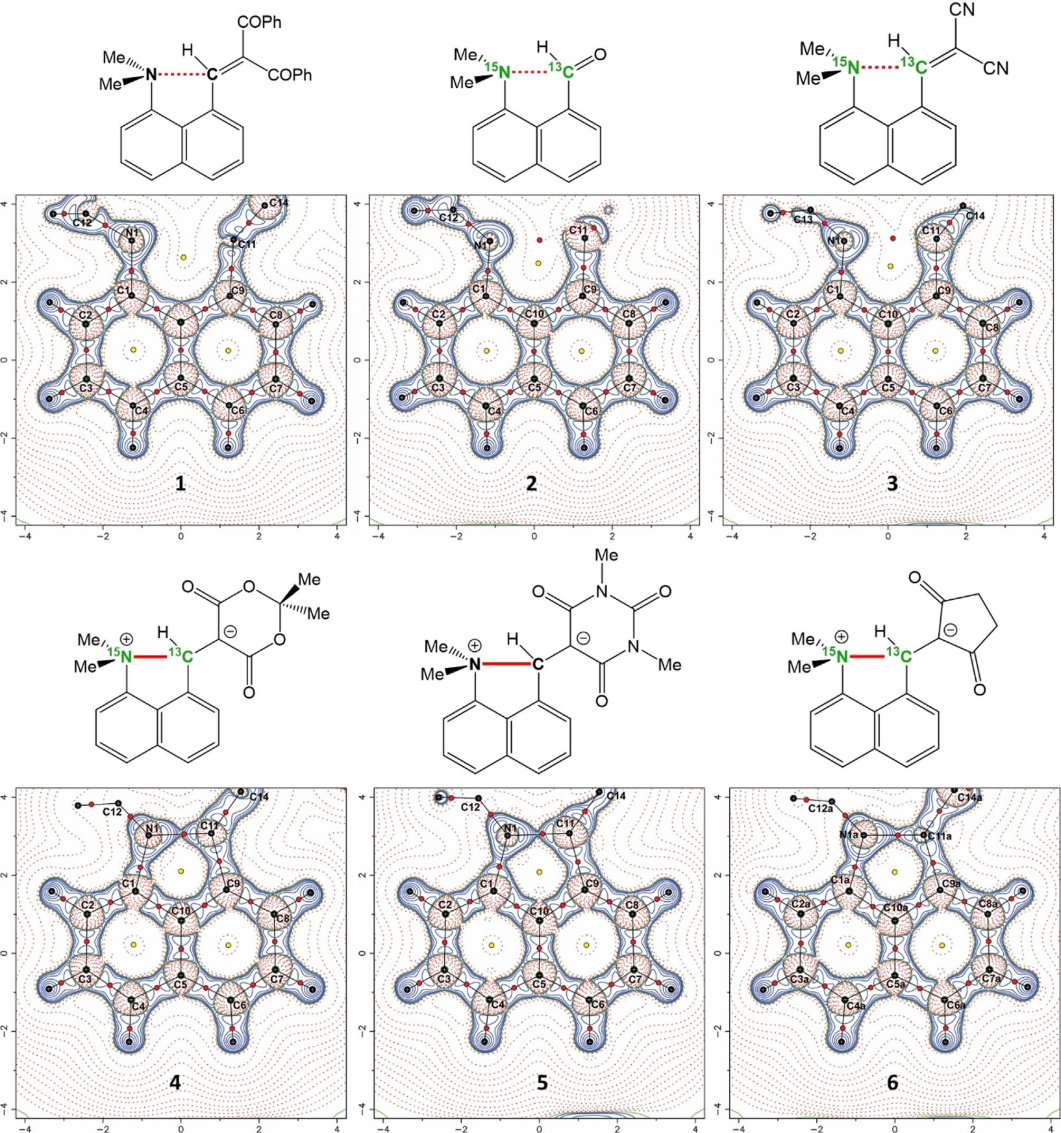
The structures of the *peri*‐substituted naphthalenes **1**–**6** with their Laplacian maps from the charge density determinations. The static deformation charge density distribution maps are given in the ESI. The selectively labelled ^15^N and ^13^C sites, used for NMR studies of **2**, **3**, **4** and **6**, are highlighted in green.

To study the progression from n–π* interaction to initial bond formation we designed a series of six peri‐naphthalenes with a ‐NMe_2_ group situated next either to an aldehyde or various alkenes functionalized with two terminal electron‐attracting groups, outlined in Figure 1, **1**–**6**. X‐ray crystallography shows the Me_2_N⋅⋅⋅C distances decrease as the electrophilic strength of the alkene series increases, with the longest for two benzoyl groups terminating the alkene (**1**; 2.695 Å), then for two nitriles (**3**; 2.413 Å), and finally three with a cyclic link between the terminal groups: a diester (**4**; 1.651 Å), a diamide (**5**; 1.624 Å) and a diketone (**6**; 1.612 and 1.626 Å).[^7^, ^11^] The aldehyde has the second‐longest inter‐atomic distance in the series (**2**; 2.481 Å).^[12]^ Notably, for **4**–**6** the separations correspond to the formation of a particularly long N−C bond (1.612–1.651 Å) which completes a doubly‐fused five‐membered ring in a zwitterionic structure (*cf*. a Me_3_N^+^‐CHRCO_2_^−^ bond: 1.536 Å, and a typical N−C bond between neutral atoms: 1.47 Å).^[13]^ We have probed the development of N⋅⋅⋅C peri‐bonding in this series of crystals using two complementary solid‐state methods:

(1) by determination and topological analysis of the charge densities for the six molecules in this series using accurate, high‐resolution single crystal X‐ray diffraction measurements, which are supported by DFT calculations.

(2) using NMR to directly measure the ^1^ *J*_NC_ coupling between the two nuclei involved in the interaction in the solid state for isotopically enriched molecules **2**, **3**, **4** and **6**.

For the NMR study, the molecules were prepared with ^15^N (60 %) and ^13^C (99 %) isotopic labels at the peri positions (ESI). The crystallographically determined structures are related to the experimentally determined ^1^ *J*_NC_ couplings using periodic DFT calculations to derive the theoretical coupling constants, thus providing a joint solid‐state charge density/NMR approach. The most closely‐related charge density studies on peri‐systems relate to proton sponges and a long (2.700 Å) Me_2_N⋅⋅⋅CONMe_2_ interaction, whilst investigations into Cl⋅⋅⋅Cl interactions and extended hypervalent bonding in S⋅⋅⋅S−S⋅⋅⋅S and S⋅⋅⋅Se−Se⋅⋅⋅S situations have also been completed.[^14^, ^15^] Solid‐state ^1^ *J* coupling studies between peri chalcogenide elements have also been made.^[16]^ However, no combined charge density/^1^J coupling studies on crystalline peri‐naphthalenes have been reported.

## Results and Discussion

Charge density determinations were made from X‐ray diffraction data on high‐quality crystals of **1**–**6** at 100 K, with data for **6** collected using a synchrotron source (Diamond Light Source) on account of small crystal size. Parameters derived from the topological analysis of the charge densities using QTAIM are given in Table 1.^[17]^ Laplacian maps through the molecular plane are shown in Figure 1 with further details, e.g., molecular graphs showing bond paths, and bond and ring critical points, given in the ESI. All structures have a bond (3, −1) critical point between the interacting *peri* N and C atoms, that is a point where the electron density gradient is zero and is a maximum in two and a minimum in one of three orthogonal directions. As the Me_2_N⋅⋅⋅C distance decreases from 2.6758(4) to 1.6070(6) Å, the charge density at this critical point increases dramatically from 0.13 e Å^−3^ in **1**, to 1.29 and 1.35 e Å^−3^ in the two distinct crystallographic environments for the cyclic dione **6**, which is ca. 80 % of the charge density for a typical N^+^−C bond, as observed in the N^+^−Me bonds in **6** (1.55–1.62 e Å^−3^) or seen in tetramethylammonium cations (1.54–1.65 e Å^−3^).^[18]^

**Table 1:** The C⋅⋅⋅N bond distances for **1**–**6** and **S1**–**S3** in order of decreasing Me_2_N⋅⋅⋅C separation, with parameters from the charge density determinations, experimental solid‐state NMR ^15^N and ^13^C isotropic chemical shifts, and the GIPAW‐DFT calculated ^1^ *J*_NC_ couplings and isotropic shifts. The DFT isotropic shifts were determined using *δ*_iso_=−[*σ*_cal_−*σ*_ref_], where *σ*_ref_=170 ppm for ^13^C and −153 ppm for ^15^N.^[19]^

		Charge Density			Experimental MAS NMR		Density Functional Theory (Periodic DFT)			
	Me_2_N⋅⋅⋅C [Å]	Bond path [Å]	*ρ*(r) [e Å^−3^]	∇^2^ *ρ*(r) [e Å^−5^]	*δ*_iso_(^15^N) [ppm]	*δ*_iso_(^13^C) [ppm]	R_ij(N⋅⋅⋅C)_ [Å]	^1^ *J*_NC_ [Hz]	*δ*_iso_(^15^N) [ppm]	*δ*_iso_(^13^C) [ppm]
**1**	2.6758(4)	2.695	0.13	1.69	–	–	2.676	0.85	−328	157
**2**	2.4796(7)	2.481	0.19	1.97	−341	188	2.480	0.48	−335	191
**3**	2.4163(2)	2.418	0.21	2.20	−337	167	2.416	0.45	−318	166
**S1^[20]^**	2.292(4)	2.245	0.33	2.26	–	–	2.292	0.61	−314	183
**S2^[21]^**	1.993(3)	1.993	0.56	2.13	–	–	1.993	2.32	−296	177
**4**	1.6467(5)	1.647	1.19	−2.92	−295	94	1.647	5.88	−285	93
**5**	1.6237(9)	1.624	1.26	−3.46	–	–	1.624	4.01	−282	89
**6**	1.6252(7) 1.6070(6)	1.625 1.607	1.29 1.35	−4.30 −6.73	−292 −292	88 83	1.625 1.607	4.73 3.57	−290 −287	86 82
**S3 200 K**	–	–	–	–	–	–	2.167	1.94	–	–
**100 K^[8]^**	–	–	–	–	–	–	1.750	7.04	–	–

The Laplacian $\nabla_{\rho }^{2}$ , the second derivative of the charge density with respect to distance, highlights areas of charge concentration and depletion, and thus reveals the fine details of the electronic distribution. The Laplacian maps show an increasing spread of charge concentration between the interacting N and C atoms as their separation distance decreases (Figure 1). For **1**–**3**, the charge concentration on the N atom is representative of the lone pair, but for **4**–**6**, there is contact between the charge concentrations on the N and C atoms. The Laplacian values at the bond critical point (BCP) for **1**–**3** are small and positive, indicating closed‐shell interactions, and increase slightly from **1** to **3** (1.69 to 2.20 e Å^−5^), but decrease strongly from **4** to **6** (−2.92 to −6.73 e Å^−5^), indicating the shared nature of these interactions. However, they do not reach the Laplacian values for typical N^+^−C bonds, as seen in the N^+^−Me moieties in **6** (−8.49 to −9.60 e Å^−5^). Together with charge density data, this suggests that even in **6** the N−C bond in the five‐membered ring between peri groups is not fully formed. We note that although in the structural formulae for **4**–**6** the peri N atom is assigned a charge of +1, the Hirshfeld and QTAIM charges derived from the CD study are much smaller (Hirshfeld: 0.00 to +0.12; QTAIM: −0.18 to −0.98), and the Hirshfeld charges contrast with those for **1**–**3,** which are negative (−0.04 to −0.11), due to the more localized electron lone pair.

**Figure 2:**
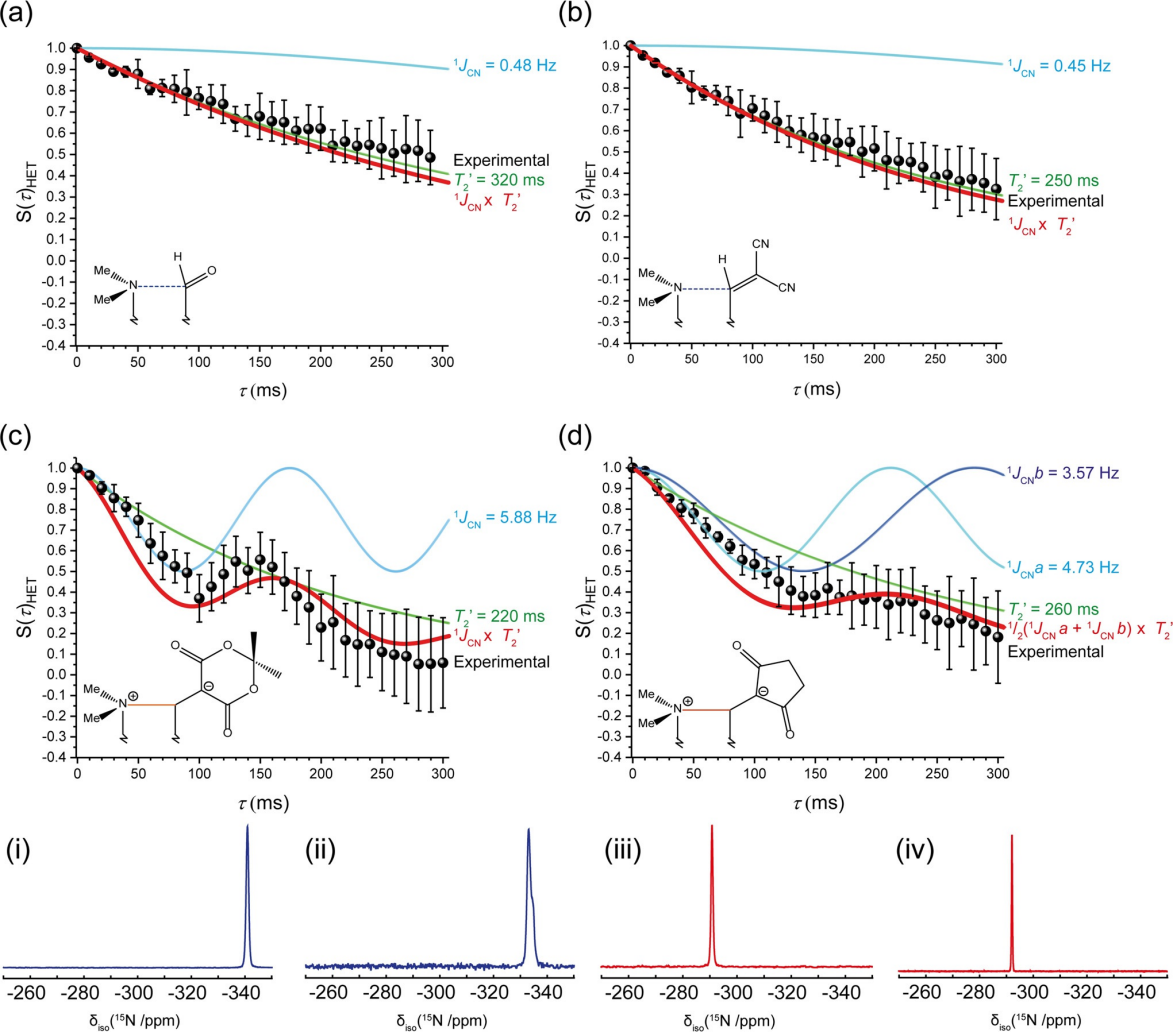
The ^15^N‐^13^C heteronuclear spin‐echo intensities (black circles) of the ^15^N resonance with an increasing tau (*τ*) delay for the naphthalenes a) **2**, b) **3**, c) **4** and d) **6** at 11.75 T (*ν*_0_^1^H=500.1, ^13^C=125.76 and ^15^N=50.69 MHz) and a MAS frequency of (*ν*_R_) 11 kHz. On each graph the GIPAW‐DFT determined Simpson simulated ^1^ *J*_NC_ couplings (cyan), *T*_2_ determined from spin‐echo experiments (green), and their product (red) are given. The decays follow a cos(π*J*_NC_τ)exp(−τ/*T*_2_) function. (i, ii, iii and iv) The respective ^15^N MAS NMR spectra of structures **2**, **3**, **4**, and **6**, those without a bond give a low‐frequency resonance (blue) and after a bond has formed a ≈50 ppm shift to high frequency (red) is observed.

The alkene bond under attack from the dimethylamino group extends its length by ca. 0.12 Å from 1.3509(3) and 1.3659(2) Å in **1** and **3** to 1.4687(6) and 1.4737(6) Å in the two crystallographically independent molecules of **6**, with a decrease in the charge density at the BCP, from 2.28 and 2.25 e Å^−3^ in **1** and **3** to 1.84 and 1.85 e Å^−3^ in **6**. Furthermore, there is a change in the Laplacian from −19.87 and −20.10 e Å^−5^ in **1** and **3** to −14.76 and −14.77 e Å^−5^ in **6**. Given that the typical charge density and Laplacian values for a single C−C bond are ca. 1.6 e Å^−3^ and −10 to −12 e Å^−5^, this suggests that the double bond in **6** has not been fully transformed into a single (*σ*) bond.^[22]^ There is also a change in the ellipticity of this bond, derived from the CD determination (Section 2, ESI), from 0.29–0.32 for **1** and **3**, to 0.13–0.17 for **4**–**6** which could be interpreted as a reduction in the π component of the bonding, though this approach has been questioned since there is no direct connection between the topological analysis and an orbital based description of bonding.^[23]^

**Figure 3:**
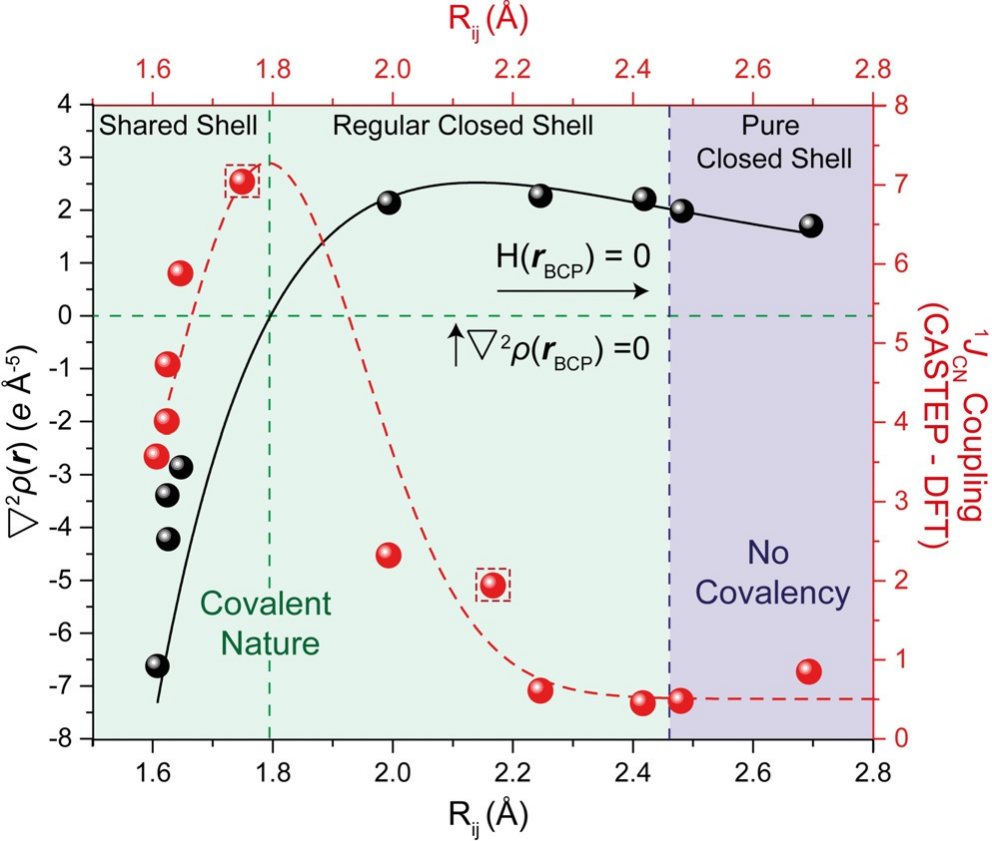
The relationships between the Laplacian of the charge density (∇^2^ *ρ*(r)) and the ^1^ *J*_NC_ coupling against the distance between the interacting N⋅⋅⋅C atoms (R_ij_). The ∇^2^ *ρ*(r)−R_ij_ relationship is fitted to a Morse‐like dependence, as given by Mallinson and Woźniak,^[14]^ ∇^2^p(***r***)=D_e_ [1−exp{−a${}^{\left(R{}_{\mathrm{ij}}-R1\right)}$ }], where D_e_=2.417 and *R*1=2.13867. Accordingly, the DFT‐derived ^1^ *J*_NC_ couplings–R_ij_ relationship is tentatively imposed over a constrained inverse Morse potential, ^1^ *J*_NC_=−D_e_[1−exp{−a${}^{\left(R-R{}_{e}\right)}$ }]^2^, where R_e_ was fixed to ≈1.8 Å and D_e_ is derived from a Harmonic oscillator function to be 7.4 Hz. The function was offset to (^1^ *J*_NC_) 0.5 Hz, the lowest periodic DFT determined ^1^ *J*_NC_ coupling at longer bond lengths. The two calculated ^1^ *J*_NC_ for **S3** at 100 and 200 K are given in red dashed squares.

To provide data for intermediate N⋅⋅⋅C separations, the charge densities for the alkaloids senkirkine and clivorine, **S1** and **S2**, were determined using B3LYP functionals, a 6–311++G** basis set, and atomic coordinates from their reported crystal structures.[^20^, ^21^] At the BCPs for their transannular N⋅⋅⋅C interactions there are small charge concentrations (0.33 and 0.56 e Å^−3^) increasing with decreasing N⋅⋅⋅C separation, and their Laplacian values (2.26 and 2.13 e Å^−5^) are similar to those of the dinitrile **3**. Further calculations were completed for **1**–**6** and reproduced the experimental charge densities at the N⋅⋅⋅C BCP to within 0.2 e Å^−3^ for **1**–**5**, while for **6** the values were within 0.7 e Å^−3^, though the diffraction dataset for **6** is slightly less extensive and complete than for **1**–**5**. The trend of the Laplacians was also reproduced, though with greater divergence (more negative) for **4**–**6** (ESI, Figure S27). The variation of Laplacian with the N⋅⋅⋅C separation for **1**–**6** and **S1** and **S2** (Figure 3), shows a trend which can be fitted with a Morse‐like function, which shows a small rise in Laplacian as the N⋅⋅⋅C distance contracts to ca. 2.1 Å and then a rapid decrease in value, passing through zero at ca. 1.8 Å. Thus, the Laplacian is a sensitive discriminator for the bonding, with the small positive values corresponding to closed‐shell interactions, which decrease strongly for **4**–**6** suggesting covalent interactions. A similar Morse‐like relationship of the Laplacian against interatomic separation was shown by Minor and Woźniak et al. for OH⋅⋅⋅O and NH⋅⋅⋅N hydrogen bond formation.^[24]^ A plot of the calculated energy density (H) at the N−C bond critical point against increasing N⋅⋅⋅C separation tended, from negative, to zero at ca. N⋅⋅⋅C: 2.45 Å, suggesting this is the limit for covalent interaction (ESI). Furthermore, the Delocalisation Index (DI) was calculated for the peri N⋅⋅⋅C interaction/bond in each molecule as a measure of bond order.^[25]^ For **4**–**6**, this was in the range of 0.71–0.72, being lower for **2** (0.14) and **3** (0.19), and very low for **1** (0.08). For **4**, the other three N−C bonds had DI's of 0.84–0.85 consistent with the peri‐bond not being fully formed (ESI, Table S16, Figure S29).

The charge density, determined from X‐ray diffraction measurements, maps the valence electron distributions between the interacting groups. The corresponding crystal structures for **S1**–**S2** and **1**–**6** have been used to calculate, via DFT, the ^1^ *J*_NC_ couplings between ^15^N and ^13^C atoms located at either end of the peri interaction/partial bond to characterise the interaction across the Me_2_N⋅⋅⋅C bridge. This use of DFT to predict the NMR parameters is compared to experimental measurements made on naphthalenes **2**, **3**, **4** and **6** in which the two interacting/bonding atoms are both isotopically labelled (Scheme S1, ESI).

The ^13^C and ^15^N cross‐polarization MAS (CPMAS) spectra of the ^15^N⋅⋅⋅^13^C enriched aldehyde and alkenedinitrile, **2** and **3**, show single resonances from the labelled sites, with *δ*_iso_(^13^C): 188 and 167 ppm (Figure S32, ESI) and *δ*_iso_(^15^N): −341 and −337 ppm (Figure 2, i and ii) respectively. For **4** and **6**, the shorter N⋅⋅⋅C distances reduce the ^13^C shifts to lower values, *δ*_iso_: 94 ppm (**4**) and *δ*_iso_: 88 and 83 ppm (for crystallographically independent molecules of **6**) consistent with the formation of N−C bonding and an *sp* ^3^ carbon centre. For ^15^N, which has a negative gyromagnetic ratio, the shifts increase accordingly to *δ*_iso_ −295 and −292 ppm for **4** and **6**, respectively, with no differentiation between the two independent molecules of **6**, consistent with the formation of a more deshielded nitrogen nuclei (Figure 2, iii and iv). The chemical shift is a large NMR interaction (kHz) and is highly sensitive to changes in bonding and the local environment, however, it only indirectly suggests an N−C bond has formed.

The presence of a measurable ^1^ *J*_NC_ coupling at the ^15^N nucleus would indicate there is an electron‐mediated through‐ bond two nuclear spin interaction across the Me_2_N⋅⋅⋅C bridge, which has traditionally been used to characterize and quantify bonding environments.[^26^, ^27^] Solid‐state NMR experiments seldom succeed in achieving the spectral resolution observed in the solution state because of the existence of high order cross terms which cannot be reduced by MAS, residual dipolar couplings, and the distribution of isotropic shifts due to non‐perfect ordering or π‐stacking faults.^[28]^ A mechanism for measuring the ^1^ *J* coupling in the solid‐state is to utilise the spin‐echo sequence which refocuses the evolution of all the terms that appear as offsets, in particular those that are caused by a distribution of chemical shifts. The use of spin echoes in an NMR experiment allows the detection of chemical shift separated *J* couplings even when inhomogeneous broadening means it is not directly visible in the observed spectrum.^[29]^ The presence of other highly coupled nuclei with large quadrupolar couplings (such as ^14^N or ^17^O) can also cause dephasing of the signal and prevent accurate measurements of the *J*‐coupling, therefore the measurement was observed from the perspective of the ^15^N nuclei which is solely coupled to 99 % ^13^C (*I*=1/2 ), whereas the more sensitive ^13^C is coupled to 60 % ^15^N (*I*=1/2 ) and 40 % quadrupolar ^14^N (*I*=1), which would cause greater dephasing and overestimation of the ^1^ *J* coupling. We have previously discussed a methodology of utilizing periodic DFT calculations on molecular structures determined by single‐crystal X‐ray crystallography to determine ^1^ *J* couplings which are validated with spin‐echo solid‐state NMR experiments. Here, we expand this methodology to compare the charge density maps with NMR observations.^[29]^

The heteronuclear ^15^N‐^13^C spin‐echo decay for the aldehyde **2** and dinitrile **3** (Figure 2 a and b) both show very shallow exponential decays which are indicative of narrow resonances that are not broadened by ^1^ *J*_NC_ coupling contributions. A green simulated fit is given for the *T*_2_ decays, with the blue SIMPSON^[30]^ simulation of the periodic DFT determined ^1^ *J*_NC_ coupling, and their product is given in red. The observed decay can be reliably fitted to both the *T*_2_ component (determined from a homonuclear spin‐echo) and *T*_2_ with the minor 0.48 or 0.45 Hz ^1^ *J*_NC_ coupling. The origin of these small ^1^ *J*_NC_ couplings is attributed to the weak bonding interaction between the two nuclei.

The zwitterions **4** and **6** both show a more dramatic exponential decay, which cannot solely be attributed to the measured *T*_2_ relaxation. The GIPAW‐DFT ^1^ *J*_NC_ calculations predict more substantial couplings of 5.88 Hz for **4** and an average of 4.15 Hz for **6**. When the cosine of these couplings is multiplied by the exponent of the *T*_2_ decay, the red projections (Figure 2 c and d) are observed, which are in good agreement with the spin‐echo data. The dione **6** has two crystallographically distinct molecules, with Me_2_N⋅⋅⋅C distances of 1.607 and 1.625 Å, resulting in ^1^ *J*_NC_ couplings of 3.57 and 4.73 Hz, respectively. It is presumed both sites will give an equal contribution to the spin echo, thus a ^1^ *J*_NC_ coupling of 4.15 Hz was utilised in the simulation. Unfortunately, the summation of the two offset ^1^ *J*_NC_ components dampens the cosine feature of the decay, giving a less accurate fit. Concurrent periodic‐DFT calculations were performed on two structures arising from variable temperature studies on a substituted derivative of **3** (**S3**, ESI), which have been very recently reported, to provide ^1^ *J*_NC_ couplings in the Me_2_N⋅⋅⋅C: 1.7–2.4 Å region.^[8]^ In this unique case the Me_2_N⋅⋅⋅C separation at 200 K is 2.167(4) Å, but contracts, in a reversible process, to 1.749(3) Å at 100 K giving calculated ^1^ *J*_NC_ values of 1.94 and 7.04 Hz, respectively.

In Figure 3, the N−C distance (R_ij_) is plotted against both the second derivative of the charge density and the periodic DFT‐derived, NMR confirmed, ^1^ *J*_NC_ coupling constants (Hz) for **1**–**6** and **S1**–**S3**. A negative second derivative of the electron density is representative of a shared shell covalent bond which is observed below 1.8 Å. Between 1.8 and 2.45 Å a positive second derivative of the electron density is observed, which is still covalent in nature but is defined as regular closed‐shell, and at longer bonds lengths (>2.45 Å) no covalency is observed and a pure closed‐shell bond arrangement dominates. The corresponding ^1^ *J*_NC_ coupling results can be *tentatively* fitted to an inverse Morse function (Figure 3, red dashed line), in contrast to the Morse‐like dependence outlined by charge density. It is interesting to note that though the ^1^ *J*_NC_ values are larger for **4**–**6** with high delocalization indices for the peri N−C bond, and very small for **1**–**3**, there is a maximum in the value of ^1^ *J*_NC_ (ca. 7.4 Hz) which occurs at an N⋅⋅⋅C separation of ca. 1.79 Å. Indeed, the maximum coincides with the point where the Laplacian of the charge density in the bond begins to strongly decrease as the N⋅⋅⋅C distance closes. Above an N⋅⋅⋅C separation of 2.45 Å, corresponding to a calculated energy density of zero at the bond critical point, the coupling remains constant at a minimum value of 0.5 Hz, consistent with no significant covalency between the two atoms.

## Conclusion

We have presented an advanced NMR crystallography methodology that allows DFT‐driven observables and experimental charge density measurements to be utilised to determine the conditions required for bond formation in a series of model compounds containing a Me_2_N⋅⋅⋅C(*sp* ^2^) interaction or partially formed bond. For this model system, as confirmed by DFT corroborated charge density and NMR observations, the variation of Laplacians and ^1^ *J*_NC_ coupling constants suggest that at an N⋅⋅⋅C separation of ca. 1.8 Å, the system starts to form a covalent shared shell bond, with an increasing degree of formation as the atomic separation reduces further. This approach opens up the possibility of identifying the transition state for bond formation, of importance, for example, for understanding enzyme catalyzed conjugate additions.^[31]^

For additional bond formation studies, a more computational approach could be taken. Models for different reaction stages could be obtained using calculations on various substituted *peri*‐naphthalene compounds, with the N⋅⋅⋅C interaction distance constrained to desirable distances in the 1.5–2.5 Å range but allowing full relaxation of the rest of the structure. From each such structure QTAIM parameters and ^1^ *J*‐couplings could be derived. Modelling of crystal structures may also assist, for example, the tolerance of a N⋅⋅⋅C interaction within the 1.7–2.3 A range, for example, in **S3**, may be partly due to other attractive interactions such as the hydrogen bonding in the crystal packing.^[8]^ Furthermore, computational methodologies, such as random structure searching, can identify new synthetic targets with desired N⋅⋅⋅C distances.^[32]^ To summarise, we have illustrated here that charge density, NMR and DFT methods can be used together to characterize bond formation, a process which is at the heart of chemistry.

## Conflict of interest

The authors declare no conflict of interest.

## Supporting information

As a service to our authors and readers, this journal provides supporting information supplied by the authors. Such materials are peer reviewed and may be re‐organized for online delivery, but are not copy‐edited or typeset. Technical support issues arising from supporting information (other than missing files) should be addressed to the authors.

Supporting Information
