# Supplementary material for: Mapping of N−C Bond Formation from a Series of Crystalline Peri‐Substituted Naphthalenes by Charge Density and Solid‐State NMR Methodologies
Source: Angew Chem Int Ed Engl. 2021 Oct 1;60(44):23878–84. doi: 10.1002/anie.202111100 (PMC8596510; doi:10.1002/anie.202111100)
Supplement: Supplementary file 1 — Supporting Information [file ANIE-60-23878-s001.pdf]

Angewandte Chemie
Eine Zeitschrift der Gesellschaft Deutscher Chemiker

Supporting Information

Mapping of N−C Bond Formation from a Series of Crystalline Peri-Substituted Naphthalenes by Charge Density and Solid-State NMR Methodologies

Gregory J. Rees, Mateusz B. Pitak, Alberth Lari, Stephen P. Day, Jonathan R. Yates, Peter Gierth, Kristian Barnsley, Mark E. Smith, Simon J. Coles, John V. Hanna,* and John D. Wallis**

anie_202111100_sm_miscellaneous_information.pdf

Structures of S1-S3. **Page 1.**

Experimental methodology:

1. **Synthesis of doubly labelled compounds.** **Page 2.**
2. **Details of charge density measurements.** **Page 5.**
3. **Details of calculations.** **Page 35.**
4. **Details of solid-state NMR measurements.** **Page 37.**
5. **References.** **Page 39.**

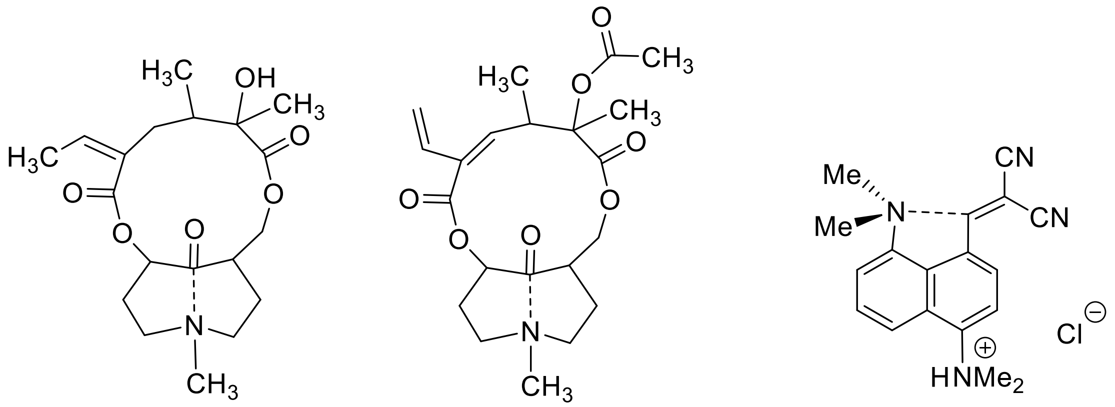

S1 **S2** **S3**

Figure S1. Structures of alkaloids Senkirkine **S1** and Clivorine **S2** and the HCl salt of the 4',6'-bis(dimethylamino)naphthyl-2-cyano-propenenitrile, **S3**.

Experimental Methodology.

1. Synthesis of 1-6 and doubly labelled 2-4 and 6.

General scheme for the synthesis of 1-6.

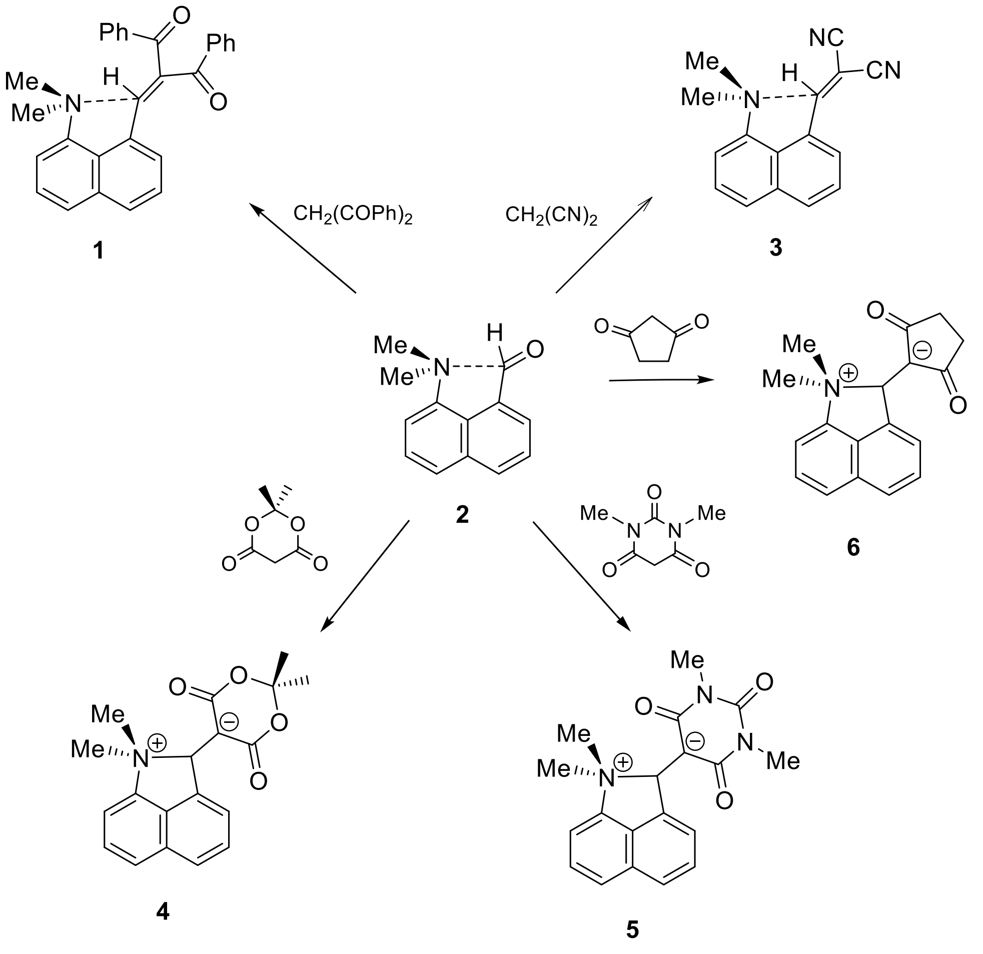

Scheme S1. Synthesis of compounds, **1**-**6**[S1-S2] using methods described below.

General. The solution NMR characterisation was completed using a JEOL ECLIPSE 400 spectrometer operating at ^{1}H, ^{13}C and ^{15}N Larmor frequencies of 400, 100.6 and 40.6 MHz respectively, or an 11.7 T Bruker Avance HD NMR spectrometer, operating at ^{1}H, ^{13}C and ^{15}N Larmor frequencies of 500.1, 125.9 and 50.8 MHz respectively, using $CDCl_3$ as solvent and tetramethylsilane (TMS) as standard (δ_{iso} = 0 ppm) unless otherwise stated. The ^{15}N NMR spectra in solution are measured relative to nitromethane. The IR spectra were recorded on a Perkin Elmer Spectrum 100 FT-IR Spectrometer using Attenuated Total Reflection sampling unless otherwise stated, and are reported in cm^{-1}. Mass spectra were recorded at the EPSRC Mass Spectrometry Centre at the University of Swansea on an LTQ Orbitrap spectrometer or NTU using an Agilent 6890N Network GC System equipped with a 5975 Inert XL Mass Selective Detector. Flash chromatography was performed on 40–63 silica gel (Merck).

Synthesis of ^{15}N and ^{13}C labelled 2-4 and 6.

To completed ^{1}J solid state NMR studies on the interaction between *peri* groups, compounds **2-4** and **6** were prepared to bear ^{15}N and ^{13}C labels at the *peri* positions (Scheme S2). Naphthalene was nitrated using $K^{15}NO_3$ (^{15}N: 60%) and concentrated sulphuric acid[S1] to give 1-(^{15}N)-nitronaphthalene (^{15}N)-**S4** in 74 % yield after chromatography. The product was reduced to 1-(^{15}N)-aminonaphthalene and methylated to give the dimethylamino compound (^{15}N)-**S5** in 64% yield after chromatography. The doubly labelled material was obtained by lithiation of (^{15}N)-S**5** with excess *n*-butyl lithium over 5 days, removal of unreacted *n*-butyl lithium from the precipitated lithiated material, and reaction with 1.1 equivalents of Me_2N-^{13}CHO (^{13}C: 99%). Purification by chromatography gave (^{15}N^{13}C)-**2** in 56 % yield. (^{15}N^{13}C)-**3**, -**4** and -**6** were prepared by Knoevenagel condensations on (^{15}N^{13}C)-**5** with malononitrile, Meldrum's acid and cyclopenta-1,3-dione respectively.[S1-S2]

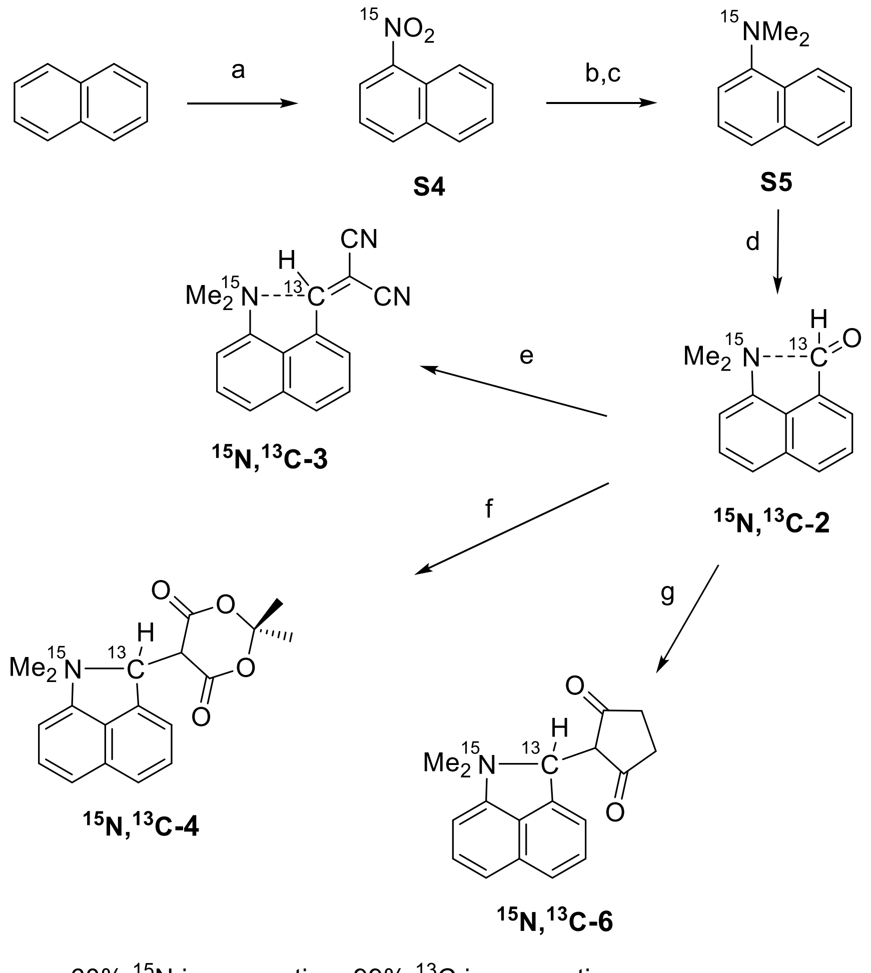

60% ^{15}N incorporation; 99% ^{13}C incorporation.

Scheme S2. Synthetic scheme for preparing isotopically labelled **2**-**4** and **6**. a, $K^{15}NO_3$ / H_2SO_4 / 60°C, chromatography; b, H_2 / Pd-C (10%) / ethanol, chromatography; c KOH / MeI / DMSO, 20 ^{o}C chromatography; d, *n*-BuLi / ether / 5 days, Me_2N^{13}CHO, MeOH, chromatography; e, $CH_2(CN)_2$ / methanol / ethylenediammonium *bis*(acetate) catalyst / reflux; f, Meldrum's acid, DMSO, 25 ^{o}C; g, cyclopentane-1,3-dione / methanol / ethylenediammonium *bis*(acetate) catalyst / reflux.

Synthesis of 1-(^{15}N,60%)-dimethylaminonaphthalene-8-(^{13}C,99%)carbaldehyde, ^{15}N,^{13}C-2.

n-Butyl lithium (20 ml , 2.5 M in hexane, 50 mmol) was added to a solution of 1-(^{15}N, 60%)-**S5** (2.20 g, 12.8 mmol) in dry ether (25 ml) under nitrogen and stirred for 5 days during which time a lemon precipitate of lithiated material formed.[S2] The solvent containing excess lithiating agent was removed by syringe, and the remaining solid washed with dry ether (3 x 20 ml). The solid was stirred with dry ether, cooled to -78 ^{o}C and treated with dimethylformamide containing a 99%^{13}C labelled carbonyl group (1.00 g, 13.5 mmol). After allowing to warm to room temperature overnight, the reaction mixture was treated with methanol (15 ml), stirred for 30 min and diluted with ether (80 ml). The solution was extracted with water (3 x 50 ml) and brine (1 x 50 ml) and dried over $MgSO_4$. Flash chromatography of the residue using hexane/ether (3:1) gave the product **^{15}N,^{13}C-2** as a pale yellow solid (1.45 g, 56.4%), R_f=0.28 (hexane/ether 3:1); m.p. 85-86 ^{o}C; ^{1}H NMR (600.1 MHz, $CDCl_3$, 20 ^{o}C, TMS): δ=10.66 (d, ^{1}J(C,H) = 186.9 Hz, 1H; -^{13}C*H*O), 7.92 (d, ^{3}J(H,H) = 7.1 Hz, 1H; 7-*H*), 7.67 (d, ^{3}J(H,H) = 7.6 Hz, 1H; 5-*H*), 7.56 (d, ^{3}J(H,H) = 7.1 Hz, 1H; 4-*H*), 7.52 (t, ^{3}J(H,H) = 8.0 Hz, 1H) and 7.51 (t, ^{3}J(H,H) = 8.0 Hz, 1H) (3-,6-*H*), 7.35 (d, ^{3}J(H,H) = 7.3 Hz, 1H; 2-*H*), 2.74 (s, 6H; N($CH_3)_2$); ^{13}C NMR (150.9 MHz, $CDCl_3$, 20 K, TMS): δ=191.7 (s,^{13}*C*HO); ^{15}N NMR (60.8 MHz, $CDCl_3$, 20 K, CH_3NO_2): δ= -338.5 (s, *N*($CH_3)_2$); IR (ATR): $\tilde{\nu}$= 2943, 2865, 2831, 2790, 1622 (^{13}C=O), 1611 (^{13}C=O), 1505, 1471, 1449, 1429, 1359, 1345, 1204, 1171, 1101, 1089, 1040, 1023, 1006, 955, 888, 843, 801, 784, 767, 713, 660 cm^{-1}; *cf.* unlabelled material: 1658 (C=O) cm^{-1}; *m/z:* EI 202 (4.4%), 201 ([M(^{13}C, ^{15}N)]$^{+}$, 32.8%), 200 ([M(^{13}C)]$^{+}$, 21.7%), 172 (51.5%), 171 (100%), 170 (53.7%); HRMS: (LTQ Orbitrap XL, NSI): 203.1102 (12%), 202.1069 (100%) and 201.1099 (12%) [$^{13}CC_{12}H_{13}{}^{15}NO$ + H]$^{+}$ requires 202.1074, [$^{13}CC_{12}H_{13}NO$ + H]$^{+}$ requires 201.1103.

Synthesis of 1-(1’-(^{15}N,60%)-dimethylaminonaphth-8’-yl)-1-(^{13}C,99%)-ethene-2,2-dinitrile ^{15}N,^{13}C-3.

The doubly labelled aldehyde **^{15}N,^{13}C-2** (32 mg, 0.16 mmol) and malonitrile (103 mg, 1.56 mmol) were dissolved in DMSO (1.5 ml) and then left to stir at room temperature for 72 h. The solution was treated with water (10 ml) and left at 0°C for 36 h. The precipitate was filtered off, washed with water, and dried in a desiccator to give the dinitrile **^{15}N,^{13}C-3** as a pale green solid (20 mg, 50%) R_f=0.62 (cyclohexane/ethyl acetate 3:1); m.p. 153-155 ^{o}C ; ^{1}H NMR (600.1 MHz, $CDCl_3$, 20 ^{o}C, TMS): δ=8.78 (d, ^{1}J(C,H) = 173.5 Hz, 1H; 8-^{13}C*H*=), 7.94 (d, ^{3}J(H,H) = 8.4 Hz, 1H; 7-*H*), 7.72 (d, ^{3}J(H,H) = 8.1 Hz, 1H; 5-*H*), 7.57 (t, ^{3}J(H,H) = 7.9 Hz, 1H) and 7.56 (t, ^{3}J(H,H) = 7.9 Hz, 1H) (3-,6-*H*), 7.51 (d, ^{3}J(H,H) = 6.2 Hz, 1H; 4-*H*), 7.48 (d, ^{3}J(H,H) = 8.1 Hz, 1H; 2-*H*), 2.73 (s, 6H; N($CH_3)_2$); ^{13}C NMR (150.9 MHz, $CDCl_3$, 20 ^{o}C, TMS): δ=165.7 (s, (8’-^{13}*C*H=); ^{15}N NMR (60.8 MHz, $CDCl_3$, 20 ^{o}C, CH_3NO_2): δ= -337.3 (s, *N*($CH_3)_2$); IR (ATR): $\tilde{\nu}$= 2938, 2832, 2225 (C≡N), 1521, 1474, 1451, 1371, 1037, 891, 835, 786, 775, 749, 620, 581 cm^{-1}; HRMS: (LTQ Orbitrap XL, NSI): 250.1190 (100%) and 249.1221 (60%). [$^{13}CC_{15}H_{13}{}^{15}NN_2$ + H]$^{+}$ requires 250.1186, [$^{13}CC_{15}H_{13}N_3$ + H]$^{+}$ requires 249.1216.

Synthesis of (1’-(^{15}N,60%)-1’,2’-Dihydro-1’,1’-dimethylbenzo[cd]indol-1’-ium-2’-yl) 2-(^{13}C,99%)-4,6- dioxo-1,3-dioxan-5-ide, ^{15}N,^{13}C-4.

Doubly labelled aldehyde **^{15}N,^{13}C-2** (102 mg, 0.51mmol) and Meldrum’s acid (77 mg, 0.53 mmol) were stirred together in dry DMSO (2 ml) under nitrogen at room temperature for 48 h. The reaction mixture was poured into distilled water (20 ml), and left at 0°C for 24 h. The precipitate was collected, washed with water, and dried in a desiccator to give product **^{15}N,^{13}C-4** as white crystals (117 mg, 70%). R_f=0.53 (ethyl acetate/methanol 1:1); m.p. 191-193 ^{o}C; ^{1}H NMR (600.1 MHz, $CDCl_3$, 20 ^{o}C, TMS): δ=7.93 (d, ^{3}J(H,H) = 8.3 Hz, 1H; Ar-H_1), 7.83 (d, ^{3}J(H,H) = 8.0 Hz, 1H; Ar-H_1), 7.70 (t, ^{3}J(H,H) = 7.6 Hz, 1H; Ar-H_1), 7.61 (t, ^{3}J(H,H) = 7.6 Hz, 1H; Ar-H_1), 7.41 (d, ^{3}J(H,H) = 8.0 Hz, 1H; Ar-H_1), 7.32 (d, ^{3}J(H,H) = 7.0 Hz, Ar-H_1), 7.10 (d, ^{1}J(C,H) = 152.1 Hz, 1H; 2’-*H*), 3.40 (s, 6H; N($CH_3)_2$), 1.83 (s, (6H, C($CH_3)_2$); ^{13}C NMR (150.9 MHz, $CDCl_3$, 20 ^{o}C, TMS): δ=99.68 and 99.72 (2 x s, 2’-*C adjacent to ^{14}N and ^{15}N*) **;** ^{15}N NMR (60.8 MHz, $CDCl_3$, 20 ^{o}C, CH_3NO_2): δ= -292.9 (s, *N*($CH_3)_2$). At room temperature this molecule rapidly opens and closes the *peri* bond leading to an average structure and thus no coupling is observed between

peri atoms.[S2] IR (ATR): $\tilde{\nu}$= 3041, 3025, 2999, 2924, 1684, 1615 (C=O), 1460, 1432, 1394, 1366, 1307, 1277, 1251, 1202, 1180, 1164, 1129, 1074, 1004, 932, 899, 824, 788, 769, 742, 655, 636, 612 cm^{-1}; HRMS: (LTQ Orbitrap XL, NSI): 328.1392 (100%) and 327.1421 (64%). [$^{13}CC_{18}H_{19}{}^{15}NO_4$+H]$^+$ requires 328.1391, [$^{13}CC_{18}H_{19}NO_4$+H]$^+$ requires 327.1420.

Synthesis of 1'-(^{15}N,60%)-1',2'-Dihydro-1',1'-dimethylbenzo[cd]indol-1'-ium-2'-yl 1-(^{13}C,99%) -2 ,5-dioxo-cyclopentan-1-ide monohydrate ^{15}N,^{13}C-6.

Doubly labelled aldehyde **^{15}N,^{13}C-2** (250 mg, 1.26 mmol) and cyclopentan-1,3-dione (125 mg, 1.28 mmol) were refluxed together in methanol (8 ml) with ethylenediamine diacetate (10 mg) for 3 h. Evaporation of most of the solvent, and addition of ether gave the product **^{15}N,^{13}C-6** as a cream powder (202 mg, 58%). R_f=0.13 (ethyl acetate/methanol 1:1); m.p. 137-139 °C; ^{1}H NMR (600.1 MHz, CDCl$_3$, 20 °C, TMS): δ=7.94 (d, 1H, ^3J(H,H) = 8.8 Hz, 1H; Ar-H_1), 7.83 (d, ^3J(H,H) = 8.8 Hz, 1H; Ar-H_1), 7.70 (t, ^3J(H,H) = 8.1 Hz, 1H; Ar-H_1), 7.61 (t, ^3J(H,H) = 8.1 Hz, 1H; Ar-H_1), 7.41 (d, ^3J(H,H) = 7.4 Hz, 1H; Ar-H_1), 7.26 (d, ^3J(H,H) = 7.4 Hz, 1H; Ar-H_1), 6.59 (d, ^1J(C,H) = 147.9 Hz, 1H; 2'-*H*), 3.36 (s, 6H; N(CH_3)$_2$), 2.54 (s, 4H; 3-,4-H_2); ^{13}C NMR (150.9 MHz, CDCl$_3$, 20 °C, TMS): δ=88.79 and 88.82 (2 x s, 2'-*C adjacent to ^{14}N and ^{15}N*); ^{15}N NMR (60.8 MHz, CDCl$_3$, 20 °C, CH$_3$NO$_2$): δ= -293.3 (s, *N*(CH$_3$)$_2$); At room temperature this molecule rapidly opens and closes the *peri* bond leading to an average structure and thus no coupling is observed between peri atoms.[S2] IR (ATR): $\tilde{\nu}$= 3404 br, 3176, 3018, 2909, 1547 (C=O), 1498, 1460, 1444, 1434, 1427, 1369, 1343, 1296, 1279, 1258, 1234, 1022, 988, 901, 832, 825, 778, 742, 698, 641, 608 cm^{-1}; HRMS: (LTQ Orbitrap XL, NSI): 282.1339 (100%) and 281.1362 (59%). [$^{13}CC_{17}H_{17}{}^{15}NO_2$+H]$^+$ requires 282.1336, [$^{13}CC_{17}H_{17}NO_2$+H]$^+$ requires 281.1366.

2. Single Crystal X-ray Diffraction and Charge Density Measurements.

Topological analysis of charge density distributions using Bader's Atom in Molecules approach (AIM)[S3] is well established and now used to explore weaker types of bonding and interactions, for example, hydrogen bonding, halogen bonding, O···O and O···Br interactions and also systems containing anions and receptors[S4]. To probe the development of N-C bonding between the *peri*-groups in **1**-**6**, a charge density determination and analysis has been carried out on a high-quality single crystal of each substance. Highly redundant high-resolution X-ray data ($\sin\theta/\lambda$ >1.0 Å^{-1}) of **2** and **4** were carefully collected on a Nonius KappaCCD area-detector diffractometer located at the window of Nonius FR591 rotating-anode X-ray generator, equipped with a molybdenum target ($\lambda_{MoK\alpha}$ = 0.71073 Å) and Roper CCD camera,[S5] whereas for **1** and **5** X-ray intensities were collected on a *Rigaku AFC12* goniometer equipped with an enhanced sensitivity (HG) *Saturn724+* detector mounted at the window of an *FR-E+ SuperBright* (Mo Kα, λ = 0.71073 Å) rotating anode generator with HF *Varimax* optics (100µm focus),[S6] and processed with the CrystalClear[S7] software package. Crystals were cooled using an Oxford Cryosystems Cobra system. X-ray diffraction data from **3** were collected on Bruker Nonius X8 Apex diffractometer equipped with a Kryoflex cooling device and a data collection strategy for the high-resolution data set was determined using COSMATIC and COSMO.[S8] Diffraction data for **6** were collected in Experimental Hutch 1 (EH1) on the I19 beamline at the Diamond Light Source, which is equipped with a Crystal Logic 4-circle kappa geometry diffractometer and a Rigaku Saturn 724+ CCD detector. The sample was cooled to and maintained at 100 K using an Oxford Cryosystems Cryostream Plus device and diffractometer control and data processing were carried out using the CrystalClear software.[S7] The data integrations for **1-2** and **4-5** were performed with EvalCCD[S9] and SORTAV[S10] was used to apply a Gaussian absorption correction and to average and merge the sets of intensities. The crystal structures were all solved by direct methods and the hydrogen atoms located on carbon atoms were placed at calculated positions. Least squares independent atom refinement (IAM) was carried out with the SHELX-

2014[S11] software package. All the non-hydrogen atoms were refined with anisotropic displacement parameters, whereas all hydrogen atoms were refined using a riding model on their parent atoms with isotropic displacement parameters based on the equivalent isotropic displacement parameter (U_{iso}(H) = 1.2 $U_{eq(aromatic)}$ and U_{iso}(H) = 1.5 $U_{eq(CH3)}$) of the parent atom.

Table S1. Crystallographic data for **1-6**.

	**1**	**2**	**3**	**4**	**5**	**6**
Formula	$C_{28}H_{23}NO_2$	$C_{13}H_{13}NO$	$C_{16}H_{13}N_3$	$C_{19}H_{19}NO_4$	$C_{19}H_{19}N_3O_3$	$C_{18}H_{17}NO_2.H_2O$
Formula weight	405.47	199.24	247.29	325.36	337.37	297.34
Crystal system	Monoclinic	Monoclinic	Monoclinic	Monoclinic	Triclinic	Monoclinic
Space group	$P2_1/n$	$P2_1/c$	$P2_1/c$	$P2_1/c$	P-1	$P2_1/c$
a[Å]	11.2313(8)	8.3752(17)	11.0721(5)	9.9080(1)	8.324(4)	18.559(3)
b[Å]	11.4392(8)	10.4610(19)	9.0361(4)	12.6780(1)	8.715(4)	9.0934(11)
c[Å]	16.7547(11)	12.628(3)	13.6278(6)	12.2500(1)	12.082(6)	19.555(3)
α[°]	90	90	90	90	89.54(3)	90
β [°]	93.893(3)	109.326(3)	103.765(2)	91.119(1)	85.32(3)	112.659(2)
γ[°]	90	90	90	90	65.260(12)	90
V [Å^3]	2147.6(3)	1044.0(4)	1324.28(10)	1538.47(2)	793.0(7)	3045.5(8)
Z	4	4	4	4	2	8
ρ [g cm^{-3}]	1.254	1.280	1.240	1.405	1.413	1.297
T [*K*]	100(2)	100(2)	100(2)	100(2)	100(2)	100(2)
λ (Å)	0.71073	0.71073	0.71073	0.71073	0.71073	0.71073
μ (mm^{-1})	0.078	0.081	0.076	0.099	0.098	0.088
$(\sin\theta/\lambda)_{max}$ [Å^{-1}]	1.182	1.237	1.148	0.999	1.208	1.016
no. of unique reflections	27914	16402	15778	12725	22501	27188
no of reflections with I > 2σI	19062	15722	12771	9329	20498	24211
Data completeness to θ_{max} ° [%]	93.9	98.9	94.0	98.7	96.2	92.5

Continued….

Table S1. Crystallographic data for **1-6** continued.

	**1**	**2**	**3**	**4**	**5**	**6**
**Spherical refinement**						
R_1	0.0406	0.0466	0.0355	0.0453	0.0476	0.0494
wR_2	0.1440	0.1363	0.1256	0.1183	0.1319	0.1445
Goodness of fit	1.045	1.181	1.062	1.095	1.138	1.108
$\Delta\rho$(r) [e Å^{-3}] all data	0.505/-0.392	0.643/-0.350	0.773/-0.334	0.646/-0.357	0.745/-0.282	0.675/-0.409
**Multipole refinement**						
$R(F)$	0.0274	0.0385	0.0181	0.0352	0.0382	0.0389
$R(F^2)$	0.0450	0.0302	0.0295	0.0302	0.0385	0.0366
Goodness of fit	1.4425	2.0855	1.6715	1.7228	1.5940	1.5474
$\Delta\rho$(r) [e Å^{-3}]	0.202	0.252	0.182	0.308	0.261	0.389
	-0.311	-0.203	-0.264	-0.276	-0.209	-0.218
CCDC REFCODE[a]	HAYQUT	BAZWUU	HAYQON	HAYTOQ	EMETIA	DAZGUI

[a]Data has been deposited at the Cambridge Crystallographic Data Centre, 12 Union Rd, Cambridge CB2 1EZ (www.ccdc.cam.ac.uk) with submission numbers: 2094439-2094444.

Table S2. Molecular Geometry for Compounds **1-6**, in order of decreasing $Me_2N\cdots C$ separation.

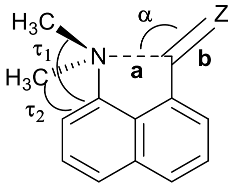

Z = O or CX_2

X = electron withdrawing group

	**a** / Å	**b** / Å	**α** /°	**τ1** /°	**τ2** /°	Naphthalene plane / alkene (or carbonyl) plane /°
**1**	2.6758(4)	1.3509(3)	118.27(1)	-25.40(4)	105.55(3)	53.49(1)
**2**	2.4796(7)	1.2243(4)	113.06(2)	-44.58(3)	85.53(2)	61.67(3)
**3**	2.4163(2)	1.36585(18)	112.43(1)	-49.65(2)	81.61(2)	64.46(1)
**4**	1.6467(5)	1.4703(5)	115.14(3)	-49.27(5)	72.35(5)	65.42(2)
**5**	1.6237(9)	1.4747(8)	115.72(3)	-50.71(5)	70.64(5)	68.63(1)
**6B**	1.6252(7)	1.4687(6)	114.63(3)	-57.16(7)	64.24(6)	72.94(2)
**6A**	1.6070(6)	1.4737(8)	115.00(4)	-44.41(7)	78.50(6)	63.72(2)

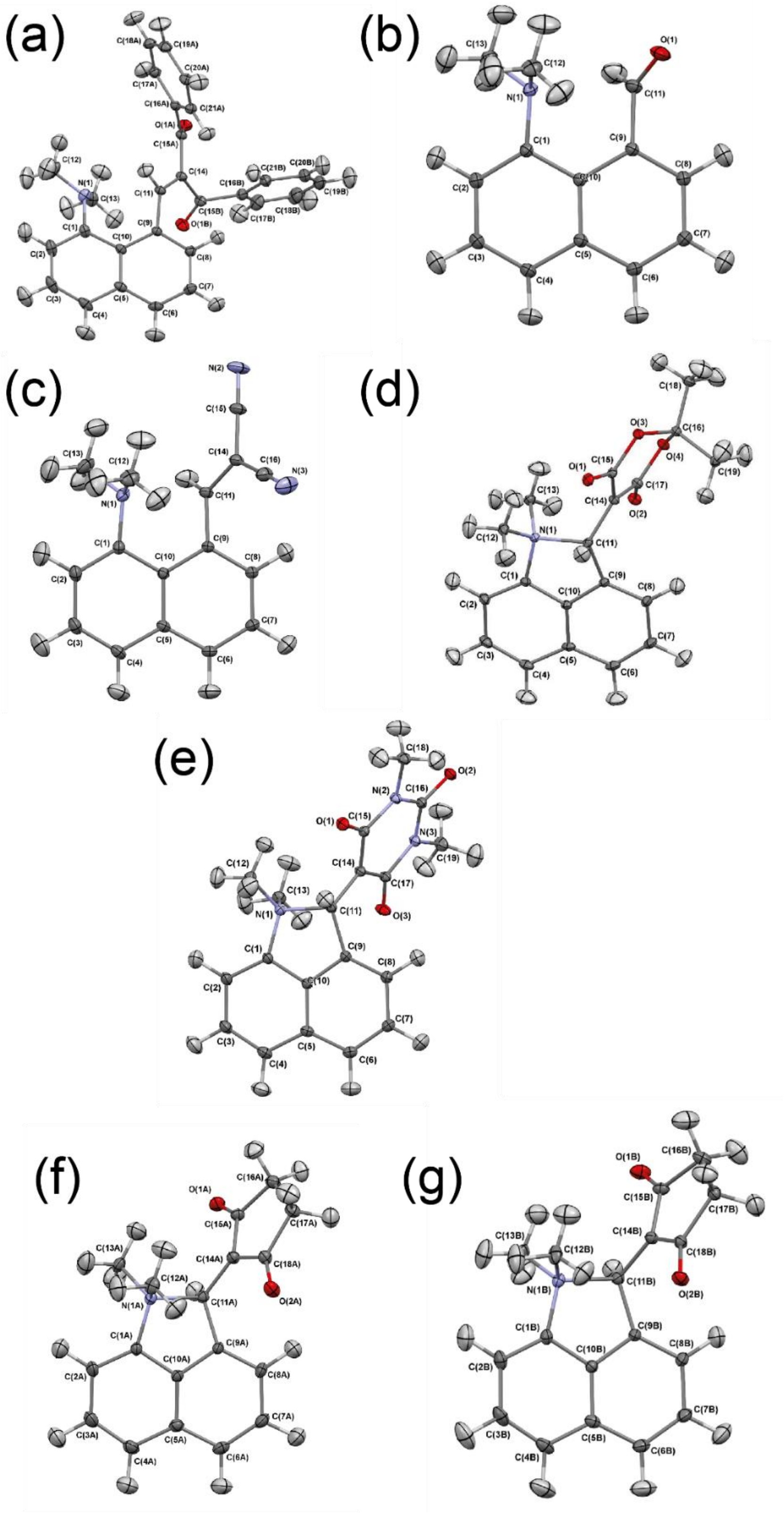

Figure S2: Molecular structures of **1-5**, (a)-(e) and the two independent molecules of **6**, (f) and (g), with anisotropic displacement parameters for non-hydrogen atoms at the 50% level and anisotropic displacement parameters for non-hydrogen atoms were estimated using the SHADE3 server.[S12]

Multipolar refinement.

Modelling the electron density distribution in each crystal structure using the experimental X-ray diffraction data was performed using the multipole refinement model advocated by Hansen and Coppens[S13]. The electron density is described using the formula shown in Equation 1, where $\rho_c(r)$ and $\rho_v(r)$ are the spherical core and valence electron densities, the summation in the third term accounts for the valence electron deformations and the $d_{lm\pm}$ are density-normalised real spherical harmonics expressed in polar coordinates. The isolated atom valence density and the Slater type radial functions R_1 are modified by the scaling factors (κ and κ') to account for the radial expansion or contraction of the valence shell.

$$\rho(\mathrm{r}) = \rho_{\mathrm{c}}(\mathrm{r}) + P_v \kappa^3 \rho_v(\kappa \mathrm{r}) + \sum_{l=0}^{l_{\max}} \kappa'^3 R_1(\kappa' \mathrm{r}) \sum_{m=0}^{+l} P_{lm} d_{lm\pm}(\upsilon, \varphi) \tag{1}$$

The IAM model served as an initial point for the further aspherical atom refinement, using the Hansen-Coppens formalism[S13] as implemented in the XD2016 program.[S14] The multipole refinement was carried out in a stepwise manner. Initially, only the scale factor was refined on all data. Next, accurate positional and displacement parameters for all non-hydrogen atoms were obtained from the high order refinement ($\sin\theta/\lambda > 0.7$ Å^{-1}) whereas positional and isotropic displacement for hydrogen atoms were refined using low-angle data ($\sin\theta/\lambda < 0.7$ Å^{-1}). Due to the unavailability of neutron data all C-H distances were fixed to averaged distances from neutron studies[S15]. The anisotropic displacement parameters for each hydrogen atom in the crystal structure were then determined using the SHADE3-(Simple Hydrogen Anisotropic Displacement Estimator) server.[S12] These were imported into the multipole model and fixed throughout the subsequent refinement steps. Next, multipole populations were introduced, and the complexity of the model was gradually increased. For all carbon atoms, the atomic populations were refined up to octapolar level (l=3), whereas all heteroatoms (N and O) were refined as hexadecapole (l=3) with a single expansion/contraction κ parameter. All hydrogen atoms were represented by the bond directed dipole (l=1). Chemically and symmetry-related atoms were constrained to share the same expansion/contraction (κ/κ') parameters. Throughout multipole refinement the expansion/contraction (κ/κ') parameters of all hydrogen atoms were fixed to default values κ=1.2 and κ' = 1.2. The multipole refinement was performed in blocks until satisfactory convergence was achieved. To reduce the number of parameters several chemical constraints for similar atoms were applied at the initial stages of multipole refinement. These constraints were gradually released, and the final model was chemically unconstrained. The electron neutrality condition was imposed on the molecule for the entire refinement. Final multipole refinement led to a featureless residual density map for all refined crystal structures. The Gaussian distributions of the residual electron densities (see fractal dimension distribution plots[S16] (Fig. S3)) suggest that the remaining residual densities are consistent with noise and that the electron density has been fitted appropriately. The resulting agreement factors for the six data sets are summarised in Table S1. Final structural information containing atomic coordinates, displacement parameters, multipole populations are presented in Tables **S3**-**S14**. Static deformation charge density maps and Laplacian maps are given in Figs **S4**-**S20**, and bond path plots are given in Figs **S21**-**S26**.

Theoretical quantum mechanics studies.

To validate the electronic models of molecules **1-6** calculated from experimental data with XDPROP[S14] and also to obtain electronic properties for two molecules for which high-resolution X-ray data were unavailable, Senkirkine **S1** and Clivorine S**2**, DFT computational studies were performed with Gaussian09 software package,[S17] using B3LYP functionals[S18] and the 6-311++G** basis set. The resulting wave functions for models based on molecular geometries (not optimised) from single crystal diffraction studies were analysed in terms of electronic distribution and properties using AIMAll and AIM2000.[S19] The properties of the topological analysis of electron density distribution at bond critical points (BCPs) are in good agreement with those derived from experimental models for **1**-**6**.

Fractal Distribution Plots and Residual Density Maps:

1, the dibenzoyl derivative.

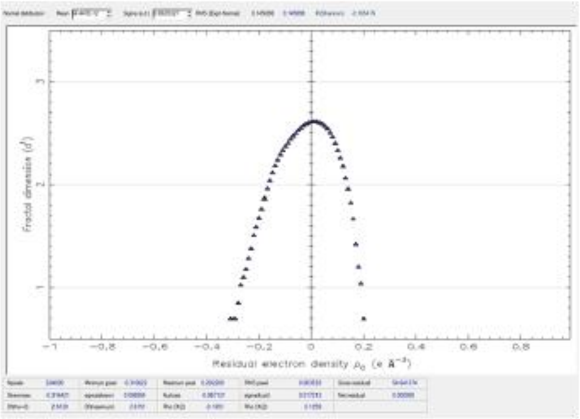

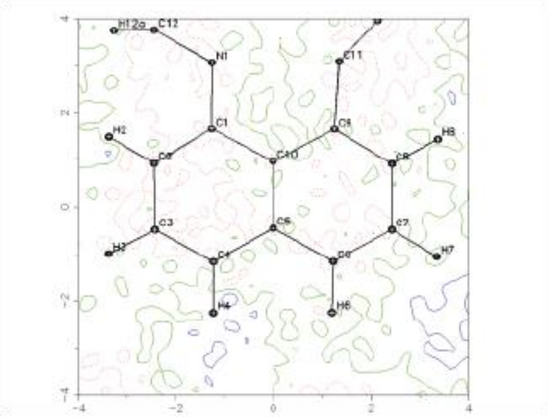

2, the aldehyde derivative.

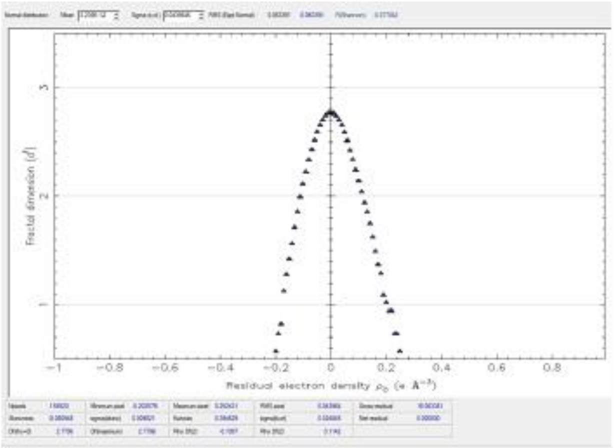

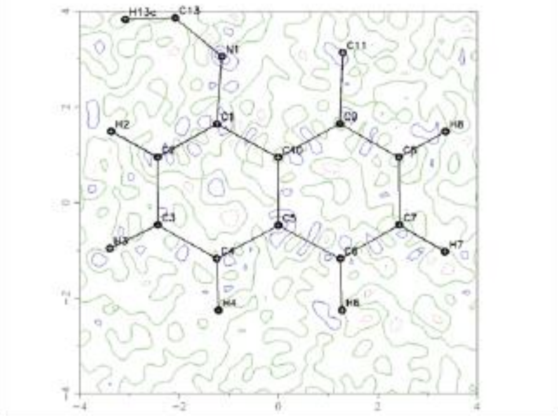

3, the dinitrile derivative.

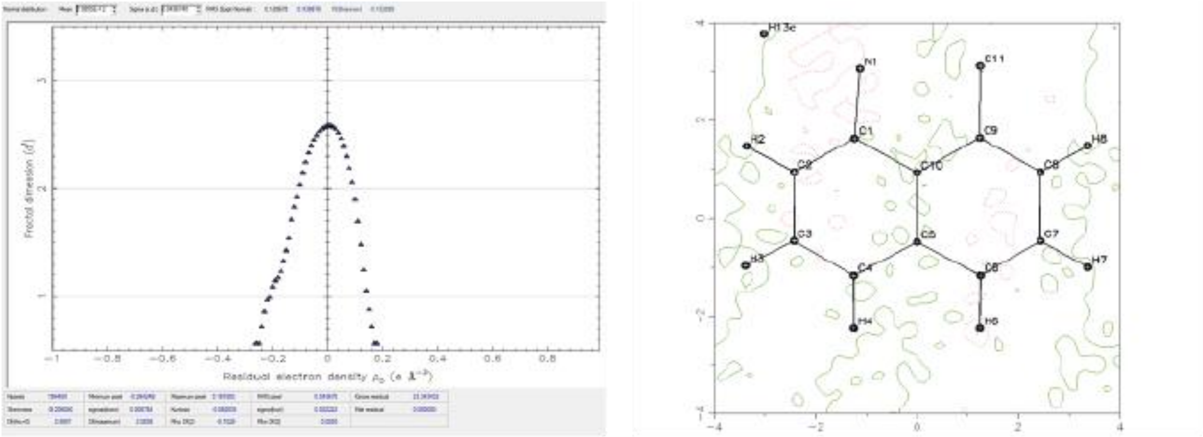

4, the Meldrum's acid acid condensation product.

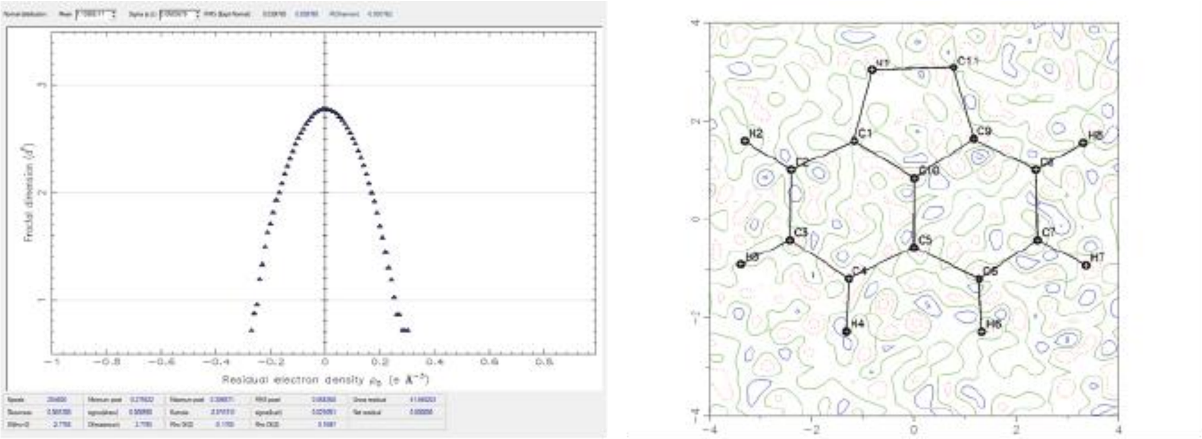

5, the dimethylbarbituric acid condensation product.

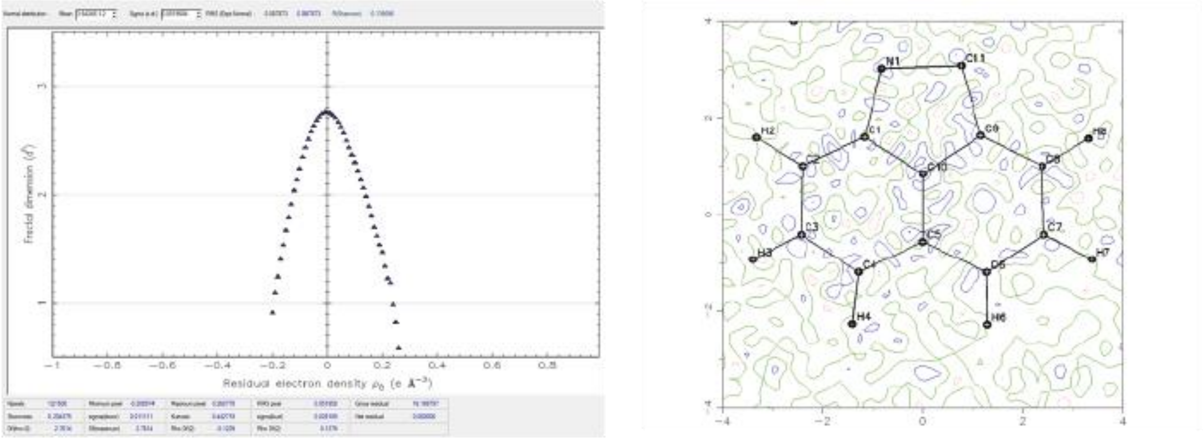

6, the cyclopenta-1,3-dione condensation product.

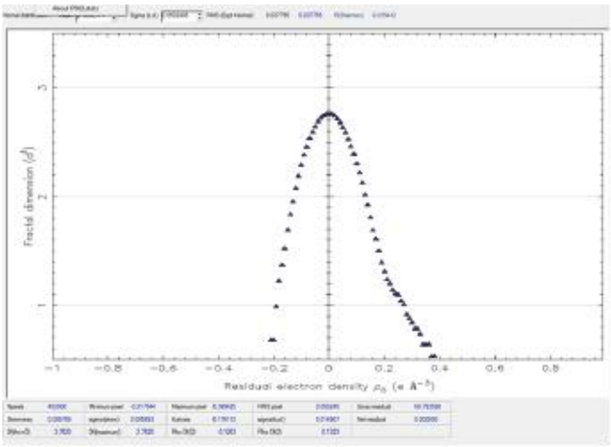

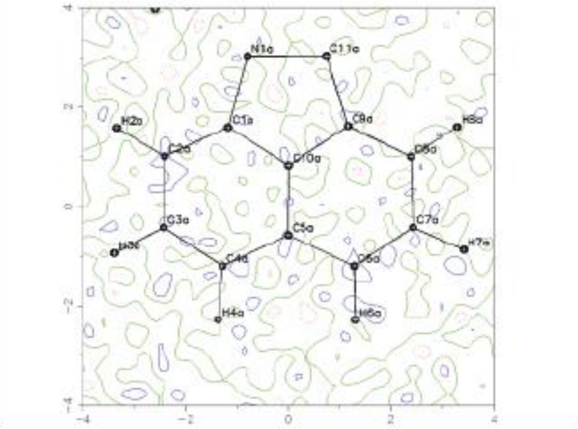

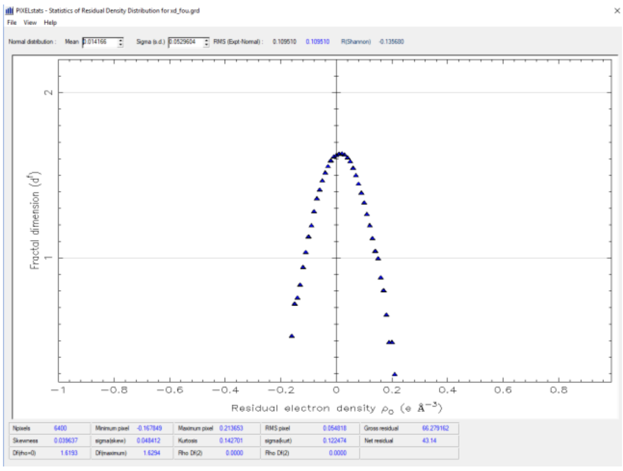

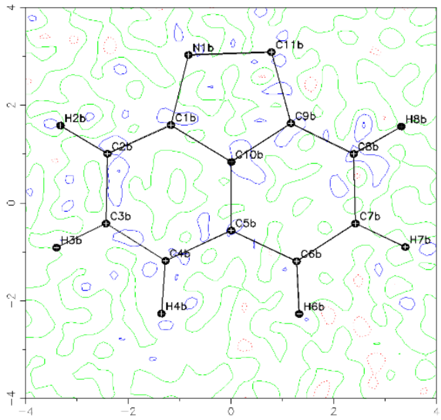

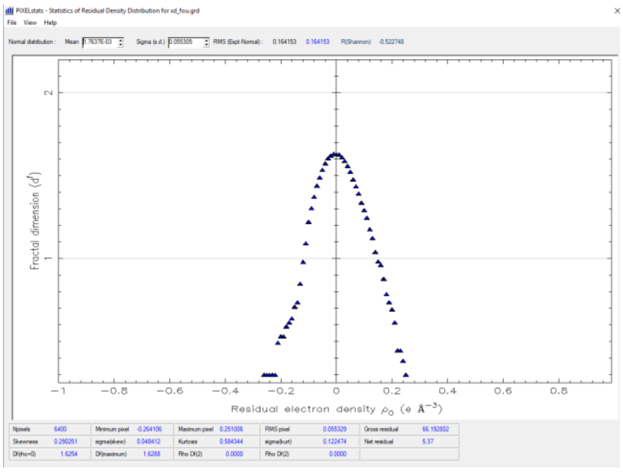

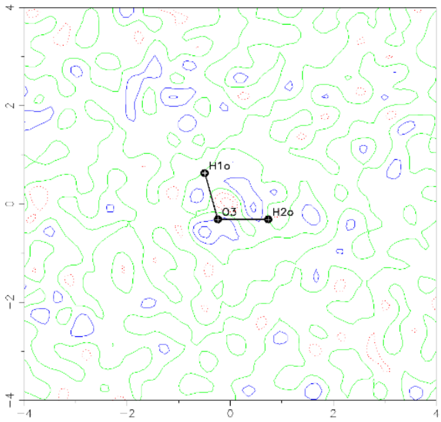

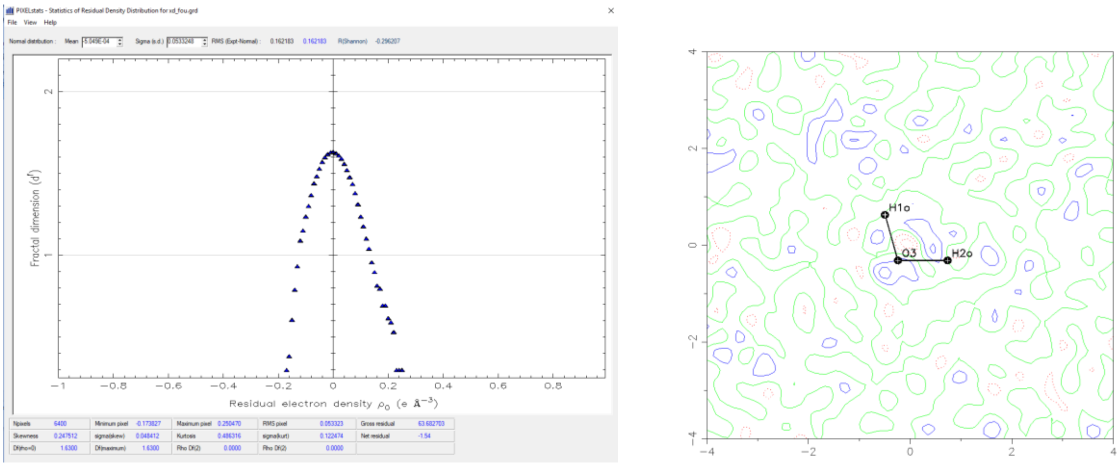

Figure S3: The fractal distribution plots (left) and the residual electron density maps (right) for **1**-**6**.

Static deformation charge density distribution maps and -$\nabla^2\rho(r)$ maps.

For each crystal structure, the first static deformation charge density distribution map and the Laplacian (-$\nabla^2\rho(r)$) map are plotted in the plane of naphthalene ring after multipole refinement. The second set of maps are plotted in the plane of the two *peri* atoms, N1, and C11 and the ring carbon attached to the N atom, C1, to show clearly the situation along the vector between the two *peri* atoms. For the static deformation maps, the positive electron density is shown in blue, negative electron density in red. Zero contours are dashed. Contours are at 0.1 *e* Å^{-3}. For the Laplacian maps, negative values are in red, positive values are in blue, zero contours are dashed, and contours are on a logarithmic scale, *e* Å^{-5}.

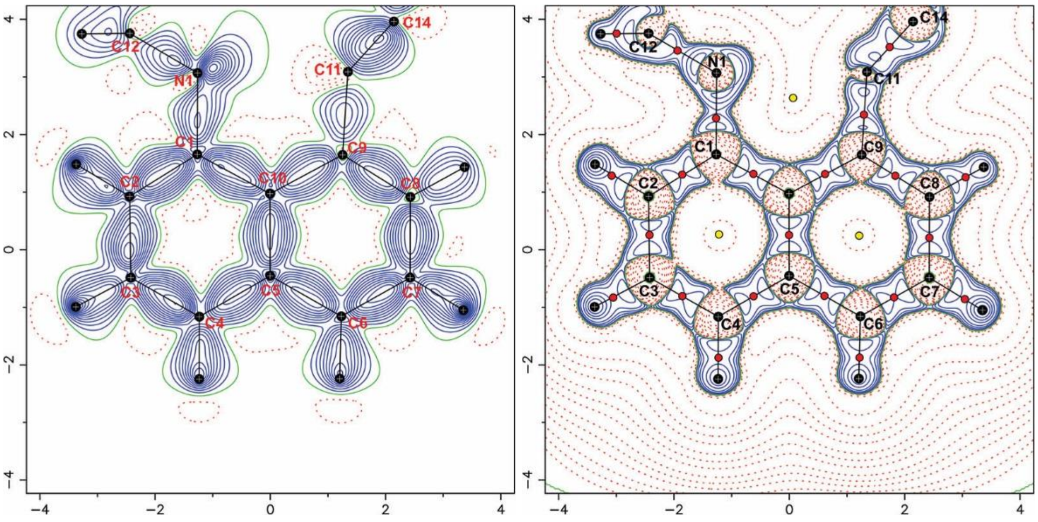

Figure S4: Static deformation charge density distribution map (left) and Laplacian -$\nabla^2\rho(r)$ (right) for **1** (naphthalene plane).

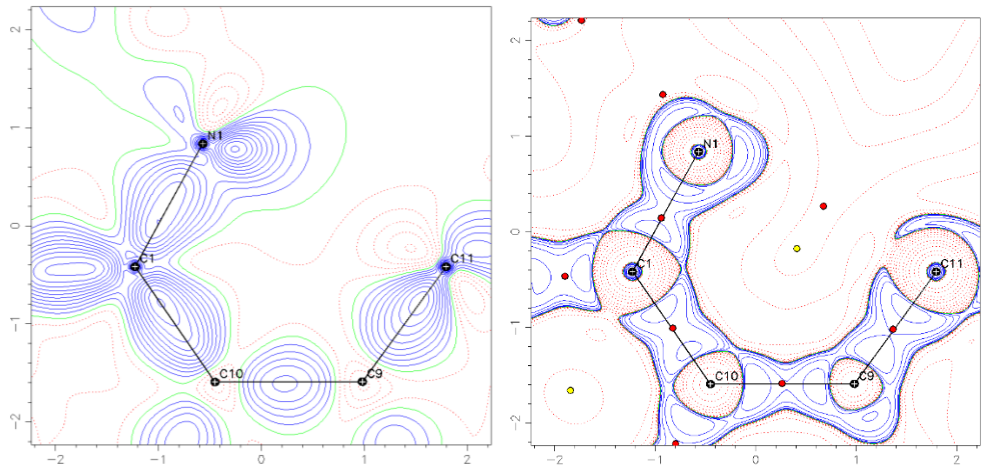

Figure S5: Static deformation charge density distribution map (left) and Laplacian -$\nabla^2\rho$(r) (right) for **1** (C1, N1, C11 plane).

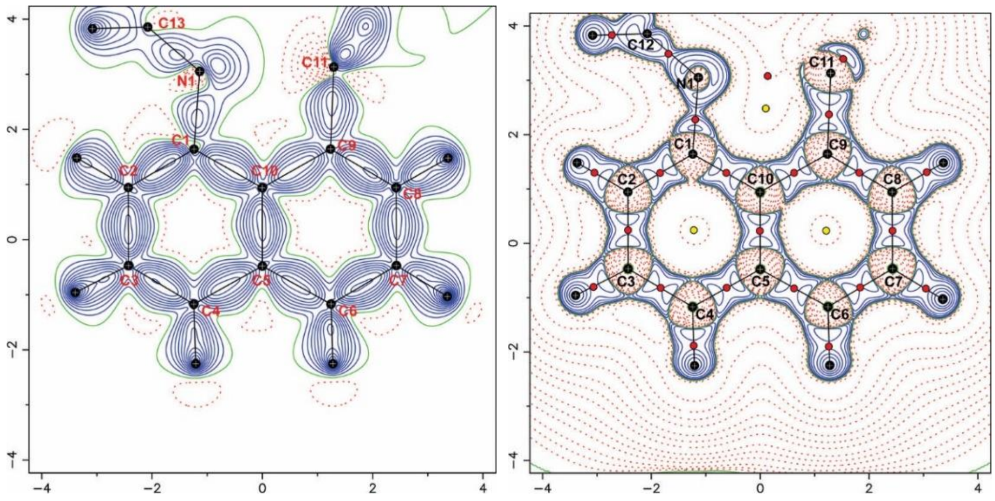

Figure S6: Static deformation charge density distribution map (left) and Laplacian -$\nabla^2\rho$(r) (right) for **2** (naphthalene plane).

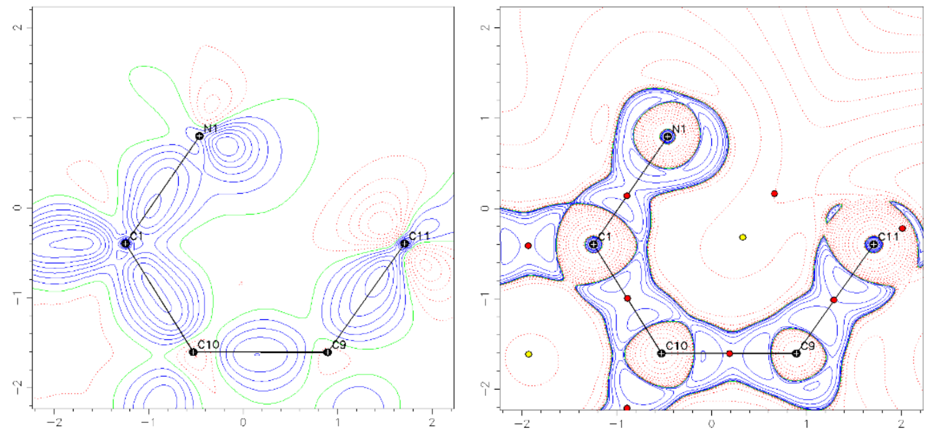

Figure S7: Static deformation charge density distribution map (left) and Laplacian -$\nabla^2\rho$(r) (right) for **2** (C1, N1, C11 plane).

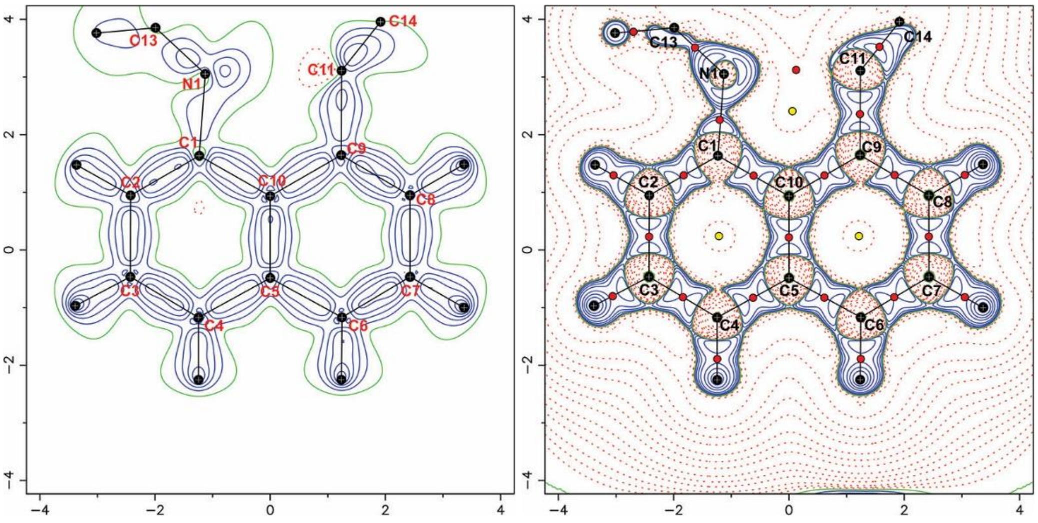

Figure S8: Static deformation charge density distribution map (left) and Laplacian $-\nabla^2\rho(r)$ (right) for **3** (naphthalene plane).

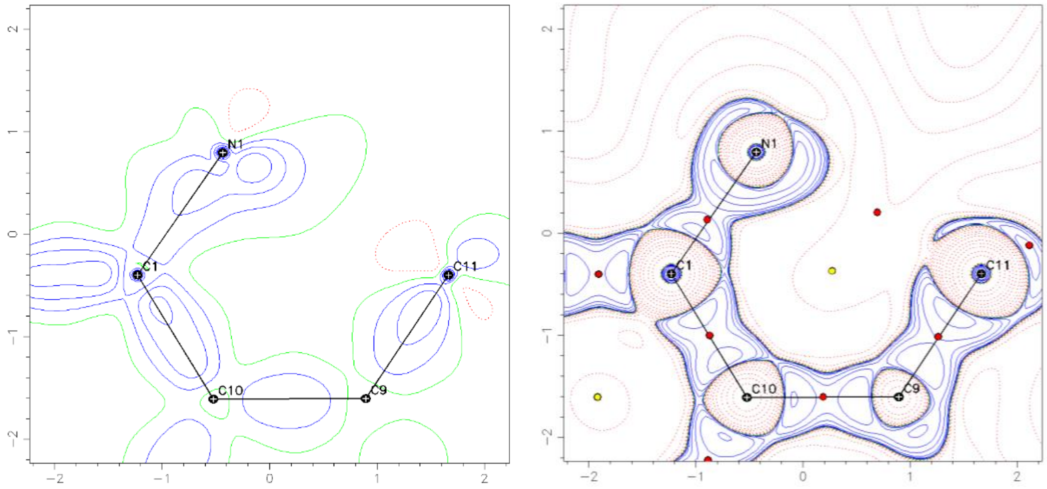

Figure S9: Static deformation charge density distribution map (left) and Laplacian $-\nabla^2\rho(r)$ (right) of **3** (C1, N1, C11 plane).

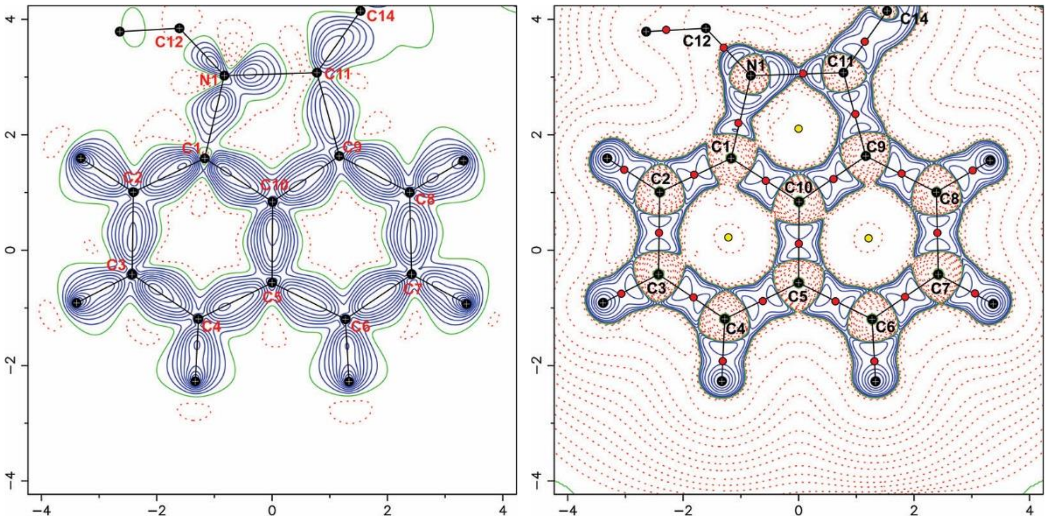

Figure S10: (Static deformation charge density distribution map (left) and Laplacian -$\nabla^2\rho$(r) (right) of **4** (naphthalene plane).

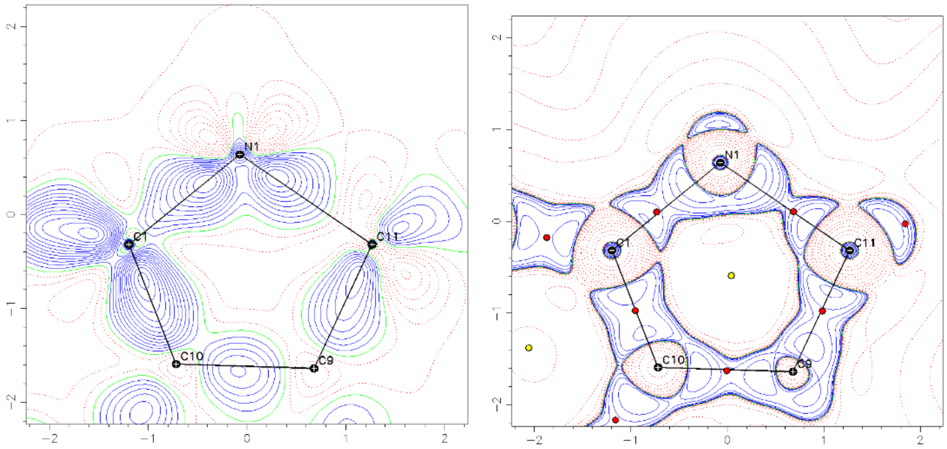

Figure S11: Static deformation charge density distribution map (left) and Laplacian -$\nabla^2\rho$(r) (right) of **4** (C1, N1, C11 plane).

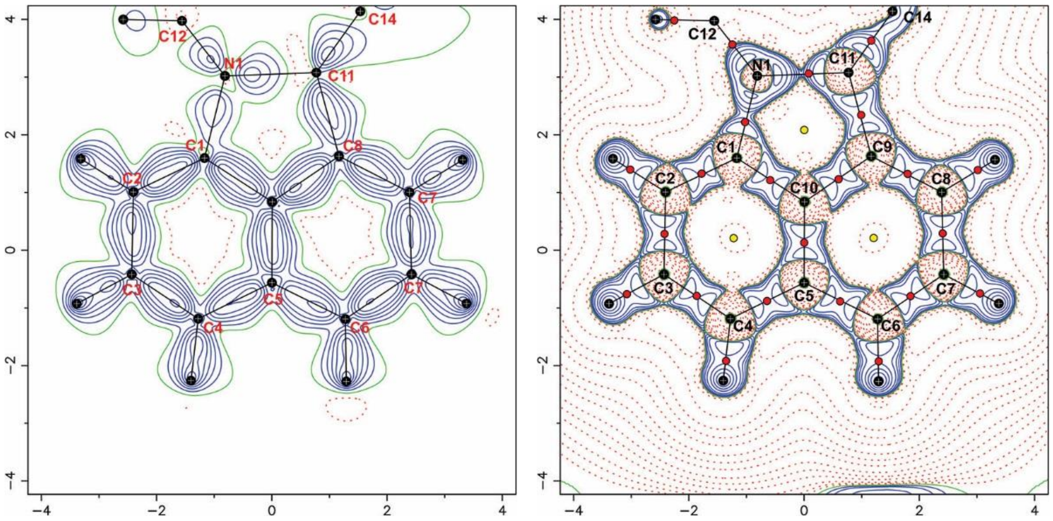

Figure S12: Static deformation charge density distribution map (left) and Laplacian -$\nabla^2\rho$(r) (right) of **5** (naphthalene plane)

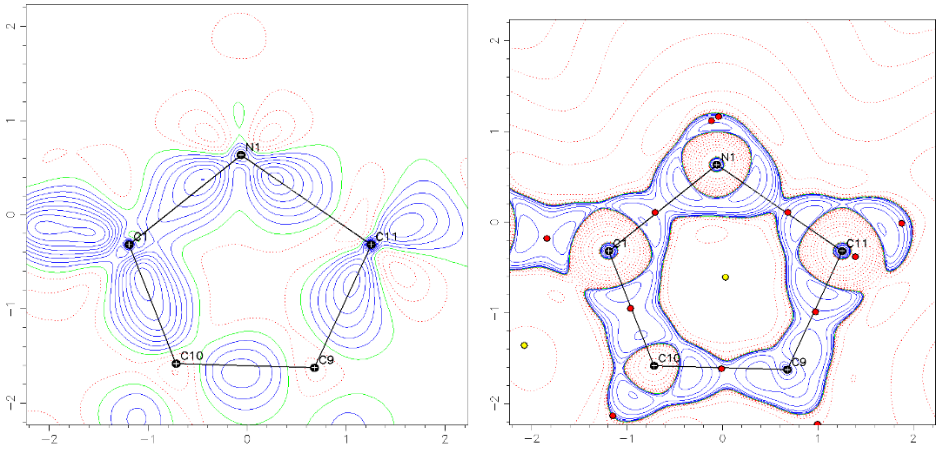

Figure S13: Static deformation charge density distribution map (left) and Laplacian -$\nabla^2\rho$(r) (right) of **5** (C1, N1, C11 plane).

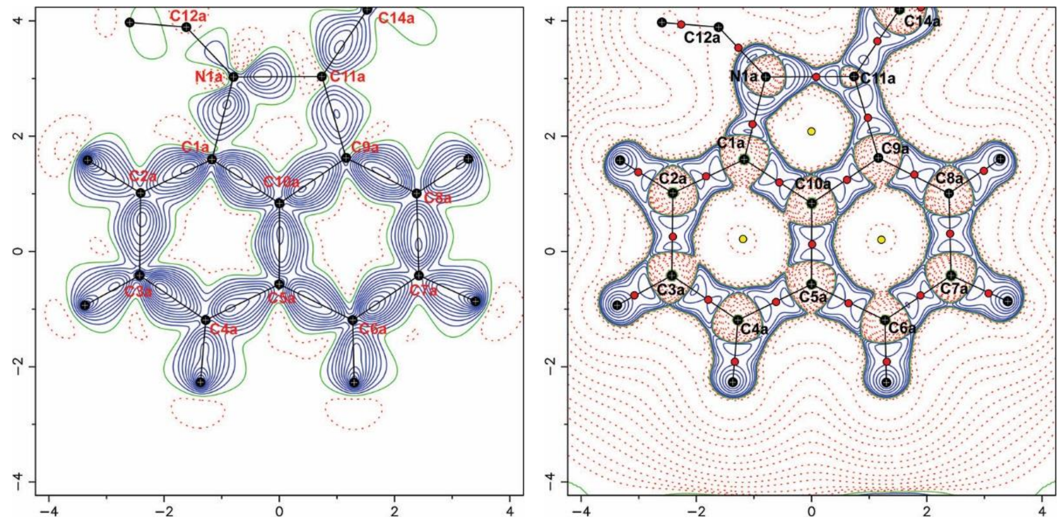

Figure S14: Static deformation charge density distribution map (left) and Laplacian -$\nabla^2\rho$(r) (right) for molecule **A** of **6** (naphthalene plane).

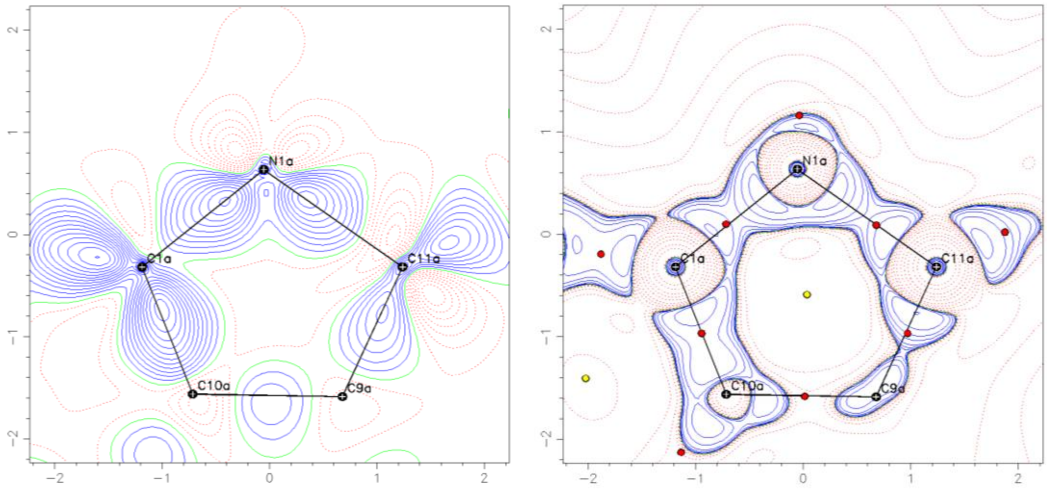

Figure S15: Static deformation charge density distribution map (left) and Laplacian -$\nabla^2\rho$(r) (right) for molecule **A** of **6** (C1, N1, C11 plane).

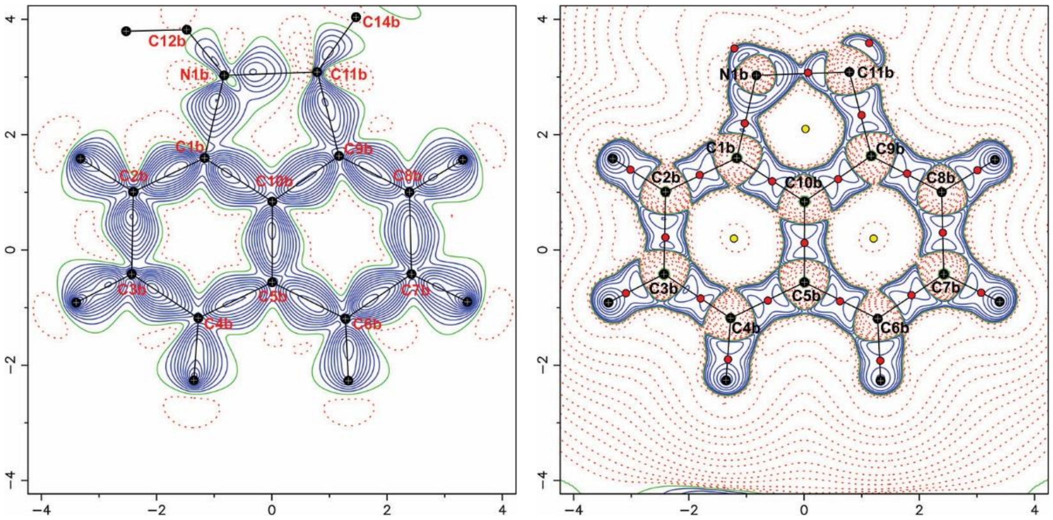

Figure S16: Static deformation charge density distribution map (left) and Laplacian $-\nabla^2\rho(r)$ (right) for molecule **B** of **6** (naphthalene plane).

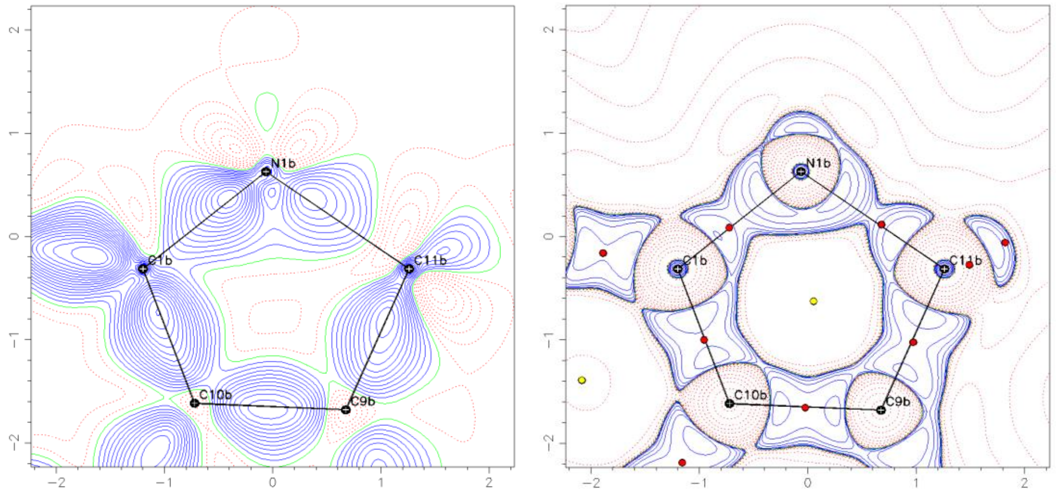

Figure S17: Static deformation charge density distribution map (left) and Laplacian $-\nabla^2\rho(r)$ (right) for Molecule B **6** (C1, N1, C11 plane).

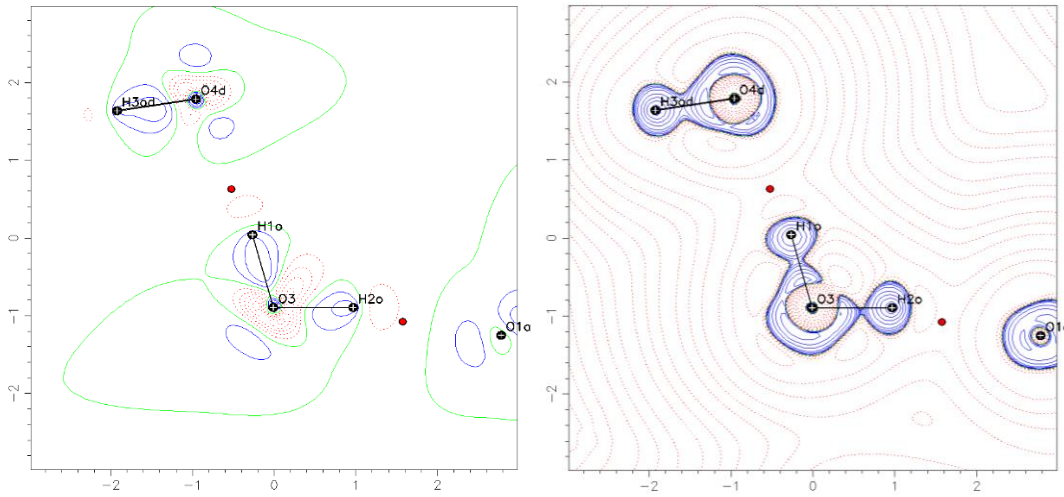

Figure S18: Static deformation charge density distribution map (left) and Laplacian $-\nabla^2\rho(r)$ (right) for water molecule O3 in **6.H_2O** (H2o H1o O4 plane).

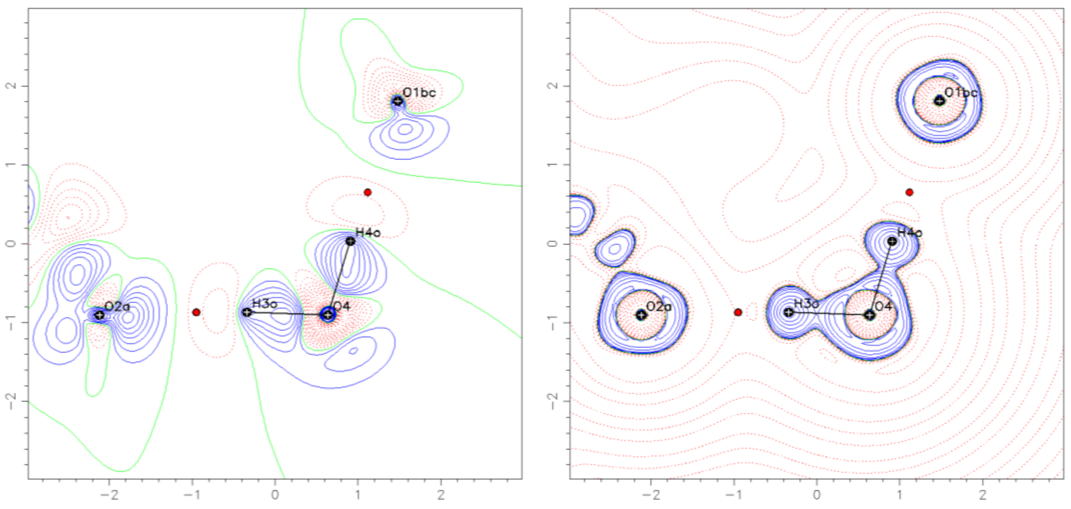

Figure S19: Static deformation charge density distribution map (left) and Laplacian $-\nabla^2\rho(\mathrm{r})$ (right) for water molecule O4 in **6.H_2O** (O2b O4 O1b plane).

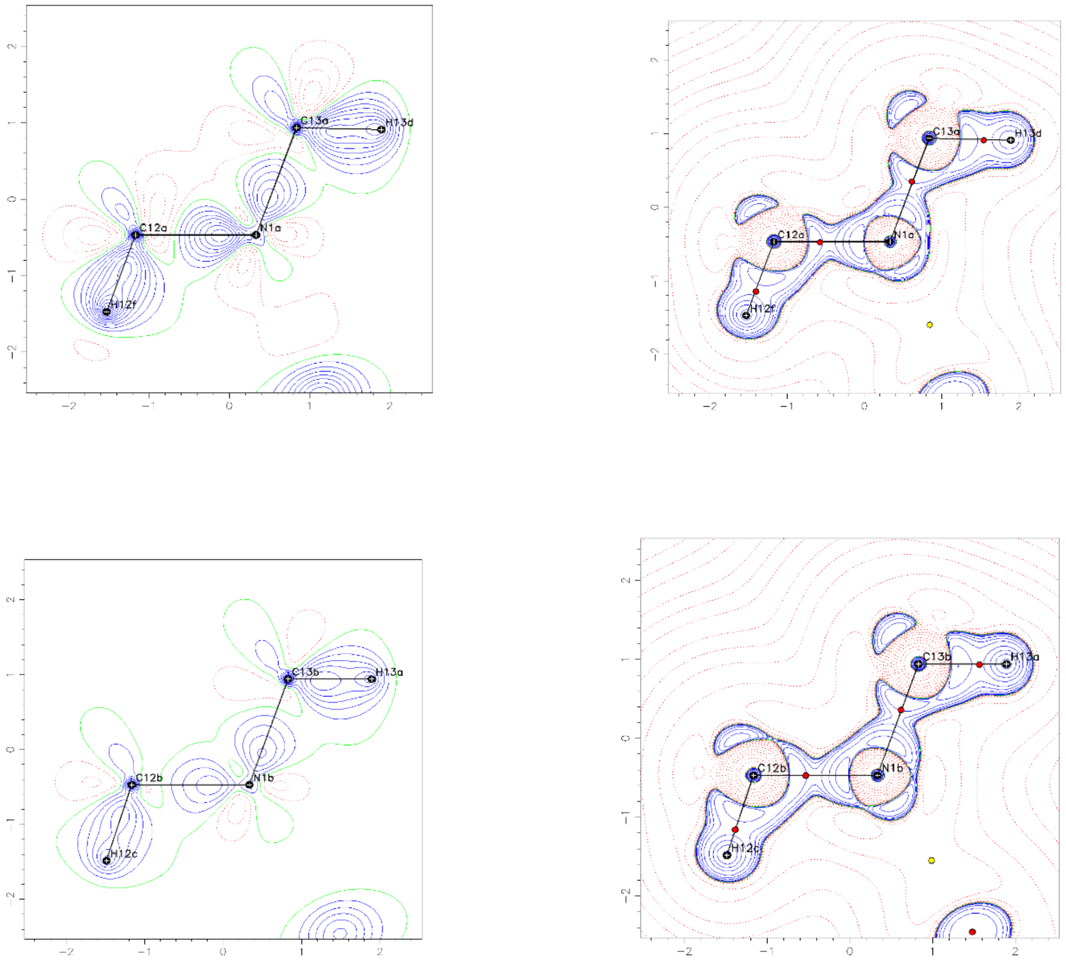

Figure S20. Static deformation density (left) and Laplacian (right) for **6**: for molecule A in C12a N1a C13a plane (top) and molecule B in C12b N1b C13b plane (bottom) showing the concentration of deformation density nearer to the N atom in the two N-CH_3 bonds, and the displacement of the (3,-1) critical point nearer to the C atom. Similar effects are seen in the N-C bond between the *peri* groups.

Bond path plots.

The nuclear positions are displayed in blue, bond paths in yellow, BCPs in red, ring critical points green yellow and cage critical points in green copper.

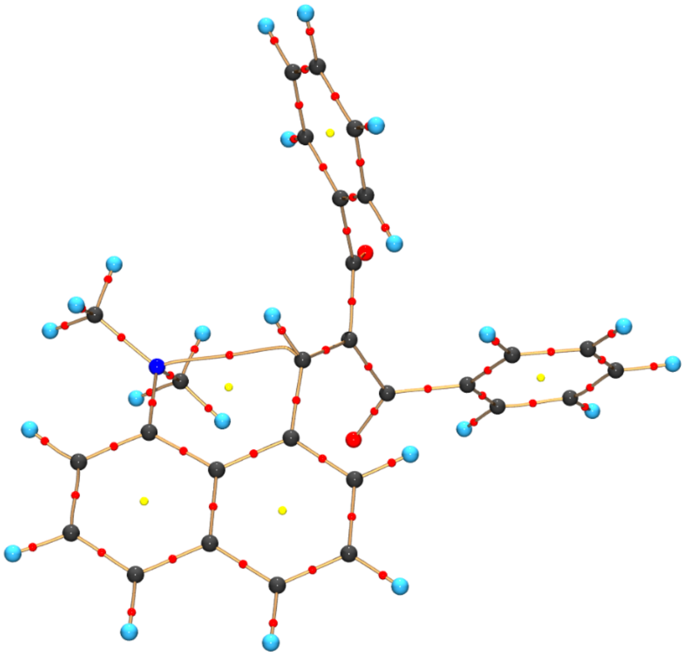

Figure S21: Bond path plot of structure **1** displaying the nuclear positions of the atoms in the structure, the bond paths (paths of maximum electron density linking these nuclear positions), and the positions of the bond critical points (position along the bond path where the electron density is at a minimum).

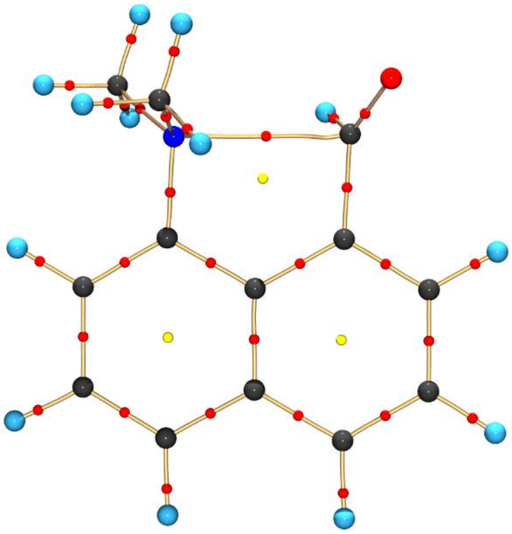

Figure S22: Bond path plot of structure **2** displaying the nuclear positions of the atoms in the structure, the bond paths (paths of maximum electron density linking these nuclear positions), and the positions of the bond critical points (position along the bond path where the electron density is at a minimum).

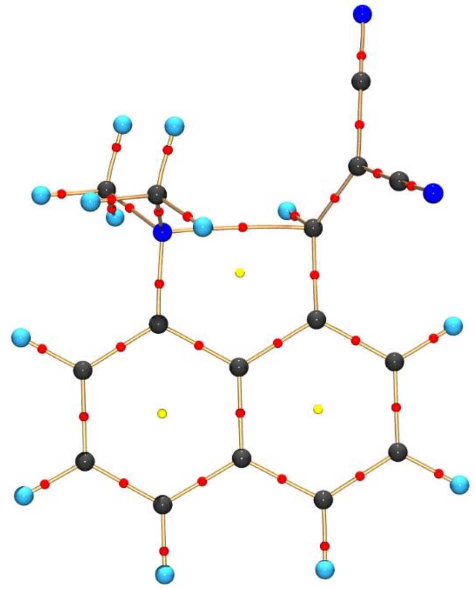

Figure S23: Bond path plot of structure **3** displaying the nuclear positions of the atoms in the structure, the bond paths (paths of maximum electron density linking these nuclear positions), and the positions of the bond critical points (position along the bond path where the electron density is at a minimum).

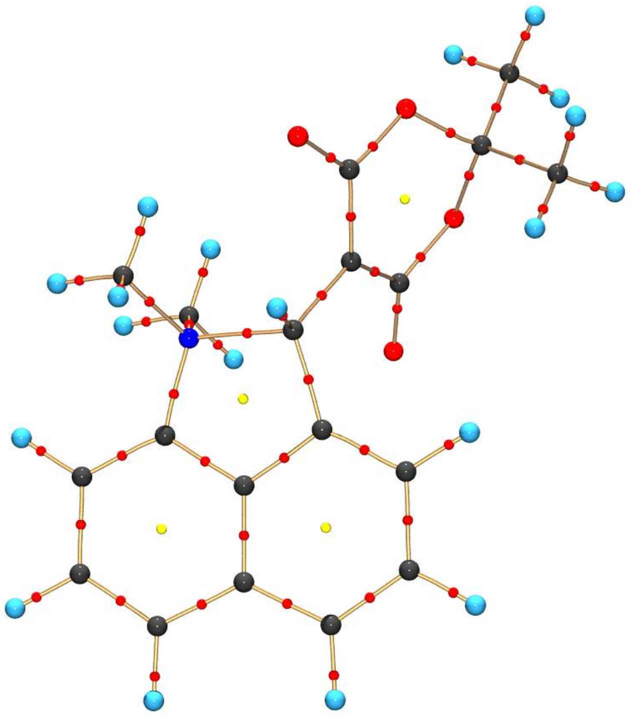

Figure S24: Bond path plot of structure **4** displaying the nuclear positions of the atoms in the structure, the bond paths (paths of maximum electron density linking these nuclear positions), and the positions of the bond critical points (position along the bond path where the electron density is at a minimum).

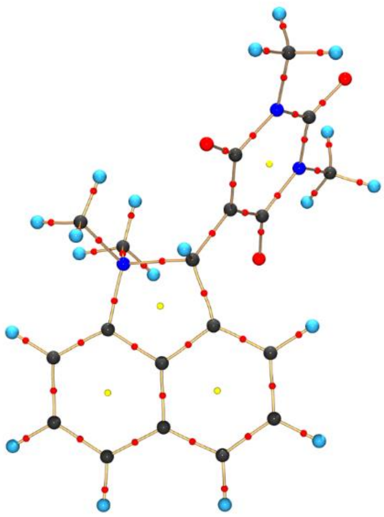

Figure S25: Bond path plot of structure **5** displaying the nuclear positions of the atoms in the structure, the bond paths (paths of maximum electron density linking these nuclear positions), and the positions of the bond critical points (position along the bond path where the electron density is at a minimum).

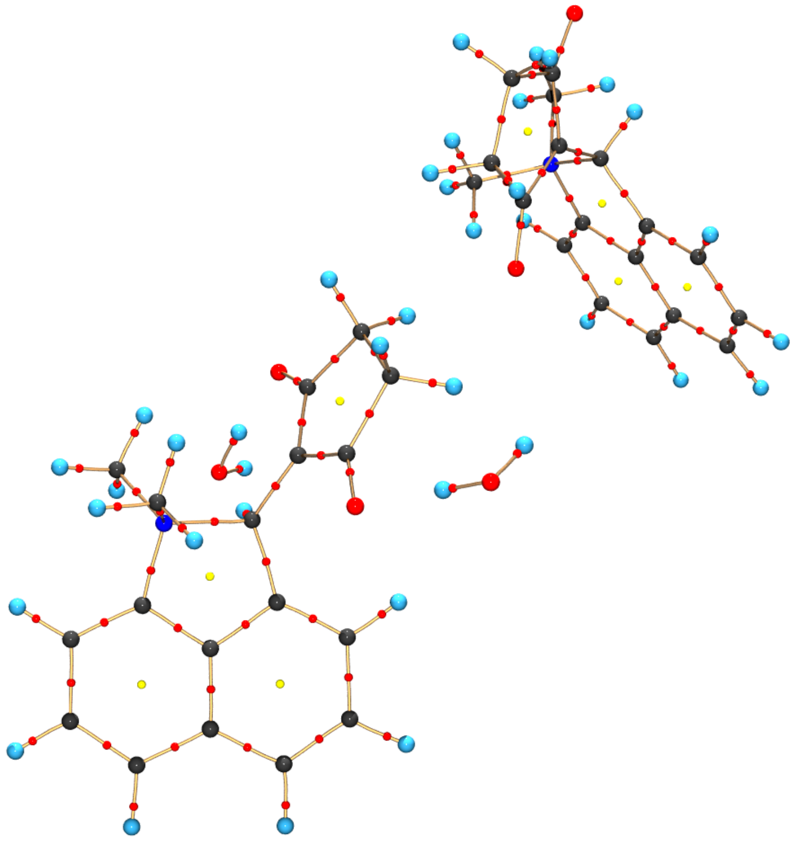

Figure S26: Bond path plot of structure **6** displaying the nuclear positions of the atoms in the structure, the bond paths (paths of maximum electron density linking these nuclear positions), and the positions of the bond critical points (position along the bond path where the electron density is at a minimum).

Electronic properties of the electron density distribution at the BCPs for the covalent bonds.

Table S3: Properties of the electron density distribution at the BCPs for the covalent bonds in **1**.

Bond	ρ(r) (e A^{-3})	$\nabla^2\rho$(r) (e A^{-5})	Rij (Å)	d_1 (Å)	d_2 (Å)	λ_1	λ_2	λ_3	ε
O(1A) -C(15A)	2.892	-34.528	1.2295	0.7709	0.4586	-27.52	-24.84	17.83	0.11
O(1B) -C(15B)	2.921	-36.670	1.2201	0.7542	0.4659	-28.32	-24.95	16.60	0.14
N(1) -C(1)	2.034	-14.164	1.4171	0.7856	0.6315	-16.40	-14.51	16.75	0.13
N(1) -C(12)	1.813	-11.067	1.4561	0.8309	0.6252	-13.29	-11.68	13.91	0.14
N(1) -C(13)	1.781	-11.235	1.4591	0.8271	0.6321	-12.83	-12.06	13.66	0.06
N(1) -C(11)	0.130	1.688	2.6953	1.3734	1.3219	-0.36	-0.20	2.24	0.84
C(1) -C(2)	2.178	-18.814	1.3832	0.6856	0.6975	-17.42	-13.62	12.22	0.28
C(1) -C(10)	1.979	-14.999	1.4367	0.7286	0.7081	-15.40	-12.51	12.91	0.23
C(2) -C(3)	2.025	-16.702	1.4114	0.6704	0.741	-15.35	-13.06	11.71	0.18
C(2) -H(2)	1.794	-15.463	1.0840	0.7323	0.3517	-17.58	-16.97	19.09	0.04
C(3) -C(4)	2.249	-21.238	1.3713	0.6516	0.7197	-18.20	-14.36	11.32	0.27
C(3) -H(3)	1.794	-15.808	1.0832	0.7202	0.3629	-17.27	-16.53	18.00	0.04
C(4) -C(5)	2.024	-16.561	1.4197	0.7066	0.7132	-15.55	-13.26	12.24	0.17
C(4) -H(4)	1.798	-15.651	1.0831	0.7141	0.369	-17.41	-16.24	18.00	0.07
C(5) -C(6)	2.033	-16.255	1.4216	0.7043	0.7173	-15.48	-13.29	12.52	0.16
C(5) -C(10)	1.985	-15.043	1.4265	0.7081	0.7183	-14.94	-12.78	12.67	0.17
C(6) -C(7)	2.211	-20.302	1.3731	0.6708	0.7024	-17.55	-14.17	11.42	0.24
C(6) -H(6)	1.819	-16.105	1.0830	0.7180	0.3651	-17.57	-16.75	18.22	0.05
C(7) -C(8)	2.072	-17.682	1.4080	0.6970	0.7111	-16.00	-13.52	11.83	0.18
C(7) -H(7)	1.807	-15.907	1.0831	0.7189	0.3641	-17.53	-16.69	18.31	0.05
C(8) -C(9)	2.165	-18.562	1.3849	0.6968	0.6881	-17.07	-13.71	12.22	0.24
C(8) -H(8)	1.789	-15.047	1.083	0.7198	0.3632	-17.23	-16.29	18.48	0.06
C(9) -C(10)	1.938	-14.592	1.4351	0.7200	0.7151	-14.70	-12.49	12.59	0.18
C(9) -C(11)	1.810	-13.152	1.4749	0.7101	0.7648	-13.2	-12.31	12.36	0.07
C(11) -C(14)	2.278	-19.872	1.3509	0.6672	0.6837	-18.08	-13.72	11.93	0.32
C(11) -H(11)	1.871	-18.369	1.0771	0.7301	0.3471	-18.95	-18.2	18.78	0.04
C(12) -H(12A)	1.620	-10.013	1.0661	0.7178	0.3484	-14.88	-14.16	19.03	0.05
C(12) -H(12B)	1.707	-12.087	1.066	0.7447	0.3213	-17.01	-15.84	20.77	0.07
C(12) -H(12C)	1.764	-15.009	1.0663	0.7185	0.3478	-16.95	-16.29	18.23	0.04
C(13) -H(13A)	1.886	-16.876	1.0661	0.7164	0.3496	-18.29	-17.86	19.28	0.02
C(13) -H(13B)	1.771	-14.652	1.0663	0.7068	0.3594	-16.62	-16.11	18.08	0.03
C(13) -H(13C)	1.730	-12.552	1.0662	0.7246	0.3416	-16.65	-15.94	20.04	0.04
C(14) -C(15A)	1.817	-12.673	1.4832	0.7284	0.7548	-13.67	-11.77	12.77	0.16
C(14) -C(15B)	1.746	-11.859	1.5096	0.7484	0.7611	-12.81	-11.72	12.67	0.09
C(15A) -C(16A)	1.756	-11.854	1.4923	0.7564	0.736	-13.01	-11.68	12.83	0.11
C(15B) -C(16B)	1.801	-12.107	1.4921	0.7562	0.7359	-13.51	-11.81	13.21	0.14
C(16A) -C(17A)	2.091	-17.226	1.4005	0.7028	0.6977	-16.11	-13.37	12.25	0.20
C(16A) -C(21A)	2.131	-18.07	1.3981	0.6900	0.7081	-16.64	-13.81	12.37	0.21
C(16B) -C(17B)	2.127	-18.275	1.3976	0.6780	0.7195	-16.59	-13.86	12.17	0.20
C(16B) -C(21B)	2.114	-18.053	1.3951	0.6582	0.7369	-16.46	-13.6	12.01	0.21
C(17A) -C(18A)	2.114	-18.762	1.3898	0.6921	0.6976	-16.71	-13.28	11.23	0.26
C(17A) -H(17A)	1.824	-16.113	1.0831	0.7219	0.3612	-17.68	-16.89	18.46	0.05
C(17B) -C(18B)	2.161	-19.527	1.3914	0.6743	0.7171	-16.84	-14.09	11.4	0.19
C(17B) -H(17B)	1.808	-16.986	1.0832	0.7523	0.3309	-18.71	-17.4	19.12	0.07
C(18A) -C(19A)	2.147	-18.990	1.3972	0.6997	0.6975	-16.81	-13.85	11.67	0.21
C(18A) -H(18A)	1.796	-15.673	1.0831	0.7349	0.3482	-17.91	-16.8	19.04	0.07
C(18B) -C(19B)	2.127	-20.395	1.3942	0.6097	0.7845	-16.34	-14.08	10.03	0.16
C(18B) -H(18B)	1.712	-13.815	1.0830	0.7296	0.3534	-16.53	-15.55	18.27	0.06
C(19A) -C(20A)	2.136	-18.456	1.3945	0.6723	0.7222	-16.52	-13.51	11.57	0.22
C(19A) -H(19A)	1.797	-15.326	1.0833	0.7212	0.3621	-17.17	-16.61	18.45	0.03
C(19B) -C(20B)	2.152	-19.484	1.3912	0.6643	0.7269	-17.04	-13.73	11.29	0.24
C(19B) -H(19B)	1.809	-17.081	1.0831	0.7201	0.363	-17.85	-16.64	17.41	0.07

C(20A) -C(21A)	2.107	-18.679	1.3961	0.6979	0.6982	-16.35	-13.64	11.32	0.20
C(20A) -H(20A)	1.836	-16.642	1.0830	0.7224	0.3606	-17.93	-16.99	18.28	0.06
C(20B) -C(21B)	2.141	-18.905	1.3921	0.6802	0.7119	-16.39	-13.94	11.42	0.18
C(20B) -H(20B)	1.730	-14.765	1.0842	0.7018	0.3824	-16.20	-15.28	16.71	0.06
C(21A) -H(21A)	1.817	-15.772	1.0831	0.7297	0.3534	-17.83	-16.94	19.00	0.05
C(21B) -H(21B)	1.801	-16.510	1.0831	0.7329	0.3501	-17.99	-16.88	18.37	0.07

Table S4: Properties of the electron density distribution at the BCPs for the covalent bonds in **2**.

Bond	ρ(r) (e A^{-3})	$\nabla^2\rho$(r) (e A^{-5})	Rij (Å)	d_1 (Å)	d_2 (Å)	λ_1	λ_2	λ_3	ε
O(1) -C(11)	2.857	-39.018	1.2245	0.7762	0.4483	-29.23	-26.37	16.58	0.11
N(1) -C(1)	1.976	-12.397	1.4279	0.7835	0.6444	-15.24	-14.34	17.18	0.06
N(1) -C(11)	0.192	1.971	2.4805	1.2922	1.1883	-0.59	-0.45	3.01	0.31
N(1) -C(12)	1.737	-8.635	1.4609	0.8104	0.6505	-12.28	-11.58	15.22	0.06
N(1) -C(13)	1.742	-8.954	1.4612	0.8201	0.6411	-12.76	-11.21	15.0	0.14
C(1) -C(2)	2.162	-18.495	1.3802	0.6937	0.6865	-17.08	-13.57	12.16	0.26
C(1) -C(10)	2.014	-15.308	1.4251	0.7083	0.7168	-15.15	-12.78	12.62	0.19
C(2) -C(3)	2.067	-16.830	1.4179	0.7055	0.7124	-15.84	-13.30	12.30	0.19
C(2) -H(2)	1.825	-17.133	1.0831	0.7127	0.3703	-17.63	-16.60	17.10	0.06
C(3) -C(4)	2.142	-18.793	1.3776	0.6928	0.6847	-16.70	-13.55	11.45	0.23
C(3) -H(3)	1.786	-15.189	1.0833	0.7183	0.3650	-17.27	-16.37	18.45	0.06
C(4) -H(4)	1.822	-16.243	1.0830	0.7132	0.3699	-17.52	-16.59	17.87	0.06
C(4) -C(5)	1.996	-15.753	1.4221	0.7193	0.7029	-15.08	-12.71	12.04	0.19
C(5) -C(6)	1.987	-15.736	1.4230	0.7140	0.7090	-14.93	-12.76	11.95	0.17
C(5) -C(10)	1.986	-15.389	1.4223	0.7088	0.7135	-15.01	-12.36	11.98	0.21
C(6) -C(7)	2.157	-19.255	1.3766	0.6915	0.6850	-16.96	-13.84	11.54	0.23
C(6) -H(6)	1.828	-16.798	1.0831	0.7308	0.3523	-18.01	-17.39	18.60	0.04
C(7) -C(8)	2.000	-15.677	1.4156	0.7015	0.7141	-15.13	-12.57	12.03	0.20
C(7) -H(7)	1.804	-15.053	1.0831	0.7126	0.3705	-17.13	-16.37	18.45	0.05
C(8) -C(9)	2.143	-17.781	1.3804	0.7007	0.6797	-16.39	-13.47	12.08	0.22
C(8) -H(8)	1.806	-15.354	1.0830	0.7324	0.3506	-17.70	-17.11	19.45	0.03
C(9) -C(10)	1.934	-14.132	1.4250	0.7061	0.7189	-14.38	-12.04	12.29	0.19
C(9) -C(11)	1.765	-11.666	1.4964	0.7306	0.7658	-12.77	-11.47	12.57	0.11
C(11) -H(11)	1.810	-14.810	1.0775	0.7524	0.3251	-18.46	-17.83	21.48	0.04
C(12) -H(12A)	1.808	-15.764	1.0661	0.7119	0.3542	-17.52	-16.89	18.65	0.04
C(12) -H(12B)	1.815	-15.784	1.0662	0.6839	0.3823	-16.67	-16.04	16.93	0.04
C(12) -H(12C)	1.816	-15.627	1.0664	0.7086	0.3578	-17.32	-16.67	18.36	0.04
C(13) -H(13A)	1.713	-13.442	1.0664	0.6884	0.3780	-15.81	-14.68	17.05	0.08
C(13) -H(13B)	1.798	-14.098	1.0662	0.6950	0.3712	-17.17	-15.49	18.57	0.11
C(13) -H(13C)	1.755	-11.902	1.0663	0.6941	0.3723	-16.15	-14.73	18.98	0.10

Table S5: Properties of the electron density distribution at the BCPs for the covalent bonds in **3**.

Bond	ρ(r) (e A^{-3})	$\nabla^2\rho$(r) (e A^{-5})	Rij (Å)	d_1 (Å)	d_2 (Å)	λ_1	λ_2	λ_3	ε
N(1) -C(1)	1.910	-10.797	1.4323	0.8047	0.6276	-14.57	-13.90	17.68	0.05
N(1) -C(11)	0.213	2.199	2.4180	1.2747	1.1434	-0.69	-0.63	3.52	0.09
N(1) -C(12)	1.792	-10.940	1.4586	0.8272	0.6314	-13.26	-12.00	14.32	0.11
N(1) -C(13)	1.766	-10.385	1.4596	0.8353	0.6243	-13.02	-11.38	14.01	0.14
N(2) -C(15)	3.401	-30.919	1.1578	0.7166	0.4413	-27.44	-27.14	23.65	0.01
N(3) -C(16)	3.400	-29.558	1.1596	0.7218	0.4378	-27.58	-27.09	25.12	0.02
C(1) -C(2)	2.199	-18.426	1.3760	0.6846	0.6913	-18.01	-14.16	13.75	0.27
C(1) -C(10)	2.022	-15.137	1.4205	0.7103	0.7103	-16.14	-13.19	14.19	0.22
C(2) -C(3)	2.012	-15.161	1.4174	0.7194	0.6980	-15.57	-13.08	13.48	0.19
C(2) -H(2)	1.877	-16.614	1.0830	0.7128	0.3702	-18.33	-17.40	19.12	0.05

C(3) -C(4)	2.205	-18.945	1.3776	0.6872	0.6905	-17.77	-14.42	13.24	0.23
C(3) -H(3)	1.829	-15.625	1.0831	0.7140	0.3691	-17.68	-16.99	19.05	0.04
C(4) -C(5)	2.004	-15.198	1.4221	0.7107	0.7114	-15.66	-13.20	13.66	0.19
C(4) -H(4)	1.841	-16.376	1.0830	0.7209	0.3621	-18.20	-17.32	19.15	0.05
C(5) -C(6)	2.010	-15.168	1.4211	0.7024	0.7188	-15.61	-13.29	13.74	0.17
C(5) -C(10)	2.020	-15.044	1.4210	0.7039	0.7171	-15.67	-13.31	13.94	0.18
C(6) -C(7)	2.220	-19.222	1.3759	0.6777	0.6981	-17.83	-14.63	13.24	0.22
C(6) -H(6)	1.875	-16.831	1.0831	0.7209	0.3622	-18.45	-17.65	19.27	0.05
C(7) -C(8)	2.032	-15.600	1.4137	0.6986	0.7151	-15.82	-13.27	13.49	0.19
C(7) -H(7)	1.866	-16.297	1.0830	0.7162	0.3669	-18.19	-17.46	19.35	0.04
C(8) -C(9)	2.179	-18.044	1.3825	0.6898	0.6926	-17.55	-14.03	13.54	0.25
C(8) -H(8)	1.837	-15.787	1.0830	0.7172	0.3658	-17.91	-17.14	19.26	0.04
C(9) -C(10)	1.989	-15.028	1.4248	0.7110	0.7139	-15.56	-13.14	13.67	0.18
C(9) -C(11)	1.822	-12.327	1.4745	0.7155	0.7590	-13.42	-12.44	13.53	0.08
C(11) -C(14)	2.250	-20.098	1.3657	0.6726	0.6931	-18.39	-14.27	12.56	0.29
C(11) -H(11)	1.923	-19.633	1.0770	0.7369	0.3401	-19.76	-19.57	19.69	0.01
C(12) -H(12A)	1.756	-12.569	1.0660	0.7337	0.3323	-16.98	-16.62	21.03	0.02
C(12) -H(12B)	1.810	-15.899	1.0665	0.6947	0.3718	-17.13	-16.20	17.43	0.06
C(12) -H(12C)	1.805	-15.540	1.0660	0.7202	0.3459	-17.64	-16.96	19.05	0.04
C(13) -H(13A)	1.791	-15.569	1.0662	0.7087	0.3575	-17.05	-16.64	18.12	0.02
C(13) -H(13B)	1.790	-15.259	1.0662	0.6927	0.3736	-16.62	-16.02	17.39	0.04
C(13) -H(13C)	1.806	-13.003	1.0666	0.7383	0.3282	-17.62	-17.19	21.80	0.03
C(14) -C(15)	1.895	-12.472	1.4275	0.6934	0.7341	-14.03	-12.34	13.90	0.14
C(14) -C(16)	1.913	-12.870	1.4295	0.6924	0.7370	-14.32	-12.47	13.92	0.15

Table S6: Properties of the electron density distribution at the BCPs for the covalent bonds in **4**.

Bond	ρ(r) (e A^{-3})	$\nabla^2\rho$(r) (e A^{-5})	Rij (Å)	d_1 (Å)	d_2 (Å)	λ_1	λ_2	λ_3	E
O(1) -C(15)	2.955	-38.213	1.2328	0.7758	0.4570	-29.37	-26.74	17.90	0.10
O(2) -C(17)	2.908	-36.206	1.2274	0.7719	0.4555	-28.96	-25.69	18.45	0.13
O(3) -C(15)	2.005	-15.967	1.3782	0.8343	0.5439	-15.87	-15.33	15.23	0.04
O(3) -C(16)	1.772	-9.800	1.4335	0.8529	0.5806	-13.58	-12.39	16.17	0.10
O(4) -C(16)	1.684	-8.917	1.4330	0.8599	0.5731	-12.11	-11.79	14.99	0.03
O(4) -C(17)	1.930	-16.398	1.3805	0.8433	0.5372	-15.46	-14.81	13.87	0.04
N(1) -C(1)	1.732	-10.604	1.4766	0.8513	0.6252	-13.28	-12.23	14.90	0.09
N(1) -C(11)	1.195	-2.924	1.6467	0.9271	0.7196	-8.27	-7.72	13.06	0.07
N(1) -C(12)	1.558	-8.644	1.4960	0.9013	0.5947	-10.44	-10.05	11.85	0.04
N(1) -C(13)	1.620	-8.466	1.4959	0.8766	0.6193	-11.13	-10.87	13.53	0.02
C(1) -C(2)	2.192	-19.623	1.3699	0.6997	0.6703	-17.87	-13.82	12.06	0.29
C(1) -C(10)	2.143	-18.984	1.3959	0.7144	0.6815	-17.37	-14.11	12.50	0.23
C(2) -C(3)	1.958	-15.625	1.4261	0.7047	0.7214	-15.04	-12.38	11.79	0.22
C(2) -H(2)	1.800	-15.603	1.0831	0.7272	0.3559	-17.66	-16.62	18.67	0.06
C(3) -C(4)	2.172	-19.416	1.3850	0.7261	0.6589	-17.36	-13.53	11.48	0.28
C(3) -H(3)	1.801	-16.489	1.0831	0.7255	0.3576	-17.76	-16.81	18.08	0.06
C(4) -C(5)	2.004	-15.896	1.4236	0.7276	0.6960	-15.31	-13.01	12.43	0.18
C(4) -H(4)	1.815	-16.165	1.0830	0.7318	0.3512	-17.85	-17.12	18.81	0.04
C(5) -C(6)	1.997	-15.701	1.4210	0.7132	0.7078	-15.32	-12.74	12.35	0.20
C(5) -C(10)	2.090	-17.938	1.4052	0.6784	0.7268	-16.42	-13.78	12.26	0.19
C(6) -C(7)	2.183	-19.169	1.3852	0.6904	0.6948	-17.41	-13.64	11.88	0.28
C(6) -H(6)	1.826	-16.796	1.0832	0.7295	0.3537	-18.02	-17.21	18.43	0.05
C(7) -C(8)	1.973	-15.314	1.4256	0.7208	0.7048	-15.02	-12.29	12.00	0.22
C(7) -H(7)	1.794	-15.660	1.0832	0.7174	0.3659	-17.37	-16.45	18.16	0.06
C(8) -C(9)	2.214	-19.617	1.3734	0.6827	0.6908	-17.70	-14.06	12.15	0.26
C(8) -H(8)	1.825	-16.225	1.0831	0.7350	0.3481	-18.09	-17.34	19.20	0.04
C(9) -C(10)	2.080	-17.417	1.4043	0.6884	0.7159	-16.07	-13.64	12.30	0.18
C(9) -C(11)	1.686	-11.136	1.5100	0.7549	0.7551	-12.62	-11.16	12.64	0.13

C(11) -C(14)	1.812	-13.019	1.4706	0.7365	0.7341	-13.42	-11.89	12.29	0.13
C(11) -H(11)	1.879	-18.180	1.0770	0.7278	0.3492	-19.11	-18.42	19.35	0.04
C(12) -H(12A)	1.863	-16.182	1.0664	0.7100	0.3563	-18.20	-17.55	19.57	0.04
C(12) -H(12B)	1.814	-15.114	1.0663	0.6958	0.3705	-17.28	-16.22	18.38	0.07
C(12) -H(12C)	1.849	-15.713	1.0661	0.7124	0.3537	-17.98	-17.38	19.65	0.03
C(13) -H(13A)	1.885	-16.280	1.0663	0.7014	0.3649	-18.05	-17.35	19.13	0.04
C(13) -H(13B)	1.820	-14.587	1.0660	0.6950	0.3710	-17.11	-16.22	18.74	0.06
C(13) -H(13C)	1.820	-14.342	1.0663	0.6882	0.3782	-17.05	-15.88	18.59	0.07
C(14) -C(15)	2.016	-15.820	1.4252	0.6986	0.7266	-15.61	-12.44	12.23	0.26
C(14) -C(17)	2.033	-16.137	1.4273	0.6814	0.7458	-15.92	-12.37	12.15	0.29
C(16) -C(18)	1.761	-11.525	1.5154	0.7875	0.7279	-12.26	-12.10	12.84	0.01
C(16) -C(19)	1.722	-10.778	1.5203	0.7782	0.7421	-11.92	-11.71	12.85	0.02
C(18) -H(18A)	1.782	-14.299	1.0662	0.7143	0.3519	-16.89	-16.62	19.22	0.02
C(18) -H(18B)	1.789	-14.134	1.0664	0.7023	0.3641	-16.64	-16.30	18.80	0.02
C(18) -H(18C)	1.785	-14.332	1.0663	0.7005	0.3658	-16.69	-16.03	18.39	0.04
C(19) -H(19A)	1.801	-14.781	1.0661	0.7064	0.3596	-16.97	-16.75	18.93	0.01
C(19) -H(19B)	1.813	-14.928	1.0663	0.6942	0.3721	-16.67	-16.50	18.25	0.01
C(19) -H(19C)	1.796	-14.385	1.0660	0.6939	0.3721	-16.43	-16.18	18.23	0.02

Table S7: Properties of the electron density distribution at the BCPs for the covalent bonds in **5**.

Bond	ρ(r) (e A^{-3})	$\nabla^2\rho$(r) (e A^{-5})	Rij (Å)	d_1 (Å)	d_2 (Å)	λ_1	λ_2	λ_3	E
O(1) -C(15)	2.827	-36.929	1.2391	0.7957	0.4435	-28.27	-26.30	17.63	0.08
O(2) -C(16)	2.993	-43.367	1.2343	0.7762	0.4581	-31.38	-26.21	14.22	0.20
O(3) -C(17)	2.899	-39.425	1.2420	0.7894	0.4526	-29.42	-25.68	15.67	0.15
N(1) -C(1)	1.716	-8.146	1.4795	0.8339	0.6456	-12.29	-11.97	16.11	0.03
N(1) -C(11)	1.258	-3.461	1.6239	0.9128	0.7111	-8.42	-8.11	13.07	0.04
N(1) -C(12)	1.569	-7.936	1.4945	0.8624	0.6321	-10.6	-10.27	12.93	0.03
N(1) -C(13)	1.592	-7.486	1.5007	0.8685	0.6321	-10.74	-10.24	13.49	0.05
N(2) -C(15)	1.982	-16.744	1.4137	0.8179	0.5958	-15.96	-14.03	13.24	0.14
N(2) -C(16)	2.219	-21.739	1.3736	0.7825	0.5911	-19.13	-16.07	13.47	0.19
N(2) -C(18)	1.733	-11.484	1.4613	0.8412	0.6201	-12.22	-11.70	12.44	0.04
N(3) -C(16)	2.240	-22.059	1.3725	0.7778	0.5947	-19.86	-16.16	13.96	0.23
N(3) -C(17)	1.927	-14.979	1.4166	0.8218	0.5948	-15.13	-13.09	13.25	0.16
N(3) -C(19)	1.698	-12.855	1.461	0.8461	0.6149	-12.27	-11.67	11.09	0.05
C(1) -C(2)	2.165	-18.153	1.3697	0.6673	0.7024	-17.57	-13.19	12.6	0.33
C(1) -C(10)	2.080	-16.514	1.3983	0.6956	0.7027	-16.19	-13.34	13.01	0.21
C(2) -C(3)	1.975	-15.245	1.4277	0.7264	0.7013	-15.15	-12.50	12.40	0.21
C(2) -H(2)	1.789	-15.763	1.0831	0.7272	0.3558	-17.66	-16.69	18.59	0.06
C(3) -C(4)	2.160	-18.687	1.3862	0.679	0.7071	-17.31	-13.45	12.08	0.29
C(3) -H(3)	1.790	-15.289	1.0830	0.7297	0.3533	-17.62	-16.76	19.08	0.05
C(4) -C(5)	1.987	-15.46	1.4231	0.7052	0.7179	-15.45	-12.76	12.75	0.21
C(4) -H(4)	1.812	-16.781	1.0833	0.7349	0.3484	-18.20	-17.24	18.66	0.06
C(5) -C(6)	1.983	-15.158	1.4231	0.7024	0.7207	-15.05	-12.9	12.79	0.17
C(5) -C(10)	2.079	-17.122	1.4037	0.6945	0.7092	-16.10	-13.87	12.85	0.16
C(6) -C(7)	2.181	-19.223	1.3847	0.6956	0.6891	-17.54	-13.92	12.23	0.26
C(6) -H(6)	1.814	-17.492	1.0832	0.7322	0.351	-18.25	-17.17	17.93	0.06
C(7) -C(8)	1.983	-15.336	1.4250	0.7104	0.7146	-15.14	-12.68	12.48	0.19
C(7) -H(7)	1.825	-16.294	1.0832	0.7110	0.3722	-17.49	-16.7	17.89	0.05
C(8) -C(9)	2.145	-18.065	1.3733	0.6696	0.7036	-16.78	-13.09	11.80	0.28
C(8) -H(8)	1.785	-15.624	1.0830	0.7355	0.3475	-17.66	-17.04	19.08	0.04
C(9) -C(10)	2.126	-17.726	1.4039	0.6922	0.7116	-16.80	-13.68	12.75	0.23
C(9) -C(11)	1.726	-11.772	1.5079	0.7373	0.7706	-12.36	-11.73	12.32	0.05
C(11) -C(14)	1.784	-11.821	1.4749	0.7703	0.7046	-12.82	-11.13	12.13	0.15
C(11) -H(11)	1.843	-19.032	1.0771	0.7421	0.3350	-19.05	-18.63	18.65	0.02
C(12) -H(12A)	1.822	-16.169	1.0662	0.7220	0.3442	-18.09	-17.33	19.26	0.04

C(12) -H(12B)	1.811	-15.823	1.0660	0.7286	0.3374	-17.93	-17.53	19.63	0.02
C(12) -H(12C)	1.870	-16.972	1.0662	0.7095	0.3568	-18.23	-17.38	18.64	0.05
C(13) -H(13A)	1.837	-16.762	1.0661	0.7219	0.3443	-18.09	-17.53	18.85	0.03
C(13) -H(13B)	1.843	-15.739	1.0660	0.7291	0.3369	-18.15	-17.86	20.27	0.02
C(13) -H(13C)	1.862	-16.316	1.0661	0.7065	0.3596	-17.82	-17.37	18.87	0.03
C(14) -C(15)	2.018	-15.569	1.4246	0.6660	0.7586	-15.82	-11.64	11.89	0.36
C(14) -C(17)	2.012	-16.081	1.4184	0.6667	0.7518	-15.44	-12.09	11.45	0.28
C(18) -H(18A)	1.793	-14.748	1.0660	0.7176	0.3484	-17.28	-16.04	18.57	0.08
C(18) -H(18B)	1.799	-16.334	1.0661	0.7205	0.3455	-17.49	-16.48	17.63	0.06
C(18) -H(18C)	1.794	-15.920	1.0664	0.7217	0.3448	-17.69	-16.45	18.22	0.08
C(19) -H(19A)	1.754	-14.806	1.0669	0.7337	0.3332	-17.70	-16.00	18.90	0.11
C(19) -H(19B)	1.795	-16.069	1.0660	0.7263	0.3397	-18.01	-16.11	18.05	0.12
C(19) -H(19C)	1.768	-16.693	1.0662	0.7232	0.343	-17.93	-15.58	16.82	0.15

Table S8a: Properties of the electron density distribution at the BCPs for the covalent bonds in **6**.

Bond	ρ(r) (e A^{-3})	$\nabla^2\rho$(r) (e A^{-5})	Rij (Å)	d_1 (Å)	d_2 (Å)	λ_1	λ_2	λ_3	ε
O(1B) -C(15B)	2.768	-35.222	1.2497	0.7693	0.4805	-25.26	-23.83	13.87	0.06
O(2B) -C(18B)	2.734	-31.275	1.2468	0.7970	0.4498	-25.73	-23.63	18.08	0.09
O(1A) -C(15A)	2.704	-32.054	1.2493	0.7907	0.4586	-25.92	-22.58	16.44	0.15
O(1A) -H(2O)	0.129	3.336	1.8735	1.2260	0.6475	-0.63	-0.6	4.57	0.06
O(2A) -C(18A)	2.674	-31.517	1.2500	0.7953	0.4547	-25.48	-23.26	17.22	0.10
O(2A) -H(3O)	0.208	3.889	1.7781	1.1692	0.6088	-1.17	-1.11	6.17	0.06
O(3) -H(2O)	2.099	-25.274	0.9777	0.7516	0.2261	-32.83	-27.99	35.55	0.17
O(3) -H(1O)	2.208	-30.79	0.977	0.7657	0.2114	-37.2	-31.17	37.58	0.19
O(4) -H(4O)	2.173	-26.915	0.9772	0.7569	0.2203	-33.48	-31.25	37.82	0.07
O(4) -H(3O)	2.111	-29.685	0.977	0.7725	0.2046	-33.77	-33.43	37.52	0.01
N(1B) -C(1B)	1.763	-10.682	1.4765	0.8566	0.6198	-12.75	-12.03	14.10	0.06
N(1B) -C(11B)	1.294	-4.300	1.6253	0.9019	0.7234	-8.61	-8.37	12.68	0.03
N(1B) -C(12B)	1.622	-8.493	1.4981	0.8663	0.6319	-11.28	-10.20	12.99	0.11
N(1B) -C(13B)	1.602	-8.628	1.496	0.8769	0.6191	-10.71	-10.19	12.28	0.05
N(1A) -C(1A)	1.714	-10.476	1.4819	0.8527	0.6292	-12.17	-12.04	13.74	0.01
N(1A) -C(11A)	1.347	-6.731	1.6071	0.9159	0.6912	-9.53	-8.88	11.68	0.07
N(1A) -C(12A)	1.555	-9.647	1.5013	0.9051	0.5961	-10.43	-9.71	10.49	0.07
N(1A) -C(13A)	1.619	-8.903	1.4936	0.8650	0.6287	-10.83	-10.77	12.70	0.01
C(1B) -C(2B)	2.288	-22.207	1.3689	0.7082	0.6608	-18.80	-14.57	11.16	0.29
C(1B) -C(10B)	2.145	-18.84	1.3968	0.7332	0.6635	-16.45	-13.96	11.57	0.18
C(2B) -C(3B)	1.930	-16.006	1.429	0.7900	0.6390	-14.9	-11.55	10.45	0.29
C(2B) -H(2B)	1.827	-17.663	1.083	0.7200	0.3630	-18.15	-16.72	17.21	0.09
C(3B) -C(4B)	2.166	-19.976	1.3862	0.7637	0.6224	-17.05	-13.28	10.35	0.28
C(3B) -H(3B)	1.824	-16.66	1.0831	0.7391	0.3440	-18.29	-17.27	18.90	0.06
C(4B) -C(5B)	2.004	-15.862	1.4229	0.7151	0.7079	-15.11	-12.84	12.09	0.18
C(4B) -H(4B)	1.826	-17.109	1.0832	0.7172	0.3660	-17.64	-16.94	17.47	0.04
C(5B) -C(6B)	2.038	-17.356	1.4212	0.6999	0.7213	-15.75	-13.33	11.73	0.18
C(5B) -C(10B)	2.112	-18.342	1.403	0.6838	0.7192	-16.38	-13.87	11.92	0.18
C(6B) -C(7B)	2.238	-21.302	1.3842	0.7148	0.6694	-18.10	-14.54	11.33	0.24
C(6B) -H(6B)	1.864	-18.113	1.0833	0.7328	0.3505	-18.81	-17.56	18.25	0.07
C(7B) -C(8B)	1.979	-15.458	1.4251	0.7192	0.7059	-14.81	-12.51	11.86	0.18
C(7B) -H(7B)	1.819	-16.535	1.083	0.7256	0.3575	-17.62	-16.98	18.06	0.04
C(8B) -C(9B)	2.206	-19.84	1.3741	0.6799	0.6943	-17.26	-14.12	11.54	0.22
C(8B) -H(8B)	1.804	-15.831	1.0831	0.7275	0.3557	-17.69	-16.59	18.45	0.07
C(9B) -C(10B)	2.131	-18.959	1.4025	0.7013	0.7012	-16.51	-14.31	11.86	0.15
C(9B) -C(11B)	1.772	-12.893	1.5079	0.7288	0.7792	-13.07	-11.69	11.88	0.12
C(11B) -C(14B)	1.840	-14.757	1.4691	0.776	0.6931	-13.98	-11.94	11.17	0.17
C(11B) -H(11B)	1.849	-18.532	1.0773	0.738	0.3393	-18.97	-17.95	18.38	0.06
C(12B) -H(12A)	1.826	-16.444	1.066	0.7247	0.3413	-18.03	-17.41	18.99	0.04

C(12B) -H(12B)	1.830	-15.82	1.0661	0.6871	0.3790	-17.14	-15.8	17.12	0.08
C(12B) -H(12C)	1.796	-14.799	1.066	0.7248	0.3413	-17.58	-16.83	19.61	0.04
C(13B) -H(13A)	1.890	-17.194	1.0661	0.7389	0.3272	-19.08	-18.50	20.39	0.03
C(13B) -H(13B)	1.770	-15.39	1.066	0.6903	0.3757	-16.3	-15.76	16.67	0.03
C(13B) -H(13C)	1.822	-15.833	1.0662	0.7209	0.3452	-17.58	-17.43	19.18	0.01
C(14B) -C(15B)	2.061	-16.669	1.4207	0.699	0.7217	-16.09	-12.88	12.3	0.25
C(14B) -C(18B)	2.027	-15.867	1.4244	0.6932	0.7312	-15.39	-12.67	12.19	0.21
C(15B) -C(16B)	1.690	-10.265	1.5262	0.7671	0.7591	-11.90	-10.60	12.24	0.12
C(16B) -C(17B)	1.602	-9.094	1.5318	0.7473	0.7846	-10.47	-10.04	11.42	0.04
C(16B) -H(16A)	1.729	-13.079	1.0923	0.7125	0.3799	-15.44	-14.93	17.29	0.03
C(16B) -H(16B)	1.692	-13.659	1.0921	0.7082	0.3839	-15.41	-14.4	16.15	0.07
C(17B) -C(18B)	1.659	-9.409	1.5253	0.7334	0.7919	-11.23	-10.24	12.06	0.10
C(17B) -H(17A)	1.754	-14.122	1.092	0.6977	0.3944	-15.58	-14.89	16.34	0.05
C(17B) -H(17B)	1.806	-15.001	1.092	0.7014	0.3906	-16.35	-15.49	16.84	0.06
C(1A) -C(2A)	2.167	-18.504	1.3696	0.7238	0.6458	-16.58	-13.15	11.22	0.26
C(1A) -C(10A)	2.133	-18.597	1.3977	0.7284	0.6693	-16.77	-13.46	11.63	0.25
C(2A) -C(3A)	1.938	-14.875	1.4301	0.7569	0.6733	-14.37	-11.97	11.46	0.2
C(2A) -H(2A)	1.768	-15.755	1.0831	0.6998	0.3833	-16.35	-15.66	16.25	0.04
C(3A) -C(4A)	2.214	-20.174	1.3843	0.7607	0.6237	-16.98	-14.00	10.80	0.21
C(3A) -H(3A)	1.743	-15.559	1.0832	0.7360	0.3472	-17.37	-16.34	18.15	0.06
C(4A) -C(5A)	1.984	-15.118	1.4207	0.7333	0.6874	-14.82	-12.50	12.20	0.19
C(4A) -H(4A)	1.797	-16.103	1.0833	0.7248	0.3585	-17.12	-17.00	18.01	0.01
C(5A) -C(6A)	2.047	-17.544	1.4206	0.7224	0.6982	-15.99	-13.38	11.82	0.20
C(5A) -C(10A)	2.074	-17.648	1.4042	0.6923	0.7119	-15.89	-13.50	11.74	0.18
C(6A) -C(7A)	2.153	-19.177	1.3856	0.7518	0.6338	-16.75	-13.16	10.74	0.27
C(6A) -H(6A)	1.808	-17.27	1.0835	0.7225	0.3610	-17.78	-16.78	17.29	0.06
C(7A) -C(8A)	2.003	-15.967	1.4268	0.7265	0.7003	-14.95	-12.92	11.90	0.16
C(7A) -H(7A)	1.819	-16.783	1.0833	0.7192	0.3642	-17.41	-16.83	17.46	0.03
C(8A) -C(9A)	2.259	-21.174	1.3702	0.6818	0.6884	-17.87	-14.80	11.50	0.21
C(8A) -H(8A)	1.816	-16.663	1.0832	0.7246	0.3586	-17.78	-16.69	17.80	0.07
C(9A) -C(10A)	2.087	-17.465	1.4041	0.6681	0.7360	-15.78	-13.42	11.73	0.18
C(9A) -C(11A)	1.722	-11.955	1.5093	0.7317	0.7776	-12.25	-11.35	11.65	0.08
C(11A) -C(14A)	1.845	-14.774	1.4744	0.7766	0.6978	-13.84	-12.24	11.31	0.13
C(11A) -H(11A)	1.885	-18.840	1.0771	0.7402	0.3369	-19.37	-18.48	19.01	0.05
C(12A) -H(12D)	1.866	-17.740	1.0661	0.7077	0.3584	-18.02	-17.42	17.70	0.03
C(12A) -H(12E)	1.841	-16.854	1.066	0.7181	0.3479	-18.01	-17.42	18.58	0.03
C(12A) -H(12F)	1.830	-16.011	1.0662	0.7156	0.3506	-17.64	-17.05	18.68	0.04
C(13A) -H(13D)	1.866	-16.886	1.0663	0.7122	0.3541	-18.17	-17.58	18.87	0.03
C(13A) -H(13E)	1.809	-15.915	1.0664	0.7152	0.3512	-17.43	-17.14	18.66	0.02
C(13A) -H(13F)	1.810	-16.488	1.0661	0.7027	0.3634	-17.27	-16.52	17.31	0.05
C(14A) -C(15A)	2.055	-16.991	1.4227	0.6873	0.7354	-16.12	-12.90	12.03	0.25
C(14A) -C(18A)	2.067	-16.835	1.4235	0.6958	0.7277	-16.15	-13.11	12.42	0.23
C(15A) -C(16A)	1.712	-11.229	1.5242	0.7753	0.7489	-11.99	-11.22	11.98	0.07
C(16A) -C(17A)	1.609	-9.178	1.5326	0.7424	0.7902	-10.38	-10.27	11.47	0.01
C(16A) -H(16C)	1.684	-12.748	1.0920	0.7150	0.3770	-15.26	-14.42	16.93	0.06
C(16A) -H(16D)	1.730	-14.136	1.0922	0.7099	0.3824	-15.51	-15.08	16.45	0.03
C(17A) -C(18A)	1.640	-9.552	1.5291	0.7426	0.7865	-11.02	-10.36	11.83	0.06
C(17A) -H(17C)	1.725	-14.529	1.0920	0.7232	0.3688	-16.12	-15.46	17.05	0.04
C(17A) -H(17D)	1.725	-14.681	1.0921	0.7297	0.3624	-16.07	-15.62	17.01	0.03

Table S8b: Properties of the electron density distribution at the BCPs for the hydrogen bonds in **6**.

Bond	ρ(r) (e A^{-3})	$\nabla^2\rho$(r) (e A^{-5})	Rij (Å)	d_1 (Å)	d_2 (Å)	λ_1	λ_2	λ_3	E
H(2O) -O(1A)	0.129	3.336	1.8735	0.6475	1.2260	-0.63	-0.60	4.57	0.06
H(3O) -O(2A)	0.208	3.889	1.7781	0.6088	1.1692	-1.17	-1.11	6.17	0.06
H(4O) -X3_O(1B)	0.139	3.266	1.8674	0.6543	1.2131	-0.71	-0.69	4.66	0.03
H(1O) -X4_O(4)	0.123	3.216	1.8905	0.6554	1.2351	-0.61	-0.51	4.34	0.19

Symmetry codes: X3) SYMM 3 - X, - Y, - Z, translation 0 1 0;
X4) SYMM 4 + X, 1/2 - Y, 1/2 + Z, translation 0 -0.5 0.5.

Atomic Charges for 1-6

Table S9: The atomic charges of **1.**

Atom	Hirshfeld		Bader		
	Population	Net Charge	Population [e]	QTAIM charge [e]	Atomic Lagrangian [au]
1 O(1A)	8.193	-0.193	8.912	-0.912	7.959 E-06
2 O(1B)	8.164	-0.164	8.866	-0.866	-3.083 E-04
3 N(1)	7.104	-0.104	7.888	-0.888	3.145 E-03
4 C(1)	5.999	0.001	5.755	0.245	-1.160 E-02
5 C(2)	6.048	-0.048	6.080	-0.080	-4.864 E-03
6 C(3)	6.047	-0.047	6.105	-0.105	7.146 E-04
7 C(4)	6.030	-0.030	6.116	-0.116	-2.105 E-03
8 C(5)	6.010	-0.010	5.998	0.002	-1.206 E-02
9 C(6)	6.032	-0.032	6.052	-0.052	-4.417 E-03
10 C(7)	6.035	-0.035	6.101	-0.101	-3.486 E-03
11 C(8)	6.065	-0.064	6.152	-0.152	-5.448 E-03
12 C(9)	5.988	0.012	5.932	0.068	-2.289 E-03
13 C(10)	6.025	-0.025	6.033	-0.033	-1.902 E-02
14 C(11)	5.991	0.009	6.032	-0.032	7.493 E-03
15 C(12)	6.070	-0.070	6.120	-0.120	-1.495 E-03
16 C(13)	6.059	-0.059	5.915	0.085	-7.504 E-04
17 C(14)	6.061	-0.061	6.128	-0.128	7.506 E-05
18 C(15A)	5.882	0.118	5.192	0.808	7.106 E-03
19 C(15B)	5.864	0.136	5.152	0.848	8.046 E-03
20 C(16A)	6.025	-0.025	5.993	0.007	3.133 E-03
21 C(16B)	6.046	-0.046	5.917	0.083	8.318 E-03
22 C(17A)	6.052	-0.052	6.093	-0.093	5.148 E-03
23 C(17B)	6.055	-0.055	6.120	-0.120	4.991 E-03
24 C(18A)	6.056	-0.056	6.132	-0.132	6.865 E-04
25 C(18B)	6.068	-0.068	6.052	-0.052	4.819 E-03
26 C(19A)	6.073	-0.073	6.074	-0.074	-1.481 E-03
27 C(19B)	6.032	-0.032	6.154	-0.154	3.846 E-03
28 C(20A)	6.066	-0.066	6.172	-0.172	2.615 E-03
29 C(20B)	6.050	-0.050	6.171	-0.171	1.869 E-04
30 C(21A)	6.052	-0.052	6.151	-0.151	3.218 E-03
31 C(21B)	6.053	-0.053	6.248	-0.248	1.233 E-03
32 H(12A)	0.967	0.033	0.890	0.120	-2.071 E-05
33 H(12B)	0.979	0.021	0.847	0.153	2.010 E-05
34 H(12C)	0.943	0.057	0.868	0.132	2.671 E-05
35 H(13A)	1.013	-0.013	0.957	0.043	1.339 E-04
36 H(13B)	0.977	0.023	0.938	0.062	3.603 E-05
37 H(13C)	1.016	-0.016	0.963	0.037	-3.862 E-05
38 H(17A)	0.947	0.053	0.892	0.108	-5.339 E-05
39 H(17B)	0.915	0.085	0.771	0.229	8.144 E-05
40 H(18A)	0.941	0.058	0.866	0.134	2.541 E-05
41 H(18B)	0.910	0.090	0.815	0.185	-4.894 E-06

42 H(19A)	0.942	0.058	0.890	0.110	1.958 E-05
43 H(19B)	0.906	0.094	0.860	0.140	-1.363 E-05
44 H(20A)	0.930	0.070	0.874	0.126	4.635 E-06
45 H(20B)	0.889	0.111	0.866	0.134	1.110 E-05
46 H(21A)	0.948	0.052	0.880	0.120	5.464 E-06
47 H(21B)	0.927	0.073	0.845	0.155	7.753 E-05
48 H(2)	0.940	0.060	0.858	0.142	3.385 E-04
49 H(3)	0.912	0.088	0.854	0.146	1.449 E-06
50 H(4)	0.934	0.066	0.906	0.094	-5.407 E-07
51 H(6)	0.928	0.072	0.887	0.113	-8.755 E-06
52 H(7)	0.933	0.067	0.897	0.103	-5.838 E-06
53 H(8)	0.954	0.046	0.898	0.102	-4.586 E-05
54 H(11)	0.945	0.055	0.862	0.138	5.365 E-04

Table S10: The atomic charges of **2**.

**Atom**	**Hirshfeld**		**Bader**		
	**Population**	**Net Charge**	**Population [e]**	**QTAIM charge [e]**	**Atomic Lagrangian [au]**
O(1)	8.188	-0.188	8.953	-0.953	-3.247 E-05
N(1)	7.035	-0.035	7.751	-0.751	-2.961 E-03
C(1)	6.027	-0.027	5.817	0.183	2.107 E-03
C(2)	6.079	-0.079	6.141	-0.141	-8.217 E-04
C(3)	6.029	-0.029	6.050	-0.050	-1.979 E-03
C(4)	6.049	-0.049	6.106	-0.106	-6.607 E-04
C(5)	6.032	-0.032	6.007	-0.007	-6.365 E-03
C(6)	6.008	-0.008	6.081	-0.081	-9.001 E-04
C(7)	6.031	-0.031	6.033	-0.033	-5.576 E-04
C(8)	6.081	-0.081	6.183	-0.183	-1.250 E-03
C(9)	6.079	-0.079	6.067	-0.067	-1.739 E-03
C(10)	6.060	-0.060	6.057	-0.057	2.654 E-03
C(11)	5.925	0.075	5.261	0.739	-3.796 E-04
C(12)	6.039	-0.039	5.897	0.103	9.204 E-03
C(13)	6.053	-0.053	5.867	0.133	1.142 E-02
H(2)	0.881	0.119	0.823	0.177	-5.092 E-06
H(3)	0.941	0.059	0.906	0.094	1.870 E-06
H(4)	0.918	0.082	0.880	0.120	1.042 E-05
H(6)	0.911	0.089	0.837	0.163	1.522 E-05
H(7)	0.951	0.049	0.927	0.073	8.431 E-06
H(8)	0.965	0.035	0.898	0.102	6.391 E-06
H(12A)	0.947	0.053	0.906	0.094	-1.517 E-06
H(12B)	0.937	0.063	0.937	0.063	-5.106 E-05
H(12C)	0.940	0.060	0.875	0.125	2.578 E-05
H(13A)	0.918	0.082	0.892	0.108	2.930 E-05
H(13B)	0.983	0.017	0.976	0.024	5.763 E-06
H(13C)	0.989	0.011	0.968	0.032	2.239 E-04
H(11)	1.005	-0.005	0.895	0.105	2.941 E-05

Table S11: The atomic charges of **3**.

**Atom**	**Hirshfeld**		**Bader**		
	**Population**	**Net Charge**	**Population [e]**	**QTAIM charge [e]**	**Atomic Lagrangian [au]**
N(1)	7.100	-0.100	7.906	-0.906	-6.543 E-03
N(2)	7.232	-0.232	7.910	-0.910	-5.465 E-06
N(3)	7.223	-0.223	7.910	-0.910	5.481 E-06
C(1)	5.946	0.054	5.665	0.335	3.405 E-03
C(2)	6.043	-0.043	6.094	-0.094	4.664 E-04
C(3)	6.011	-0.011	6.006	-0.006	5.218 E-04
C(4)	6.002	-0.002	6.038	-0.038	1.054 E-03

C(5)	5.982	0.018	5.949	0.051	4.937 E-03
C(6)	6.027	-0.027	6.072	-0.072	-1.961 E-03
C(7)	6.029	-0.029	6.057	-0.057	-4.412 E-04
C(8)	6.025	-0.025	6.056	-0.056	6.597 E-04
C(9)	5.985	0.015	5.956	0.044	5.557 E-04
C(10)	5.982	0.018	5.980	0.020	-1.608 E-04
C(11)	5.979	0.021	5.966	0.034	5.154 E-03
C(12)	6.063	-0.063	5.971	0.029	5.408 E-03
C(13)	6.079	-0.079	5.993	0.007	1.210 E-02
C(14)	6.058	-0.058	6.044	-0.044	7.703 E-05
C(15)	5.974	0.026	5.348	0.652	-1.467 E-04
C(16)	5.994	0.006	5.368	0.632	1.107 E-03
H(12A)	1.001	-0.001	0.911	0.089	-2.067 E-05
H(12B)	0.945	0.055	0.931	0.069	-3.181 E-05
H(12C)	0.962	0.038	0.895	0.105	-1.757 E-04
H(13A)	0.933	0.067	0.888	0.112	2.945 E-06
H(13B)	0.949	0.051	0.942	0.058	7.486 E-05
H(13C)	1.007	-0.007	0.901	0.099	-8.506 E-05
H(2)	0.934	0.066	0.913	0.087	-5.400 E-05
H(3)	0.921	0.079	0.899	0.101	-2.074 E-05
H(4)	0.917	0.083	0.885	0.115	1.295 E-05
H(6)	0.914	0.086	0.871	0.129	-4.446 E-06
H(7)	0.930	0.070	0.906	0.094	-6.616 E-06
H(8)	0.937	0.063	0.906	0.094	-5.201 E-05
H(11)	0.918	0.082	0.839	0.161	9.516 E-05

Table S12: The atomic charges of **4**.

Atom	Hirshfeld		Bader		
	**Population**	**Net Charge**	**Population [e]**	**QTAIM charge [e]**	**Atomic Lagrangian [au]**
O(1)	8.258	-0.258	8.984	-0.984	-4.864 E-04
O(2)	8.286	-0.286	9.029	-1.029	7.394 E-04
O(3)	8.207	-0.207	9.032	-1.032	9.663 E-04
O(4)	8.139	-0.139	8.966	-0.966	-5.562 E-04
N(1)	6.882	0.118	7.820	-0.820	3.758 E-03
C(1)	5.915	0.085	5.702	0.298	6.598 E-03
C(2)	6.021	-0.021	6.076	-0.076	9.091 E-04
C(3)	6.018	-0.018	6.154	-0.154	-1.283 E-03
C(4)	6.052	-0.052	6.109	-0.109	4.067 E-03
C(5)	6.005	-0.005	5.910	0.090	-1.277 E-03
C(6)	6.060	-0.060	6.143	-0.143	-1.809 E-03
C(7)	6.040	-0.040	6.070	-0.070	1.786 E-03
C(8)	6.079	-0.079	6.147	-0.147	7.706 E-04
C(9)	5.997	0.003	5.980	0.020	-4.464 E-03
C(10)	6.009	-0.009	6.062	-0.062	-7.864 E-03
C(11)	5.912	0.088	5.700	0.300	-2.600 E-03
C(12)	6.006	-0.006	5.823	0.177	8.809 E-03
C(13)	6.076	-0.076	5.883	0.117	-7.531 E-04
C(14)	6.138	-0.138	6.204	-0.204	8.194 E-04
C(15)	5.899	0.101	4.762	1.238	-4.407 E-04
C(16)	5.920	0.080	5.244	0.756	2.461 E-02
C(17)	5.860	0.140	4.722	1.278	1.077 E-02
C(18)	6.120	-0.120	6.166	-0.166	4.774 E-03
C(19)	6.104	-0.104	6.082	-0.082	5.380 E-03
H(2)	0.917	0.083	0.845	0.155	1.961 E-05
H(3)	0.916	0.084	0.861	0.139	1.232 E-05
H(4)	0.922	0.078	0.842	0.158	-7.072 E-06
H(6)	0.909	0.091	0.834	0.166	-1.615 E-06
H(7)	0.936	0.064	0.902	0.098	-6.003 E-06
H(8)	0.940	0.060	0.857	0.143	-5.784 E-06
H(12A)	0.950	0.050	0.921	0.079	4.979 E-05

H(12B)	0.945	0.055	0.909	0.091	1.216 E-04
H(12C)	0.942	0.058	0.894	0.106	-4.866 E-05
H(13A)	0.962	0.038	0.907	0.093	-6.791 E-05
H(13B)	0.961	0.039	0.932	0.068	6.692 E-05
H(13C)	0.962	0.038	0.961	0.039	6.751 E-05
H(18A)	0.961	0.039	0.899	0.101	-2.509 E-05
H(18B)	8.258	-0.258	0.941	0.059	2.355 E-05
H(18C)	8.286	-0.286	0.926	0.074	-2.713 E-06
H(19A)	8.207	-0.207	0.944	0.056	4.749 E-05
H(19B)	8.139	-0.139	0.964	0.036	8.847 E-06
H(19C)	6.882	0.118	0.966	0.034	2.513 E-05
H(11)	5.915	0.085	0.876	0.124	-3.271 E-05

Table S13: The atomic charges of **5**.

	**Hirshfeld**		**Bader**		
**Atom**	**Population**	**Net Charge**	**Population [e]**	**QTAIM charge [e]**	**Atomic Lagrangian [au]**
1 O(1)	8.239	-0.239	9.036	-1.036	-2.349 E-05
2 O(2)	8.327	-0.327	9.092	-1.092	-4.557 E-05
3 O(3)	8.277	-0.277	9.072	-1.072	9.551 E-05
4 N(1)	6.951	0.049	7.846	-0.846	6.978 E-03
5 N(2)	7.110	-0.110	8.022	-1.022	3.680 E-03
6 N(3)	7.055	-0.055	7.964	-0.964	8.864 E-04
7 C(1)	5.975	0.025	5.733	0.267	4.807 E-03
8 C(2)	6.024	-0.024	6.127	-0.127	-1.117 E-03
9 C(3)	6.057	-0.057	6.094	-0.094	-1.359 E-04
10 C(4)	6.017	-0.017	6.095	-0.095	1.269 E-03
11 C(5)	6.007	-0.007	6.019	-0.019	-1.624 E-03
12 C(6)	6.009	-0.009	6.115	-0.115	-6.943 E-05
13 C(7)	6.025	-0.025	6.040	-0.040	2.518 E-03
14 C(8)	6.042	-0.042	6.090	-0.090	-2.573 E-03
15 C(9)	6.067	-0.067	6.086	-0.086	2.663 E-03
16 C(10)	6.033	-0.033	6.026	-0.026	-2.053 E-03
17 C(11)	5.977	0.023	5.864	0.136	9.921 E-03
18 C(12)	6.008	-0.008	5.950	0.050	7.618 E-03
19 C(13)	6.078	-0.078	6.036	-0.036	-1.323 E-02
20 C(14)	6.175	-0.175	6.055	-0.055	5.849 E-03
21 C(15)	5.924	0.076	4.920	1.080	-2.767 E-03
22 C(16)	5.902	0.098	4.560	1.440	-1.930 E-03
23 C(17)	5.953	0.047	4.983	1.017	-1.538 E-02
24 C(18)	6.088	-0.088	6.090	-0.090	-2.352 E-04
25 C(19)	6.046	-0.046	6.131	-0.131	1.008 E-02
26 H(2)	0.917	0.083	0.856	0.144	-2.843 E-05
27 H(3)	0.931	0.069	0.867	0.133	-1.451 E-06
28 H(4)	0.903	0.097	0.824	0.176	3.488 E-05
29 H(6)	0.869	0.131	0.779	0.221	2.126 E-05
30 H(7)	0.911	0.089	0.884	0.116	1.069 E-05
31 H(8)	0.925	0.075	0.840	0.160	-1.817 E-05
32 H(11)	0.939	0.061	0.846	0.154	1.540 E-04
33 H(12A)	0.934	0.066	0.871	0.129	-4.355 E-05
34 H(12B)	0.946	0.054	0.849	0.151	-1.231 E-04
35 H(12C)	0.937	0.063	0.889	0.111	-4.720 E-05
36 H(13A)	0.944	0.056	0.839	0.161	-9.668 E-04
37 H(13B)	0.971	0.029	0.876	0.124	-1.067 E-04
38 H(13C)	0.959	0.041	0.927	0.073	1.500 E-06
39 H(18A)	0.966	0.034	0.870	0.130	-5.223 E-06
40 H(18B)	0.926	0.074	0.800	0.200	-1.591 E-04
41 H(18C)	0.932	0.068	0.830	0.170	-2.033 E-05
42 H(19A)	0.928	0.072	0.783	0.217	2.588 E-05
43 H(19B)	0.920	0.080	0.774	0.226	-2.409 E-04
44 H(19C)	0.875	0.125	0.735	0.265	1.874 E-05

Table S14: The atomic charges of **6**.

Atom	Hirshfeld		Bader		
	**Population**	**Net Charge**	**Population [e]**	**QTAIM charge [e]**	**Atomic Lagrangian [au]**
1 O(1B)	8.286	-0.286	8.964	-0.964	-1.590 E-05
2 O(2B)	8.300	-0.300	9.077	-1.077	1.568 E-04
3 O(1A)	8.196	-0.196	8.989	-0.989	3.509 E-04
4 O(2A)	8.179	-0.179	8.996	-0.996	1.033 E-03
5 O(3)	8.264	-0.264	9.168	-1.168	2.772 E-04
6 O(4)	8.348	-0.348	9.193	-1.193	-3.828 E-03
7 N(1B)	7.004	-0.004	7.981	-0.981	-2.297 E-03
8 N(1A)	6.938	0.062	7.921	-0.921	5.108 E-03
9 C(1B)	6.044	-0.044	5.960	0.040	-5.693 E-04
10 C(2B)	6.060	-0.060	6.198	-0.198	-2.098 E-03
11 C(3B)	6.069	-0.069	6.173	-0.173	3.786 E-03
12 C(4B)	6.042	-0.042	5.950	0.050	-1.756 E-03
13 C(5B)	6.038	-0.038	5.987	0.013	3.091 E-03
14 C(6B)	6.065	-0.065	6.214	-0.214	6.212 E-03
15 C(7B)	6.051	-0.051	6.080	-0.080	2.352 E-03
16 C(8B)	6.083	-0.083	6.135	-0.135	2.389 E-03
17 C(9B)	6.026	-0.026	5.982	0.018	2.391 E-03
18 C(10B)	6.037	-0.037	5.955	0.045	2.014 E-03
19 C(11B)	6.000	0.000	5.950	0.050	6.507 E-03
20 C(12B)	6.064	-0.064	5.971	0.029	8.624 E-03
21 C(13B)	6.056	-0.056	5.987	0.013	2.048 E-03
22 C(14B)	6.134	-0.134	6.125	-0.125	1.945 E-03
23 C(15B)	5.940	0.060	5.247	0.753	-5.928 E-03
24 C(16B)	6.111	-0.111	6.187	-0.187	-2.244 E-03
25 C(17B)	6.148	-0.148	6.181	-0.181	3.888 E-03
26 C(18B)	5.981	0.019	5.297	0.703	-2.468 E-03
27 C(1A)	6.017	-0.017	5.931	0.069	-5.112 E-04
28 C(2A)	6.032	-0.032	6.068	-0.068	4.244 E-03
29 C(3A)	6.046	-0.046	6.188	-0.188	1.789 E-03
30 C(4A)	6.057	-0.057	6.061	-0.061	7.756 E-04
31 C(5A)	6.025	-0.025	5.977	0.023	-3.130 E-03
32 C(6A)	6.027	-0.027	6.161	-0.161	4.542 E-03
33 C(7A)	6.066	-0.066	6.051	-0.051	-9.626 E-04
34 C(8A)	6.074	-0.074	6.111	-0.111	8.705 E-04
35 C(9A)	6.058	-0.058	5.988	0.012	2.248 E-03
36 C(10A)	6.065	-0.065	6.070	-0.070	-6.698 E-03
37 C(11A)	5.980	0.020	5.912	0.088	-4.008 E-03
38 C(12A)	6.019	-0.019	5.907	0.093	7.220 E-03
39 C(13A)	5.993	0.007	5.869	0.131	-5.619 E-03
40 C(14A)	6.096	-0.096	6.043	-0.043	4.836 E-03
41 C(15A)	5.920	0.080	5.227	0.773	-1.699 E-03
42 C(16A)	6.087	-0.087	6.148	-0.148	6.323 E-03
43 C(17A)	6.083	-0.083	6.255	-0.255	-3.179 E-05
44 C(18A)	5.914	0.086	5.234	0.766	-1.933 E-03
45 H(2B)	0.889	0.111	0.830	0.170	5.049 E-06
46 H(3B)	0.921	0.079	0.819	0.181	-9.580 E-06
47 H(4B)	0.904	0.096	0.857	0.143	-5.313 E-06
48 H(6B)	0.912	0.088	0.828	0.172	8.088 E-07
49 H(7B)	0.911	0.089	0.839	0.161	-4.914 E-06
50 H(8B)	0.928	0.072	0.844	0.156	8.351 E-06
51 H(11B)	0.955	0.045	0.867	0.133	-1.407 E-04
52 H(12A)	0.943	0.057	0.863	0.137	-1.117 E-04
53 H(12B)	0.955	0.045	0.940	0.060	3.763 E-04
54 H(12C)	0.979	0.021	0.875	0.125	6.087 E-06
55 H(13A)	0.959	0.041	0.835	0.165	4.798 E-05
56 H(13B)	0.932	0.068	0.908	0.092	6.520 E-05
57 H(13C)	0.949	0.051	0.880	0.120	8.244 E-06
58 H(16A)	0.944	0.056	0.890	0.110	9.172 E-06
59 H(16B)	0.911	0.089	0.867	0.133	1.195 E-05
60 H(17A)	0.941	0.059	0.931	0.069	1.176 E-05

61 H(17B)	0.959	0.041	0.944	0.056	6.199 E-04
62 H(2A)	0.860	0.140	0.821	0.179	-2.408 E-05
63 H(3A)	0.906	0.094	0.817	0.183	-1.068 E-05
64 H(4A)	0.902	0.098	0.831	0.169	-4.553 E-06
65 H(6A)	0.893	0.107	0.832	0.168	-3.574 E-06
66 H(7A)	0.890	0.110	0.818	0.182	8.720 E-06
67 H(8A)	0.908	0.092	0.819	0.181	4.340 E-06
68 H(11A)	0.986	0.014	0.886	0.114	1.382 E-04
69 H(12D)	0.912	0.088	0.858	0.142	-5.449 E-05
70 H(12E)	0.941	0.059	0.867	0.133	1.784 E-04
71 H(12F)	0.954	0.046	0.870	0.130	-6.925 E-05
72 H(13D)	0.974	0.026	0.927	0.073	6.811 E-04
73 H(13E)	0.955	0.045	0.893	0.107	-2.276 E-04
74 H(13F)	0.911	0.089	0.872	0.128	2.747 E-04
75 H(16C)	0.929	0.071	0.874	0.126	-3.738 E-05
76 H(16D)	0.920	0.080	0.852	0.148	-2.049 E-04
77 H(17C)	0.915	0.085	0.859	0.141	5.848 E-04
78 H(17D)	0.896	0.104	0.800	0.200	1.059 E-06
79 H(2O)	0.874	0.126	0.400	0.600	4.994 E-04
80 H(1O)	0.814	0.186	0.359	0.641	-3.909 E-06
81 H(4O)	0.835	0.165	0.411	0.589	2.859 E-06
82 H(3O)	0.907	0.093	0.412	0.588	5.918 E-04

Table S15. Summary of interatomic distances, bond paths and charge density and Laplacians at (3,-1) critical points for (a) the N---C interaction/bond and (b) the attacked C=C bond for 1, 3-6 representing different stages of a Michael reaction.

N···C(=C).

	**Bond length / Å**	**Bond path / Å**	***ρ*(r) (*e* A^{-3})**	**∇^{2}*ρ*(r) (*e* A^{-5})**
**1**	2.6758(4)	2.6953	0.130	+1.688
**3**	2.4163(2)	2.4180	0.213	+2.199
**4**	1.6467(5)	1.6467	1.195	-2.924
**5**	1.6237(9)	1.6239	1.258	-3.461
**6B**	1.6252(7)	1.6253	1.294	-4.300
**6A**	1.6070(6)	1.6071	1.347	-6.731

(N···)C=C.

	**Bond length / Å**	**Bond path / Å**	***ρ*(r) (*e* A^{-3})**	**∇^{2}*ρ*(r) (*e* A^{-5})**
**1**	1.3509(3)	1.3509	2.278	-19.872
**3**	1.3685(18)	1.3657	2.250	-20.098
**4**	1.4703(5)	1.4706	1.812	-13.019
**5**	1.4737(8)	1.4749	1.784	-11.821
**6B**	1.4687(6)	1.4691	1.840	-14.757
**6A**	1.4737(8)	1.4744	1.845	-14.774

3. QTAIM Calculations.

The QTAIM properties for the N---C interaction/bond of **1**-**6** and **S1** and **S2** were calculated at the (B3LYP/6-311+G(d,p) level in Gaussian[S17] and analysed with AIMA11.[S19] A comparison of the experimental and calculated charge density and Laplacians at the N···C BCP are shown in Table S16. A plot of calculated Laplacian v N---C separation is shown in Figure S27. A plot of the energy density at the BCP against N---C separation is given in Figure S28.

Table S16. Experimental and Calculated QTAIM parameters for at the bond critical point for the Me_2N---C peri bond in **1-6** and **S1-S2**

	Experimental ρ(r) (e Å^{-3})	Calculated ρ(r) (e Å^{-3})	Experimental $\nabla^2\rho(r)$ (e Å^{-5})	Calculated $\nabla^2\rho(r)$ (e Å^{-5})	Calculated Energy density, H (a.u.)	Calculated Delocalisation Index (DI).
**1**	0.130	0.133	+1.688	+1.558	+ 0.001	0.08
**2**	0.192	0.192	+1.971	+1.931	–0.001	0.14
**3**	0.213	0.234	+2.199	+1.992	–0.004	0.19
**S1**	-	0.332	-	+2.262	–0.011	0.23
**S2**	-	0.564	-	+2.129	–0.039	0.36
**4**	1.195	1.180	–2.924	–5.054	–0.174	0.71
**5**	1.258	1.239	–3.461	–6.163	–0.192	0.72
**6B**	1.294	1.240	–4.300	–6.257	–0.192	0.72
**6A**	1.347	1.278	–6.731	–6.874	–0.203	0.71

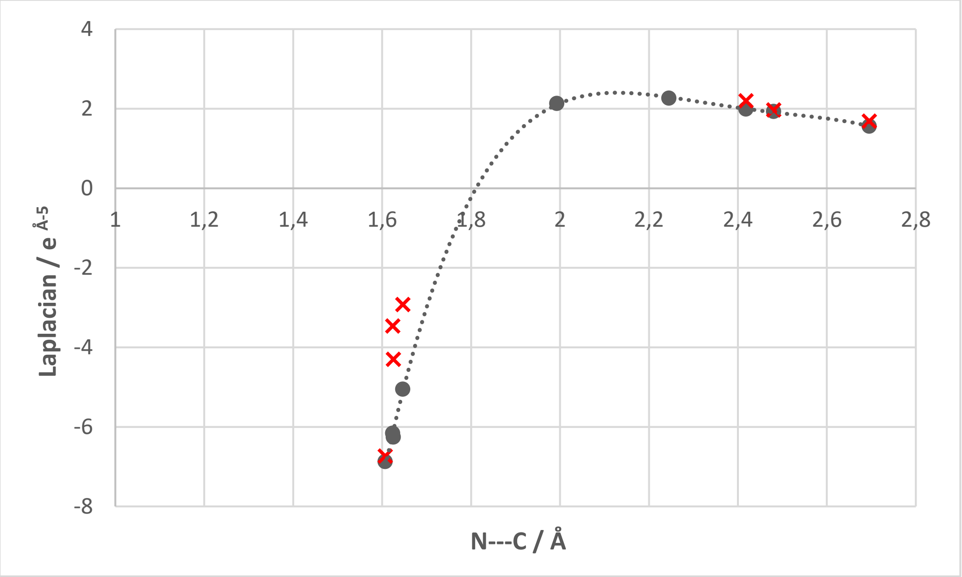

Figure S27. Plot of calculated Laplacian for **1**-**6**, **S1** and **S2** against Me_2N---C separation (black dots), with experimental values for **1**-**6** added (red crosses).

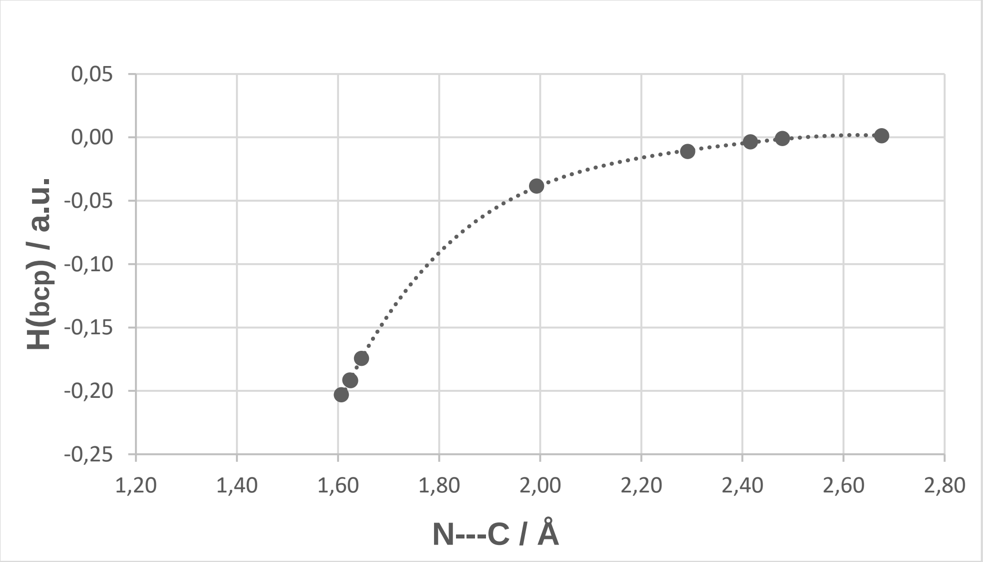

Figure S28. Plot of the energy density at the Me2 N---C bond critical point, H(BCP), against $Me_2N\cdots C$ separation for **1**-**6**, **S1** and **S2**.

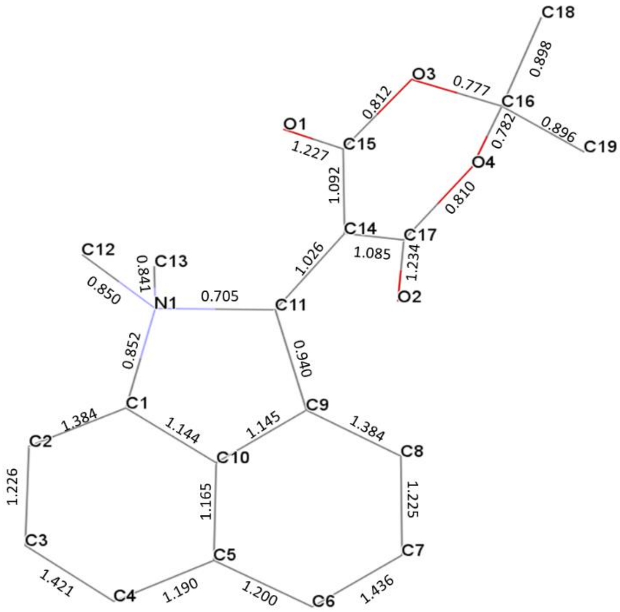

Figure S29. Calculated Delocalisation Indexes (DI) for each bond between non-H atoms in **4**. The DI's in the naphthalene ring reflect a degree of bond alternation around the ring. The peri N-C bond has DI 0.705, and the "broken" alkene bond, C11-C14, shows a DI of 1.026.

4. Solid State NMR.

All solid state NMR (SS-NMR) experiments were performed on an 11.7 T Bruker Avance III NMR spectrometer, operating at ^{1}H, ^{13}C and ^{15}N Larmor frequencies of 500.1, 125.8 and 50.7 MHz respectively. A Bruker 3.2 mm triple-resonance magic angle spinning (MAS) probe was used to facilitate spinning frequencies of 11 kHz. The determined ^{13}C and ^{15}N $\pi/2$ pulses of 5.0 µs were utilised, which represent a nutation frequency (v_1) equal to 50 kHz. Ramped cross polarisation was employed to all ^{13}C and ^{15}N spectra, with a contact time of 1.0 ms. A ^{1}H $\pi/2$ pulse of 2.5 µs (100 kHz) and a recycle delay of 16 seconds were employed to allow the probe's coil to cool down after SPINAL-64 heteronuclear decoupling ($v_1(^{1}H)$ = 120 kHz). A minimum of 800 transients were recorded for each spectrum.

The ^{15}N chemical shifts were referenced indirectly to neat liquid nitromethane (CH_3NO_2, 0 ppm) by using the secondary reference of powdered [^{15}N]histidine (δ_{iso} = −333.1, −204.3 and −191.0 ppm). To convert to the chemical shift scale employed by protein NMR (NH_3 (*l*), −50 ºC) it is necessary to add 379.5 ppm to the given scale. The ^{13}C chemical shifts were referenced to the primary reference of neat tetramethylsilane (*l*, TMS, $Si(CH_3)_4$, 0 ppm). [S20]

The ^{13}C-^{15}N ^{1}J-coupling measurements were performed as previously described by Hung and co-workers using the sequence given in Figure S30.[S21] Here, an initial CPMAS to ^{15}N was completed, before an identical π refocussing pulse on both ^{15}N and ^{13}C channels during a varying τ period was utilised. Tau (τ) was incremented from 0 to 300 ms. A total of 32-64 scans were collected per increment, with 60 increments, and a recycle delay of 16 scans giving a total experiment time of ~18 hours. All experiments where completed from the perspective of the ^{15}N nuclei, which is coupled to 99 % ^{13}C. We have previously shown it is possible to measure ^{1}J-couplings from a ^{13}C which is coupled to quadrupolar nuclei ($^{1}J^{13}C^{17}O$).[S24] However, larger scalar couplings are typically required to isolate the ^{1}J terms from the quadrupolar effects.

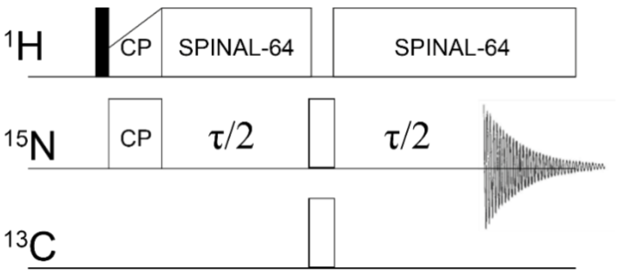

Figure S30: Cross polarisation heteronuclear spin-echo experiment utilised in the ^{13}C-^{15}N ^{1}J coupling measurements.

All spectra were made using DMFit[S22], and the CASTEP derived MAS NMR parameters were simulated with SIMPSON[S23] with the experimental parameters discussed above. The SIMPSON simulation package was also used to derive the $^{1}J_{NC}$ couplings, as this package does not incorporate T_2, then these outputs were multiplied by the homonuclear echo determined T_2' ($\exp(-\tau/T_2')$). The output of these simulations with varying $^{1}J_{NC}$ values and for two different T_2' values are given in Figure S31. We have previously discussed this methodology in further detail.[S24]

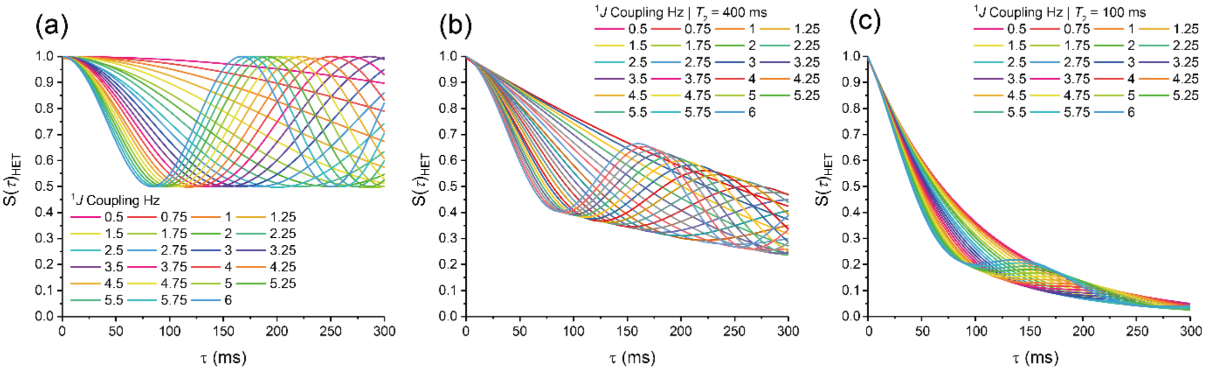

Figure S31: (a) The Simpson simulated ^{15}N-^{13}C heteronuclear spin-echo intensities showing the $^{1}J_{NC}$ cosine modulations over a 0.5 to 6 Hz range (these follow a $\cos(\pi J_{NC}\tau)$ function, no T_2' contributions). The weighting of these cosine oscillations with T_2' ($\exp(-\tau/T_2')$) values of (b) 400 and (c) 100 ms. These simulations show that for small ^{1}J-couplings that T_2' causes the cosine modulated exponential decays to converge. This is not an issue for

^{15}N, which has narrow native linewidths and couplings and can be overcome by using faster MAS for other nuclei.

Density Functional Theory for Solid State NMR Studies.

All density functional theory (DFT) calculations used the CASTEP 16.1 code which employs Kohn-Sham DFT methodology using periodic plane-waves under the ultrasoft pseudopotential approximation.[S25] The generalized gradient approximation for the exchange-correlation energy was employed using the Perdew-Burke-Ernzerhof (PBE) functional.[S26] The pseudopotentials were generated 'on-the-fly' using the standard Materials Studio pseudo-atom definitions (Accelrys, San Diego CA, USA).

The calculation was converged with respect to basis-set size and Brillouin zone k-point sampling to at least an accuracy of 0.4 mH (~2 x10^{-6} % of total energy) per atom for each of the systems under investigation. To confirm this level of energy convergence was sufficient to produce accurate ionic forces, energy minimization with respect to their ionic positions was repeated with increasing plane wave cut off energy and density of k points within the Monkhorst–Pack Brillouin zone grid. This level of convergence was achieved using plane-wave cutoff energy of 1400 eV for the systems, and by invoking k-point Monkhorst-Pack grids of 2 × 3 × 2. [S27]

To calculate isotropic chemical shifts were determined using the following references; σ_{ref} = 170 ppm for ^{13}C and −153 ppm for ^{15}N.

Cross Polarisation ^{13}C and ^{15}N Solid State NMR

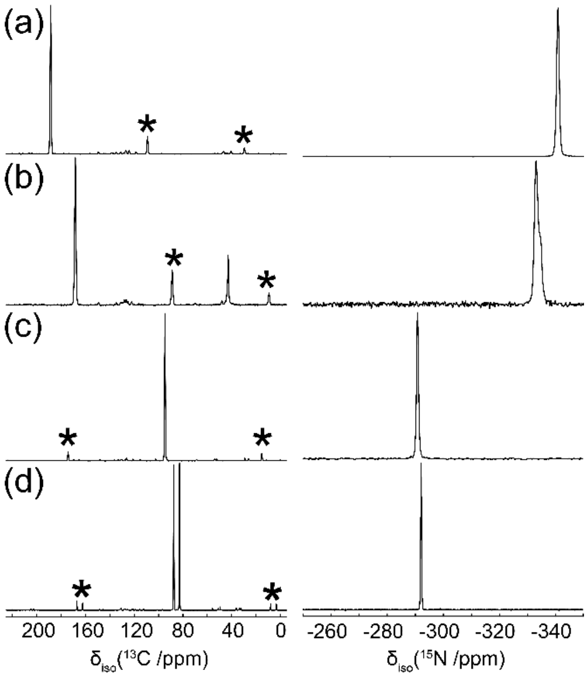

Figure S32: The proton (^{1}H->^{13}C) cross polarised, and 120 kHz ^{1}H decoupled, ^{13}C and ^{15}N solid state NMR of the doubly isotopically labelled structures (a) **2**, (b) **3**, (c) **4** and (d) **6** (for which there are two crystallographically independent molecules). * represents the location of the spinning sidebands. The ^{15}N MAS NMR of (b) shows some broadening to one side of the resonance. A bond between peri substituents is not formed, which allows for a small degree of rotation about the N-C(ring) bond at room temperature. This rotation and the variation in potential overlap between the nitrogen lone pair and aromatic π system will lead to a distribution of environments, which could ead to broadening. Alternatively, there could be some disorder in the orientation of this group between different regions of the crystal.

The powder ^{13}C and ^{15}N solid state NMR of the labelled samples were achieved using a proton (^{1}H) cross-polarization (CP) magic angle spinning (MAS) experiment before proton decoupled acquisition. Hence, the data presented is no longer quantitative due to CP being directly affected by internuclear ^{1}H -^{13}C/^{15}N distances and this lack of quantitation is enhanced by the isotopic labelling of the peri environments with ^{13}C and ^{15}N. The ^{13}C CPMAS NMR spectra of compounds **2** and **3** (Figure S32) both show a single distinct environment at 188.6 and 168.4 ppm respectively, these are consistent with the two polar groups (CH=O and CH=C(CN)$_2$), respectively.

Conversely, the formation of the bond between the ^{13}C and ^{15}N labelled sites (ring closed structure) produces a more shielded environment giving a shielded shift of 94.7 ppm for compound **4**. Compound **6** displays isotropic

shifts at both 82.8 and 87.6 ppm. The observation of two resonances corroborates with the previous diffraction data showing two crystallographically distinct sites in the unit cell. The shifts are consistent with the formation of N-C bond and breaking of the double bond. Crystallography suggests that the degree of bond formation in **6** is higher than in **4**, hence the more shielded shift.

The ^{1}H-^{15}N CPMAS NMR spectra show an expected opposite result to the achieved ^{1}H-^{13}C CPMAS NMR data. Isotropic shifts of −340.8 and −333.1 ppm (shift reference against $MeNO_2$, to convert to the protein chemical shift value add 379.5 ppm) for the compounds **2** and **3** respectively, which shifts to −290.9 and −292.2 ppm for the compounds **4** and **6**, respectively. The observed increase in the chemical shift can be attributed to lone pair electron density being donated from the ^{15}N site to form the closed ring structure and the resultant positive charge on nitrogen.

Table S17: A comparison of the bond distance, electron density, experimental solid state NMR, and the DFT derived NMR parameters.

Sample	Charge Density			Experimental NMR		CASTEP			
	R_{ij} (N---C) (Å)	ρ(r)	$\nabla^2\rho$ (r)	δ_{iso} (^{15}N) Ppm	δ_{iso} (^{13}C) Ppm	R_{ij} (N---C) (Å)	$^{1}J_{NC}$ (Hz)	δ_{iso} (^{15}N) Ppm	δ_{iso} (^{13}C) ppm
**1**	2.676	0.130	1.688	-	-	2.676	0.85	-328	157
**2**	2.480	0.192	1.971	-341	188	2.480	0.48	-335	191
**3**	2.416	0.213	2.199	-337	167	2.416	0.45	-318	166
Senkirkine,S**1**	2.245	0.332*	2.262*	-	-	2.245	0.61	-314	183
Clivorine, S**2**	1.993	0.564*	2.129*	-	-	1.993	2.32	-296	177
**4**	1.647	1.195	-2.924	-295	94	1.647	5.88	-285	93
**5**	1.624	1.258	-3.461	-	-	1.624	4.01	-282	89
**6**	1.625	1.294	-4.300	-292	88	1.625	4.73	-290	86
	1.607	1.347	-6.731	-292	83	1.607	3.57	-287	82

*charge density and Laplacians for **S1** and **S2** are calculated, not experimental.

5. References.

[S1] a) H. E. Fierz-David, L. Blangey, *Organic Chemistry 3rd Edn by L .F. Fieser and M. Fieser*, Reinhold Publishing Corporation, **1956**, p. 739; b) P. C. Bell, J. D. Wallis, *Chem. Commun*. **1999**, 257-258; c) A. J. Kirby, J. M. Percy, *Tetrahedron* **1988**, *44*, 6903-6910.

[S2] A. Lari, M .B. Pitak, S. J. Coles, G. J. Rees, S. P. Day, M. E. Smith, J. V. Hanna, J. D. Wallis, *Org. Biomolec. Chem*. **2012**, *10*, 7763-7779.

[S3] R. F. W. Bader, *Atoms in Molecules - A Quantum Theory*, Oxford University Press, Oxford, 1990.

[S4] a) P. R. Mallinson, G. T. Smith, C. C. Wilson, E. Grech, K. Wozniak, *J. Am. Chem. Soc.* **2003**, *125*, 4259-4270; b) S. Cenedese, V. K. Zhurov, A. A. Pinkerton, *Cryst. Grow. Des*. **2015**, *15*, 875-883; c) M. S. Pavan, K. D. Prasad, T. N. Guro Row, *Chem. Commun*. **2013**, *49*, 7558-7560; d) I. L. Kirby, M. Brightwell, M. B. Pitak, C. Wilson, S. J. Coles, P. A. Gale, *PCCP* **2014**, *16*, 10943-10958; e) I. L. Kirby, M. B. Pitak, C. Wilson, S. J. Coles, P. A. Gale *CrystEngComm* **2015**, *17*, 2815-2826.

[S5] R. Hooft, B.V. Nonius: Delft: Netherlands, **1998**.

[S6] P. A. Gale, S. J. Coles, *Chem. Sci.* **2012**, *3*, 683-689.

[S7] Rigaku Corporation, Japan.

[S8] Bruker AXS Inc., 5465 East Cheryl Parkway, Madison, WI 53711-5373, USA.

[S9] A. J. M. Duisenberg, L. M. J. Kroon-Batenburg, A. M. M. Schreurs, *J. Appl. Crystallog.* **2003**, *36*, 220-229.

[S10] R. H. Blessing, *J. Appl. Crystallog.* **1997**, *30*, 421-426.

[S11] G. M. Sheldrick, *Acta Crystallogr. Sect. C* **2015**, *71*, 3-8.

[S12] A. Ø. Madsen, A. A. Hoser, *J. Appl. Crystallogr.* **2014**, *47*, 2100-2104.

[S13] N. K. Hansen, P. Coppens, *Acta Crystallogr. Sect. A* **1978**, *34*, 909-921.

[S14] A. Volkov, P. Macchi, L. J. Farrugia, C. Gatti, P. Mallinson, T. Richter, T. Koritsanszky, XD2016: A Computer Program Package for Multipole Refinement, Topological Analysis of Charge Densities and Evaluation of Intermolecular Energies from Experimental and Theoretical Structure Factors, **2016,** Buffalo, USA, http://www.chem.gla.ac.uk/~louis/xd-home/.

[S15] F. H. Allen, I. J. Bruno, *Acta Crystallogr. Sect. B* **2010**, *66*, 380-386.

[S16] a) K. Meindl, J. Henn, *Acta Crystallogr. Sect. A* **2008**, *64*, 404-418; b) J. Henn, A. Schönleber, *Acta Crystallogr. Sect. A* **2013**, *69*, 549-558; c) J. Henn, K. Meindl, *Acta Crystallogr. Sect. A* **2014**, *70*, 248-256.

[S17] Gaussian 09, (Windows version 9.0), M. J. Frisch, G. W. Trucks, H. B. Schlegel, G. E. Scuseria, M. A. Robb, J. R. Cheeseman, G. Scalmani, V. Barone, B. Mennucci, G. A. Petersson, H. Nakatsuji, M. Caricato, X. Li, H. P. Hratchian, A. F. Izmaylov, J. Bloino, G. Zheng, J. L. Sonnenberg, M. Hada, M. Ehara, K. Toyota, R. Fukuda, J. Hasegawa, M. Ishida, T. Nakajima, Y. Honda, O. Kitao, H. Nakai, T. Vreven, J. A. Montgomery, Jr., J. E. Peralta, F. Ogliaro, M. Bearpark, J. J. Heyd, E. Brothers, K. N. Kudin, V. N. Staroverov, R. Kobayashi, J. Normand, K. Raghavachari, A. Rendell, J. C. Burant, S. S. Iyengar, J. Tomasi, M. Cossi, N. Rega, J. M. Millam, M. Klene, J. E. Knox, J. B. Cross, V. Bakken, C. Adamo, J. Jaramillo, R. Gomperts, R. E. Stratmann, O. Yazyev, A. J. Austin, R. Cammi, C. Pomelli, J. W. Ochterski, R. L. Martin, K. Morokuma, V. G. Zakrzewski, G. A. Voth, P. Salvador, J. J. Dannenberg, S. Dapprich, A. D. Daniels, Ö. Farkas, J. B. Foresman, J. V. Ortiz, J. Cioslowski, and D. J. Fox, Gaussian, Inc., Wallingford CT, 2009.

[S18] a) C. Lee, W. Yang, R. G. Parr, *Phys. Rev. B.* **1988**, *37*, 785-789; b) A. D. Becke, *J. Chem. Phys.* **1993**, *98*, 5648-5652; c) P. C. Hariharan, J. A. Pople, *Theor. Chim. Acta* **1973**, *28*, 213-222.

[S19] AIMAll (Version 17.11.14), K. A. Todd, TK Gristmill Software, Overland Park KS, USA, 2016 (aim.tkgristmill.com); AIMA2000, F. Biegler-König, J. Schönbohm, *J. Comp. Chem*. **2002**, *23*, 1489-1494.

[S20] R. K. Harris, E. D. Becker, S. M. Cabral de Menezes, R. Goodfellow, P. Granger, *Mag. Res. Chem.* **2002**, *40*, 489-505.

[S21] I. Hung, A.-C. Uldry, J. Becker-Baldus, A. L. Webber, A. Wong, M. E. Smith, S. A. Joyce, J. R. Yates, C. J. Pickard, R. Dupree, S. P. Brown, *J. Am. Chem. Soc.* **2009**, *131*, 1820-1834.

[S22] D. Massiot, F. Fayon, M. Capron, I. King, S. Le Calve, B. Alonso, J. O. Durand, B. Bujoli, Z. H. Gan, G. Hoatson, *Mag. Res. Chem.* **2002**, *40*, 70-76.

[S23] M. Bak, J. T. Rasmussen, N. C. Nielsen, *J. Mag. Res.* **2000**, *147*, 296-330.

[S24] G. J. Rees, S. P. Day, K. E. Barnsley, D. Iuga, J. R. Yates, J. D. Wallis, J. V. Hanna, *PCCP* **2020**, *22*, 3400-3413.

[S25] S. J. Clark, M. D. Segall, C. J. Pickard, P. J. Hasnip, M. J. Probert, K. Refson, M. C. Payne, *Z. Kristallogr.* **2005**, *220*, 567-570.

[S26] J. P. Perdew, J. A. Chevary, S. H. Vosko, K. A. Jackson, M. R. Pederson, D. J. Singh, C. Fiolhais, *Phys. Rev. B.* **1992**, *46*, 6671-6687; J. P. Perdew, J. A. Chevary, S. H. Vosko, K. A. Jackson, M. R. Pederson, D. J. Singh, C. Fiolhais, *Phys. Rev. B.* **1993**, *48*, 4978-4978.

[S27] C. J. Pickard, F. Mauri, *Phys. Rev. B.* **2001**, *63*, 245101.
